# E–H Bond Cleavage Processes in Reactions of Heterometallic Phosphinidene-Bridged MoRe and MoMn Complexes with Hydrogen and p-Block Element Hydrides

**DOI:** 10.1021/acs.organomet.3c00295

**Published:** 2023-08-31

**Authors:** M. Angeles Alvarez, M. Esther García, Daniel García-Vivó, Miguel A. Ruiz, Patricia Vega

**Affiliations:** Departamento de Química Orgánica e Inorgánica/IUQOEM, Universidad de Oviedo, E-33071 Oviedo, Spain

## Abstract

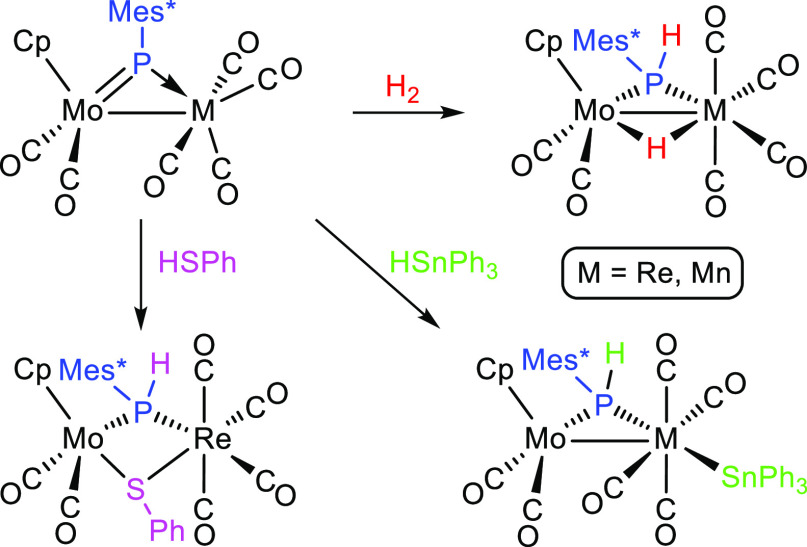

Reactions of complexes [MoMCp(μ-PMes*)(CO)_6_] with H_2_ and several p-block element (E) hydrides mostly resulted in the cleavage of E–H bonds under mild conditions [M = Re (**1a**) and Mn (**1b**); Mes* = 2,4,6-C_6_H_2_^*t*^Bu_3_]. The reaction with H_2_ (ca. 4 atm) proceeded even at 295 K to give the hydrides [MoMCp(μ-H)(μ-PHMes*)(CO)_6_]. The same result was obtained in the reactions with H_3_SiPh and, for **1a**, upon reduction with Na(Hg) followed by protonation of the resulting anion [MoReCp(μ-PHMes*)(CO)_6_]^−^. The latter reacted with [AuCl{P(*p*-tol)_3_}] to yield the related heterotrimetallic cluster [MoReAuCp(μ-PHMes*)(CO)_6_{P(*p*-tol)_3_}]. The reaction of **1a** with thiophenol gave the thiolate-bridged complex [MoReCp(μ-PHMes*)(μ-SPh)(CO)_6_], which evolved readily to the pentacarbonyl derivative [MoReCp(μ-PHMes*)(μ-SPh)(CO)_5_]. In contrast, no P–H bond cleavage was observed in reactions of complexes **1a,b** with PHCy_2_, which just yielded the substituted derivatives [MoMCp(μ-PMes*)(CO)_5_(PHCy_2_)]. Reactions with HSnPh_3_ again resulted in E–H bond cleavage, but now with the stannyl group terminally bound to M, while **1a** reacted with BH_3_·PPh_3_ to give the hydride-bridged derivatives [MoReCp(μ-H)(μ-PHMes*)(CO)_5_(PPh_3_)] and [MoReCp(μ-H){μ-P(CH_2_CMe_2_)C_6_H_2_^*t*^Bu_2_}(CO)_5_(PPh_3_)], which follow from hydrogenation, C–H cleavage, and CO/PPh_3_ substitution steps. Density functional theory calculations on the PPh-bridged analogue of **1a** revealed that hydrogenation likely proceeds through the addition of H_2_ to the Mo=P double bond of the complex, followed by rearrangement of the Mo fragment to drive the resulting terminal hydride into a bridging position.

## Introduction

**Chart 1:**
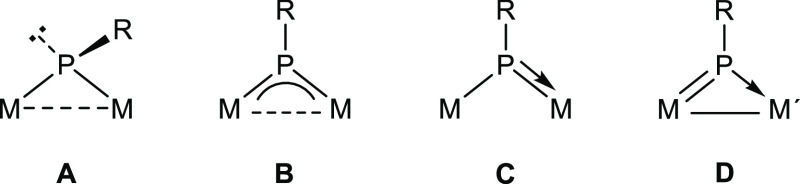
Coordination modes of PR ligands at binuclear complexes.

**Scheme 1:**
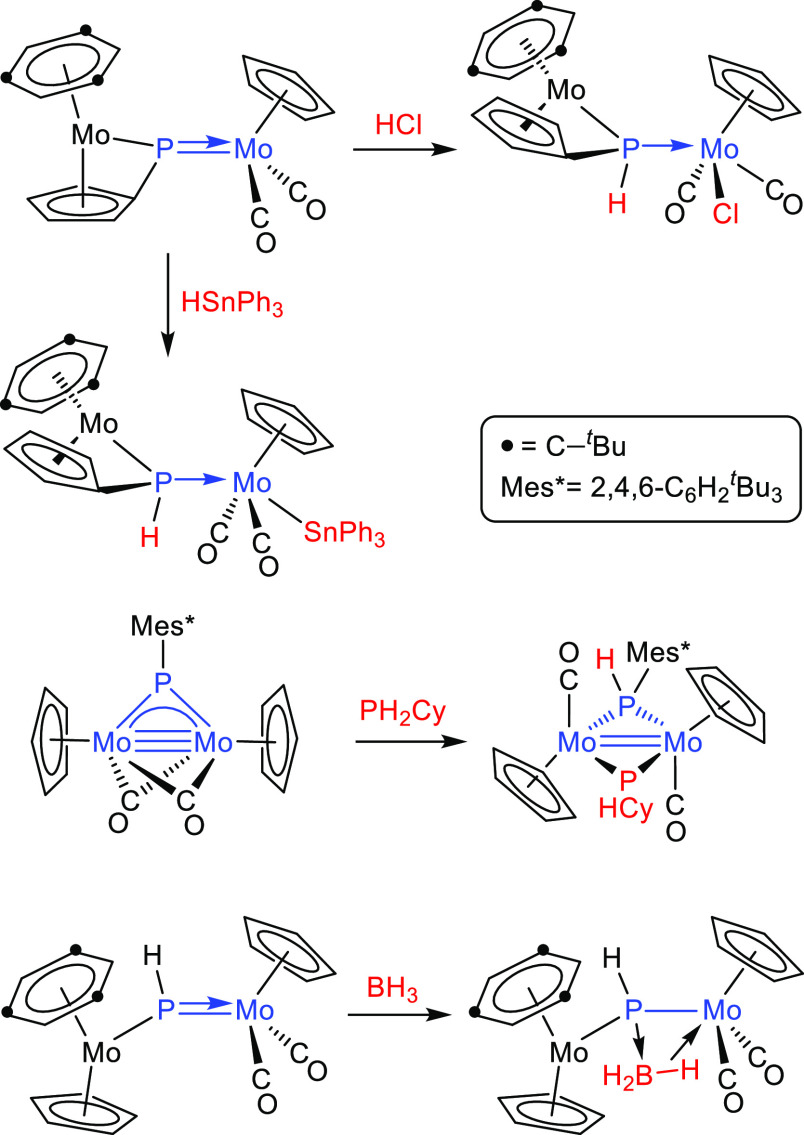
E–H Bond Activation with Mo_2_ Complexes of Types B and C

The activation and eventual cleavage of single bonds between hydrogen and p-block elements (E) of simple molecules HER_n_ (R = H, halogen, and hydrocarbyl group) is a central matter in molecular chemistry, with a significant impact on both organic (olefin hydrogenation, hydrosilylation, hydrophosphination, and related reactions) and inorganic reactions, the most representative example in the latter case being the oxidative addition reaction to metal complexes. These processes are particularly difficult when E is hydrogen itself since the dihydrogen molecule displays a quite strong bond with no polarity. Different strategies have proved to be useful to promote the cleavage of E–H bonds on homogeneous media. One of the best studied processes is the activation of such bonds by coordination to unsaturated metal complexes because the latter provide both an acceptor molecular orbital for attachment of the external reagent to the metal site, via the σ(E–H) bonding orbital, and a nonbonding filled orbital to populate the corresponding σ*(E–H) antibonding orbital (back-donation). This enables the formation of a σ-complex with a weakened E···H bond, most commonly evolving through its full cleavage and eventually resulting in the oxidative addition to the metal atom.^1^ Suitable acceptor and donor orbitals to cleave H–H and H–E bonds are also found in other inorganic molecules as those displaying E–E multiple bonds, such as the heavier analogues of alkynes,^2^ or complexes having M–E multiple bonds, including the ones found in trigonal phosphanide (M = PR_2_),^3^ and terminal bent phosphinidene (M = PR) complexes,^4^ among others. Phosphinidene-bridged binuclear complexes, on the other hand, also display M–P multiple bonding in several of their possible coordination modes (**A** to **D** in Chart 1), although their reactions toward H–ER_n_ molecules have been scarcely studied, actually limited to a few homometallic complexes of types **A** to **C**. These have been reviewed previously by us and will not be discussed in detail here.^5,6^ We just note that the addition of H–E bonds across M–P multiple bonds is a typical output of these reactions for complexes of types **B** and **C**, with specific formation of P–H bonds, irrespective of the polarity (positive or negative) of the H atom in the added reagent, as shown by the reactions with HCl,^7^ PH_2_Cy,^8^ and HSnPh_3_^6^ shown in Scheme 1, although the opposite regiochemistry can be grasped in the reaction of [Mo_2_Cp_2_(μ-PH)(CO)_2_(η^6^-Mes*H)] with BH_3_·THF, which, however, led to a phosphinidene-borane bridged complex without B–H bond cleavage (Mes* = 2,4,6-C_6_H_2_^*t*^Bu_3_).^9^

**Scheme 2:**
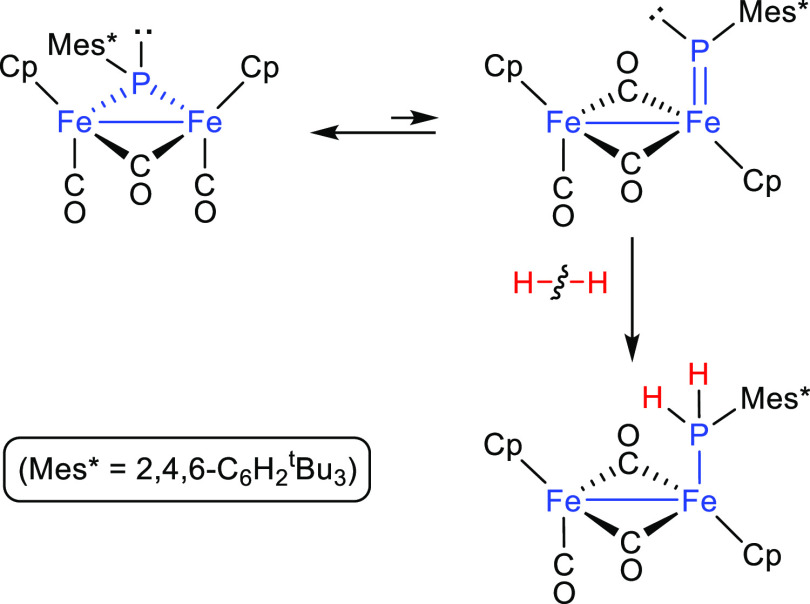
Hydrogenation Reaction of [Fe_2_Cp_2_(μ-PMes*)(μ-CO)(CO)_2_]

At first sight, complexes of type **A** (with pyramidal phosphinidene ligands) would fail to fulfill the orbital requirements mentioned above to promote the E–H bond cleavage. Thus, it was surprising to find that the diiron complex [Fe_2_Cp_2_(μ-PMes*)(μ-CO)(CO)_2_] reacted at room temperature with H_2_ (ca. 4 atm) to yield the phosphine complex [Fe_2_Cp_2_(μ-CO)_2_(CO)(PH_2_Mes*)], thus providing the first example of hydrogenation of a P-containing species under such mild conditions, yet unbeaten. A density functional theory (DFT) study, however, suggested that the actual species being hydrogenated might be an isomer of the above complex displaying a terminal PR ligand with a Fe = P double bond, which would be present in solution as a minor species in equilibrium with the major, PR-bridged isomer (Scheme 2).^10^ Thus, it might be concluded that the presence of M–P multiple bonding certainly is a favoring element for the activation of H–E bonds in these sorts of complexes.

**Chart 2:**
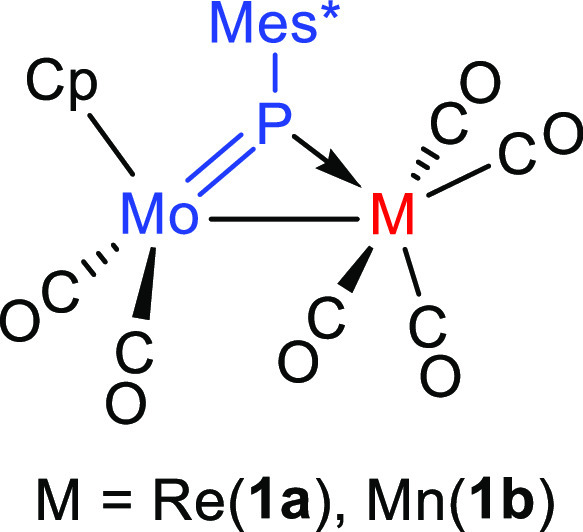
Structure of compounds **1a,b**.

Recently, an efficient preparative route for the heterometallic compound [MoReCp(μ-PMes*)(CO)_6_] (**1a**, Chart 2) was implemented by us.^11^ This complex is the first recognized example of a type **D** phosphinidene complex where, in spite of the isoelectronic nature of the metal fragments involved (15 electrons, in that case), the metal–phosphorus π-bonding interaction is essentially located at one of the M–P connections. Previous studies on the reactivity of **1a** indicated a marked trend of this complex to undergo cycloaddition processes at its Mo = P double bond when reacting with unsaturated organic molecules,^11,12^ which therefore seemed an excellent candidate for inspecting its ability to activate and cleave single E–H bonds. In this paper, we analyze the reactivity of **1a**, and that of its manganese analogue [MoMnCp(μ-PMes*)(CO)_6_] (**1b**),^13^ toward hydrogen and some simple H–ER_n_ molecules such as thiols, secondary phosphines, silanes, stannanes, and boranes. As is shown below, several examples of E–H bond cleavage processes are observed in these reactions under mild conditions, with specific formation of P–H and M–E bonds. Compounds **1** were even able to cleave the strong bond of H_2_ under mild conditions (room temperature, ca. 4 atm H_2_ pressure) in a process likely involving a two-step addition of this molecule to the Mo=P double bond of these phosphinidene complexes, according to DFT calculations.

## Results and Discussion

### Hydrogenation and Related Reactions of Compounds **1**

**Scheme 3:**
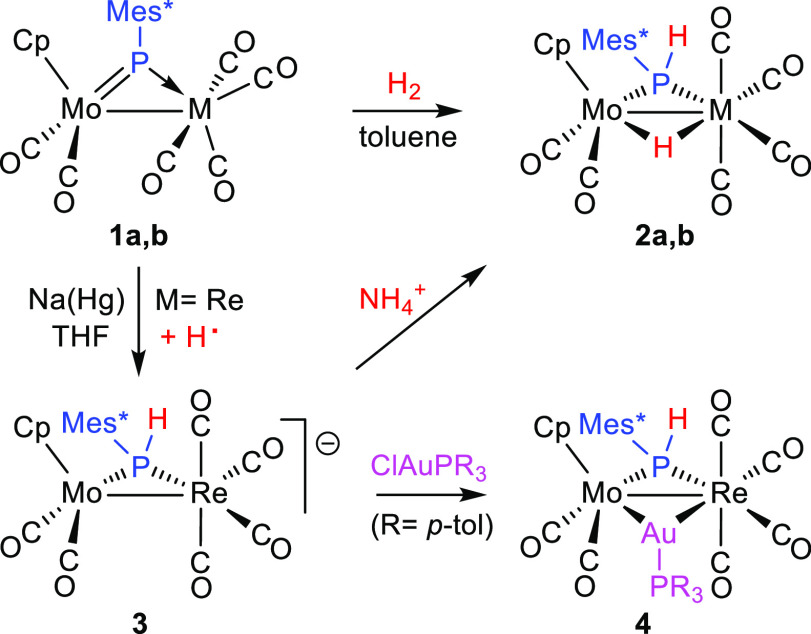
Hydrogenation and Related Derivatives of Compounds **1**

The rhenium complex **1a** reacts with H_2_ (ca. 4 atm) in toluene solution to give the phosphanide- and hydride-bridged complex [MoReCp(μ-H)(μ-PHMes*)(CO)_6_] (**2a**) selectively (Scheme 3). This reaction is completed in ca. 18 d at room temperature and in ca. 3 h at 363 K. The manganese complex **1b** reacts with H_2_ in a similar way to give the corresponding hydride complex [MoMnCp(μ-H)(μ-PHMes*)(CO)_6_] (**2b**), but the rate is faster as the reaction is now completed in ca. 3 d at room temperature, thus approaching the hydrogenation rate of the diiron complex depicted in Scheme 2 (ca. 16 h at room temperature and ca. 4 atm H_2_ pressure).^10^ This is a remarkable result as no isomer bearing a terminal PMes* ligand appears to be involved here, as it seems to be in the diiron case, and therefore would provide the first example of a genuine dihydrogen addition taking place at a phosphinidene-bridged complex. Details of the likely pathway for these unusual reactions are discussed later on in the light of DFT calculations on possible intermediates and transitions states. We should remark that these rates largely exceed that of the only reported hydrogenation of a terminal phosphinidene complex we are aware of, the transient tungsten complex [W(CO)_5_(PPh)], found to be hydrogenated at 423 K and 20 atm H_2_ pressure to yield [W(CO)_5_(PH_2_Ph)] in ca. 4 h.^14^

Hydrogenation also occurs surprisingly upon reduction of the MoRe complex **1a** with Na(Hg) in tetrahydrofuran solution, which seems to proceed with spontaneous H atom abstraction at the P atom of the putative radical intermediate [MoReCp(μ-PMes*)(CO)_6_]^−^ following one-electron reduction to give the Na^+^ salt of the phosphanide-bridged anion [MoReCp(μ-PHMes*)(CO)_6_]^−^ (**3**) (Scheme 3). This output, in any case, is consistent with the electronic structure of **1a**, with a LUMO having π*(Mo–P) antibonding character and large participation of a P atomic orbital.^11a^ The latter makes the P atom a likely location for an added electron and, therefore, a probable site for initial attachment of an abstracted H atom.^15^ The hydrogen source in this reaction has not been identified but likely is trace water present in the solvent.

Although the Na^+^ salt of the anionic complex **3** was not isolated, its formulation is firmly supported by its IR spectrum, with C–O stretches some 100 cm^–1^ less energetic, on average, than those of its neutral hydride derivative **2a** (Table 1), while the presence of a phosphanide-bridged ligand with a P–H bond is denoted by the dramatic shielding of its ^31^P NMR resonance, compared to the parent complex (from ca. 673 to 41 ppm), and the appearance of a large one-bond coupling to a single H atom (^1^*J*_PH_ = 329 Hz). These parameters are comparable to those of the hydride complex **2a** (δ_P_ = 1.4 ppm, ^1^*J*_PH_ = 343 Hz). We note that similar anionic complexes [MoMCp(μ-PPh_2_)(CO)_6_]^−^ have been reported as being formed through deprotonation of the corresponding neutral hydrides [MoMCp(μ-H)(μ-PPh_2_)(CO)_6_] (M = Re^16^ and Mn).^17^ As expected, anion **3** reacted with NH_4_PF_6_ to give the corresponding hydride-bridged derivative **2a** in good yield. An analogous reaction takes place with the gold(I) complex [AuClP(*p*-tol)_3_] to give the heterotrimetallic cluster [MoReAuCp(μ-PHMes*)(CO)_6_{P(*p*-tol)_3_}] (**4**) (Scheme 3).

**Table 1:** Selected IR and ^31^P NMR Data for New Compounds^a^

compound	ν(CO)	δ (P) [^1^*J*_PH_]
[MoReCp(μ-PMes*)(CO)_6_] (**1a**)^b^	2077 (m), 1986 (vs), 1951 (s), 1927 (w, sh), 1876 (w)	673.1
[MoMnCp(μ-PMes*)(CO)_6_] (**1b**)^c^	2055 (m), 2039 (w), 1974 (vs), 1951 (s), 1888 (w), 1862 (w)	720.9
[MoReCp(μ-H)(μ-PHMes*)(CO)_6_] (**2a**)	2088 (m), 1994 (s, sh), 1983 (vs), 1956 (f), 1881 (m)	1.4 [343]
[MoMnCp(μ-H)(μ-PHMes*)(CO)_6_] (**2b**)	2069 (s), 1995 (s), 1977 (vs), 1958 (vs), 1882 (m)	68.2 [336]
Na[MoReCp(μ-PHMes*)(CO)_6_] (**Na-3**)	2030 (m), 1935 (vs), 1886 (s), 1874 (m), 1795 (m), 1753 (m)^d^	40.8 [329]^d^
[MoReAuCp(μ-PHMes*)(CO)_6_{P(*p*-tol)_3_}] (**4**)	2049 (m), 1962 (s), 1951 (vs), 1931 (vs), 1845 (m)^e^	67.8, 64.9 [341]^f^
[MoReCp(μ-PHMes*)(μ-SPh)(CO)_6_] (**5**)	2091 (m), 1997 (f), 1982 (f), 1952 (mf), 1875 (m)	–233.8 [304]
*syn*-[MoReCp(μ-PHMes*)(μ-SPh)(CO)_5_] (*syn***-6**)	2019 (vs), 1988 (m), 1929 (m), 1906 (m)	–8.1 [352]
*anti*-[MoReCp(μ-PHMes*)(μ-SPh)(CO)_5_] (*anti***-6**)	2019 (vs), 1988 (m), 1929 (m), 1906 (m)	–33.4 [368]
[MoReCp(μ-PMes*)(CO)_5_(PHCy_2_)] (**7a**)	2027 (w), 1938 (vs), 1913 (m), 1890 (m), 1839 (w)	706.9 (92), 10.6 [339]
[MoMnCp(μ-PMes*)(CO)_5_(PHCy_2_)] (**7b**)	2007 (w), 1934 (vs), 1911 (s), 1897 (m, sh), 1847 (w)	760.0 (br), 58.4 [325]
*trans*-[MoReCp(μ-PHMes*)(CO)_6_(SnPh_3_)] (**8a**)	2064 (vw), 2010 (w), 1968 (vs), 1941 (m), 1880 (m)^e^	90.7 [348]^e^,^g^
*trans*-[MoMnCp(μ-PHMes*)(CO)_6_(SnPh_3_)] (**8b**)	2062 (vw), 2038 (w), 1959 (vs), 1950 (s, sh), 1935 (m), 1883 (m)^e^	144.3 [341]^e^
*cis*-[MoMnCp(μ-PHMes*)(CO)_6_(SnPh_3_)] (**9**)	2054 (s), 1990 (m), 1958 (vs), 1950 (s), 1918 (m), 1840 (m)^e^	89.5 [345]^e^
[MoReCp(μ-H)(μ-PHMes*)(CO)_5_(PPh_3_)] (**10**)	2038 (w), 1950 (vs), 1938 (s), 1917 (m), 1866 (m)	12.8 (85), 11.5 [345]
[MoReCp(μ-H){μ-P(CH_2_CMe_2_)C_6_H_2_^*t*^Bu_2_}(CO)_5_(PPh_3_)] (**11**)	2037 (w), 1946 (vs), 1935 (s, sh), 1913 (m), 1865 (m)	91.4 (84), 11.6

a IR spectra recorded in dichloromethane solution; ^31^P{^1^H} and ^31^P NMR spectra recorded in CD_2_Cl_2_ solution at 121.48 MHz and 293 K, with chemical shifts (δ) in ppm relative to external 85% aqueous H_3_PO_4_ and coupling constants (*J*) in hertz; ^1^*J*_PH_ data given between square brackets, taken from the corresponding ^31^P or ^1^H NMR spectra (see the Experimental Section), and *J*_PP_ data given between brackets.

b Data taken from ref (11a).

c Data taken from ref (13).

d In tetrahydrofuran.

e In toluene.

f In benzene-*d*_6_.

g *J*(P–^119^Sn)∼*J*(P–^117^Sn) = 121 Hz.

Spectroscopic data for compounds **2a,b** (Table 1) are similar to each other, except for the expected differences when replacing Re with Mn in isostructural couples (a slight decrease in the C–O stretching frequencies^18^ and a significant increase of the ^31^P chemical shift).^19^ The high intensity of the most energetic C–O stretch in each case denotes the presence of a M(CO)_4_ fragment with disphenoidal geometry,^18^ and the overall pattern of the spectrum is comparable to those of related complexes of type [MoMCp(μ-H)(μ-PR_2_)(CO)_6_] reported previously (M = Re, R = Ph and Cy;^16b,20^ M = Mn, R = Ph).^21^ The hydride ligands of compounds **2a,b** give rise to strongly shielded resonances at ca. −14 ppm, weakly coupled to the P atom (^2^*J*_PH_ ca. 20–30 Hz, metal sensitive), as expected for bridging hydrides, while the P-bound H atoms give rise to quite deshielded resonances at ca. 7.50 ppm, strongly coupled to the ^31^P nucleus (^1^*J*_PH_ ca. 340 Hz, metal insensitive), as noted above.

### Structure of the Heterotrimetallic Cluster **4**

**Figure 1:**
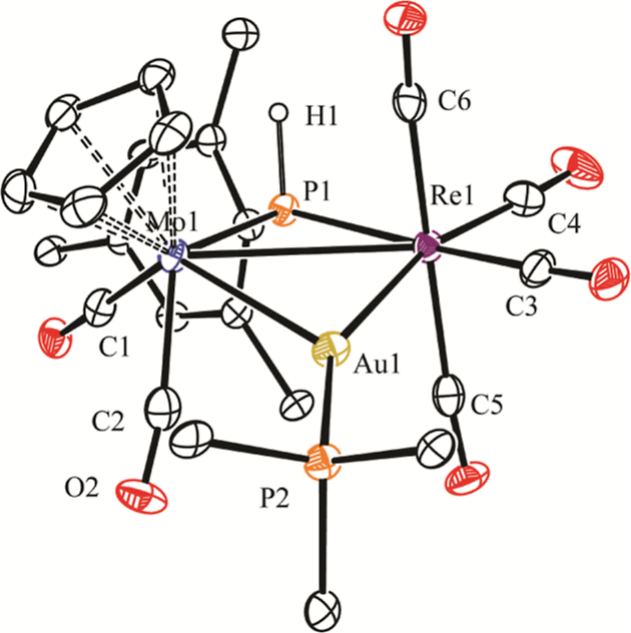
ORTEP diagram (30% probability) of compound **4**, with ^*t*^Bu and *p*-tol groups (except their C^1^ atoms) and most H atoms omitted for clarity.

The structure of **4** in the crystal (Figure 1 and Table 2) displays a central triangular MoReAu core built from cisoid MoCp(CO)_2_, disphenoidal Re(CO)_4_, and AuPR_3_ fragments, with a PHMes* ligand bridging Mo and Re atoms in a symmetrical way (M–P ca. 2.44 Å). The conformation of the latter ligand is the one having the P-bound H atom and the Cp ligand on the same side of the MoReAu plane, likely more favored on steric grounds because this allows the bulky Mes* group to point away from the Cp and other ligands of the molecule, to minimize steric repulsions. Presumably, this is also the conformation present in the related hydride-bridged complexes **2**. We note that no other cluster with a triangular MoReAu core appears to have been crystallographically characterized previously.

**Table 2:** Selected Bond Lengths (Å) and Angles (deg) for Compound **4**

Mo1–Re1	3.3256(6)	Mo1–Au1–Re1	72.48(1)
Mo1–Au1	2.8645(5)	Mo1–P1–Re1	85.70(4)
Mo1–P1	2.443(1)	Mo1–Au1–P2	132.45(4)
Mo1–C1	1.954(6)	Re1–Au1–P2	155.03(4)
Mo1–C2	1.992(7)	P1–Mo1–C1	78.4(2)
Re1–Au1	2.7591(4)	P1–Mo1–C2	115.1(2)
Re1–P1	2.448(1)	P1–Re1–C3	170.9(2)
Re1–C3	1.964(6)	P1–Re1–C4	93.5(2)
Re1–C4	1.953(8)	P1–Re1–C5	100.2(2)
Re1–C5	2.012(7)	P1–Re1–C6	85.9(2)
Re1–C6	1.997(7)	C1–Mo1–C2	76.0(2)
Au1–P2	2.312(1)	C3–Re1–C4	93.3(3)
P1–H1	1.30(5)	C5–Re1–C6	171.1(2)

By using the well-known H/AuPR_3_ isolobal analogy,^22^ we can view cluster **4** as an electron-precise molecule, as is also the case of complexes **2**, and therefore, a single Mo–Re bond is to be proposed according to the 18-electron rule.^23^ This is consistent with the corresponding intermetallic separation of 3.3256(6) Å, which, however, falls a bit above the usual range of 2.88–3.20 Å found for Mo–Re single-bond distances in carbonyl complexes.^24^ For comparison, we note that this length is some 0.15 Å longer than those determined for the unbridged complex [MoReCp(CO)_8_] (3.172(1) Å)],^25^ or in the more closely related phosphanide- and hydride-bridged complexes [MoReCp(μ-H)(μ-PPh_2_)(CO)_6_] (3.188(1) Å)^16b^ and [MoReCp(μ-H)(μ-PCy_2_)(CO)_5_(NH_3_)] (3.1876(3) Å).^26^ Noticeably, the Mo–Re distance in **4** is somewhat longer than those determined in related Mo_2_Au (Mo–Mo = 3.238(1) Å in [Mo_2_AuCp(μ-PPh_2_)(CO)_6_(PPh_3_)]),^27^ and Re_2_Au clusters {Re–Re = 3.261(2) Å in [Re_2_AuCp(μ-PHCy)(CO)_6_(PPh_3_)]},^28^ a difference that might be due to the steric pressure introduced by the bulky Mes* substituent at the bridging phosphanide ligand of **4**. Besides this, we note that even if the M–Au lengths in **4** are comparable to those measured in the mentioned clusters, we can describe the gold atom in **4** as being positioned closer to the Re atom because the difference in the corresponding M–Au distances (ca. 0.1 Å) exceeds the difference in the covalent radii of Mo and Re atoms (ca. 0.03 Å).^29^ In addition to this, the Re–Au–P angle is significantly larger than the Mo–Au–P one (ca. 155 vs 132°). It is not obvious whether this distortion follows from steric or electronic differences between the MoCp(CO)_2_ and Re(CO)_4_ fragments of the cluster, but it might be viewed as a positioning of the AuPR_3_ fragment on its way from symmetrical bridging to a terminal arrangement, relative to the MoRe center.

Spectroscopic data in solution for compound **4** (Table 1 and Experimental Section) are consistent with its solid-state structure and deserve only a few comments. Its IR spectrum displays a pattern comparable to those of the hydride-bridged complexes **2a,b**, but with C–O stretching frequencies significantly lower (by ca. 30 cm^–1^), as usually observed when replacing bridging H atoms with AuPR_3_ groups,^30^ and its ^31^P NMR spectrum displays two close and mutually uncoupled resonances at ca. 65 ppm due to the AuPR_3_ and μ-PHMes* groups. The latter can be easily identified by its strong coupling (^1^*J*_PH_ = 341 Hz) to one H atom (δ_H_ 8.03 ppm) and has a chemical shift a bit higher than that of its anionic precursor **Na-3**, whereas the chemical shift of the gold-bound phosphine is unremarkable.

### Reactions of Compounds **1** with Thiols and Secondary Phosphines

**Scheme 4:**
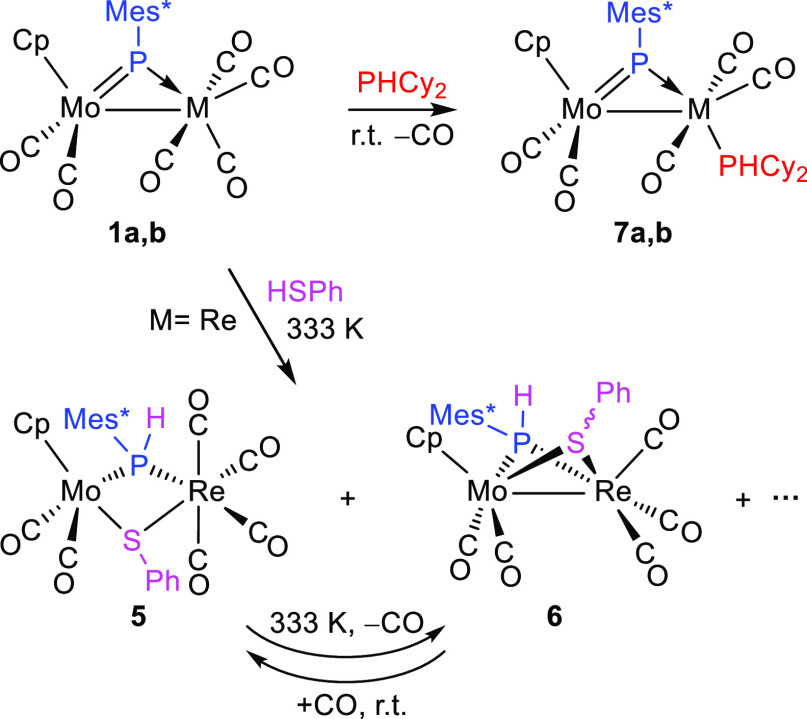
Reactions of Compounds **1** with HSPh and PHCy_2_

In contrast to hydrogen, thiols and secondary phosphines bear lone electron pairs at the S or P atoms, enabling their coordination at an unsaturated metal center, particularly in the case of phosphines, while the corresponding E–H bonds have some polarity, which is also a helpful feature with respect to its eventual cleavage. Indeed, although the rhenium complex **1a** failed to react with thiophenol at room temperature, it did it slowly at 333 K with low selectivity to give a mixture of the hexacarbonyl complex [MoReCp(μ-PHMes*)(μ-SPh)(CO)_6_] (**5**), its pentacarbonyl derivative [MoReCp(μ-PHMes*)(μ-SPh)(CO)_5_] (**6**), and a P-free product likely to be the dithiolate complex [MoReCp(μ-SPh)_2_(CO)_5_], not further investigated (Scheme 4).^31,32^ Increasing the reaction temperature to 363 K yielded the P-free species as the major product, and this was also the major output in the reactions of the manganese complex **1b** with thiophenol under different conditions, which, therefore, were not further explored. On the other side, separate experiments indicated that the hexacarbonyl complex **5** undergoes selective decarbonylation at 333 K to yield the pentacarbonyl **6**, a process also involving the formation of a Mo–Re single bond, while the latter complex can be carbonylated at room temperature upon addition of CO (1 atm), with destruction of the intermetallic interaction. Based on all of the above observations, we conclude that compound **5** is the first stable product of the reaction of **1a** with thiophenol, following from S–H bond cleavage, with specific formation of a P–H bond; however, compound **5** undergoes easy decarbonylation, and it transforms into the pentacarbonyl derivative **6** while still forming, thus explaining its low relative amount in the final mixture. The formation of the P-free product is obviously the result of the reaction of **6** with a second molecule of thiol, leading, after S–H bond cleavage and formation of a new P–H bond, to the release of phosphine PH_2_Mes*, the latter being detected spectroscopically in the ^31^P NMR spectra of the crude reaction mixtures.

Reactions of compounds **1a,b** with the primary phosphine PHCy_2_ expectedly proceeded more rapidly than the above ones and actually were completed in ca. 1 h at room temperature but just gave the corresponding derivatives of CO substitution [MoMCp(μ-PMes*)(CO)_5_(PHCy_2_)] [M = Re (**7a**) and Mn (**7b**)] as unique products (Scheme 4). Attempts to force the cleavage of the P–H bond present in the coordinated phosphine of these phosphinidene complexes by either heating them in boiling toluene solution or through irradiation with visible–UV light, proved unsuccessful or just led to their decomposition. This might be due to the unfavorable position (trans to PMes*) of the phosphine ligand in these complexes, far away from the phosphinidene ligand, making more difficult any possible H-transfer between P atoms. The transoid arrangement of phosphinidene and phosphine ligands in these compounds is indicated by the large two-bond P–P coupling of 96 Hz observed for **7a**, which is even larger than the coupling measured in the isoelectronic phosphanide-bridged complex *mer*-[MoReCp(μ-H)(μ-PCy_2_)(CO)_5_(PHPh_2_)] (^2^*J*_PP_ = 67 Hz).^33^ As a result of this positioning, the M-bound carbonyls in these molecules are left in a meridional or T-shaped arrangement, in agreement with corresponding IR spectra, which display their most energetic C–O stretch at ca. 2015 cm^–1^, with a weak relative intensity.^18^ These molecules thus retain the symmetry plane of the parent complexes, now containing the Mo, Re, and P atoms, as indicated in the ^13^C and ^1^H NMR spectra by the observation of degeneracy in the pertinent pairs of the CO and ^*t*^Bu resonances (see the Experimental Section).

### Structure of Thiolate Complexes **5** and **6**

**Figure 2:**
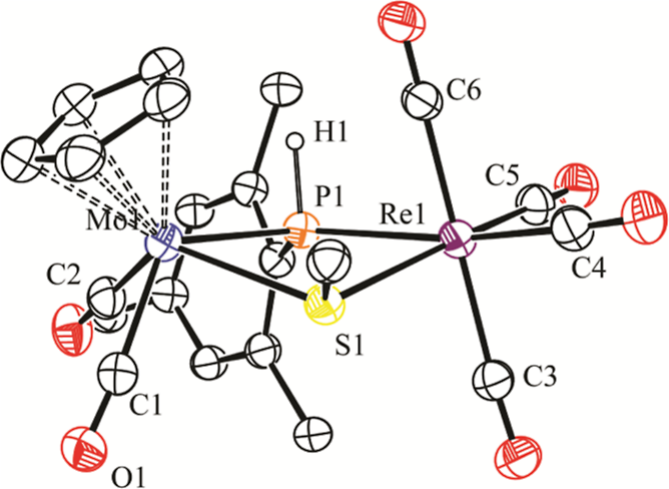
ORTEP diagram (30% probability) of compound **5**, with ^*t*^Bu and Ph groups (except their C^1^ atoms) and most H atoms omitted for clarity.

The molecule of hexacarbonyl complex **5** in the crystal (Figure 2 and Table 3) is built up from disphenoidal Re(CO)_4_ and cisoid MoCp(CO)_2_ fragments bridged by PHMes* and SPh ligands, so as to complete an octahedral environment around the Re atom and a classical four-legged piano stool environment around molybdenum. The conformation of the phosphanide ligand is comparable to the one found in cluster **4**, that is, with the bulky Mes* group as far away as possible from the Cp ligand. In contrast, the phenyl ring of the thiolate ligand adopts a syn conformation relative to the Cp ligand, perhaps to avoid the repulsive interaction with an *ortho*-^*t*^Bu group of the Mes* substituent that an anti conformation would imply. The three-electron donor nature of the bridging ligands makes this molecule a 36-electron complex, for which no metal–metal bond is to be proposed according to the 18-electron rule. In agreement with this, the intermetallic separation is quite large [4.0395(5) Å], thus precluding any significant intermetallic interaction across the somewhat puckered MoPReS central rhombus (P–Mo–Re–S ca. 152°). Incidentally, we note that this appears to be the first 36-electron MoRe or WRe complex with bridging P- and S-donor ligands to be structurally characterized. The phosphanide ligand bridges the metal atoms in a rather symmetrical way, while coordination of the thiolate ligand is slightly asymmetric, even after accounting for the small difference in the covalent radii of the metal atoms, with Mo–S and Re–S separations of 2.590(1) and 2.509(1) Å, respectively. While the latter figure is essentially identical to the Re–S distances measured in the related dirhenium complex [Re_2_(μ-PCy_2_)(μ-SPh)(CO)_8_] (ca. 2.51 Å),^34^ the Mo–S separation is significantly longer, perhaps due to the repulsions derived from the relatively close positions of Ph and Cp groups mentioned above.

**Table 3:** Selected Bond Lengths (Å) and Angles (deg) for Compound **5**

Mo1···Re1	4.0395(5)	Mo1–P1–Re1	104.56(4)
Mo1–P1	2.559(1)	Mo1–S1–Re1	104.78(3)
Mo1–S1	2.590(1)	P1–Mo1–S1	72.04(3)
Mo1–C1	1.975(4)	P1–Re1–S1	73.55(3)
Mo1–C2	1.975(4)	P1–Mo1–C1	119.7(1)
Re1–P1	2.548(1)	P1–Mo1–C2	79.3(1)
Re1–S1	2.509(1)	P1–Re1–C3	102.5(1)
Re1–C3	2.021(4)	P1–Re1–C4	169.3(2)
Re1–C4	1.949(5)	P1–Re1–C5	95.1(1)
Re1–C5	1.952(4)	P1–Re1–C6	80.6(1)
Re1–C6	1.987(4)	C1–Mo1–C2	75.9(2)
P1–H1	1.28(6)	C3–Re1–C6	174.3(2)
		C4–Re1–C5	90.8(2)

Spectroscopic data in solution for compound **5** (Table 1 and Experimental Section) are consistent with the solid-state structure just discussed and are also indicative of the presence of a single conformer in solution, presumably the one present in the crystal. Its IR spectrum displays five C–O stretches that can be identified as arising from relatively independent disphenoidal Re(CO)_4_ and cisoid Mo(CO)_2_ oscillators, with the former one being identified by a characteristic medium-intensity band at a high frequency (2091 cm^–1^). The salient spectroscopic feature of this complex is the strong magnetic shielding of its P nucleus, which displays an NMR resonance at −233.8 ppm, some 235 ppm below the one of its structurally related hydride-bridged complex **2a**, which, however, has two fewer valence electrons. This dramatic difference can be attributed to the lack of an intermetallic bond in the case of **5**.^19^ It also provides another example of the usefulness of the half-electron counting method to anticipate geometric and spectroscopic features when dealing with hydride-bridged and related binuclear carbonyl complexes of the transition metals.^23^ We also note that the one-bond P–H coupling of 304 Hz in **5** is significantly lower than the values of 330–370 Hz measured in all of the other compounds reported in this work (Table 1). This might also be related indirectly with the absence of a Mo–Re bond in **5** since this circumstance requires an opening of the Mo–P–Re angle, thus increasing the s-orbital character in the corresponding P–M bonding orbitals, which would decrease (by defect) the s-bonding character in the P–H bond and, hence, decrease the corresponding coupling constant.^35^ We have observed previously this effect in several di- and trinuclear complexes bridged by the bare P–H phosphinidene ligand, for which ^1^*J*_PH_ couplings as low as 183 Hz were measured.^36^

**Chart 3:**
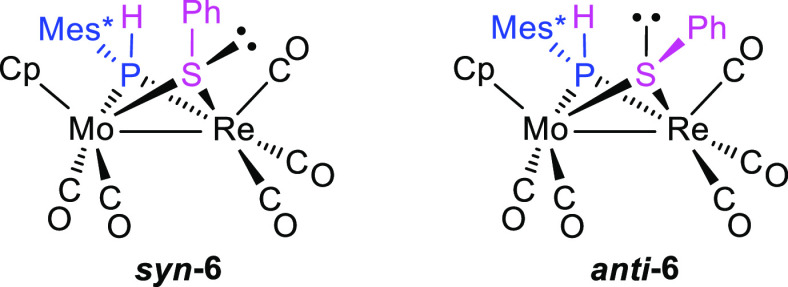
Proposed conformers for complex **6**.

Spectroscopic data for **6** are comparable to those of the structurally related, PCy_2_-bridged complex [MoReCp(μ-PCy_2_)(μ-SPh)(CO)_5_] recently reported by us^33^ and deserve only a few additional comments. The most relevant difference here stems from the NMR data, which indicate the existence in solution of two isomers in equilibrium in a ratio of ca. 3:2. Although the presence in the molecule of PHMes* and SPh bridging ligands would allow for up to four different conformers in solution, it would be sensible to assume that the conformation of the phosphanide ligand in the observed isomers would be identical to the one found for compounds **4** and **5** in the solid state, that is, the one with the bulky Mes* group away from the Cp ligand, more favored on steric grounds. This leaves only two possible structures for the isomers actually observed, differing in the disposition of the phenyl ring (syn or anti) relative to that of the Cp ligand (Chart 3). The syn conformation actually would be the one determined for **5** in the solid state, but in the case of **6**, such a conformation is expected to be somewhat destabilized since the appearance of the intermetallic interaction is accompanied by a pronounced puckering of the MoPReS central ring [P–Mo–Re–S ca. 97° in the mentioned PCy_2_-bridged complex; Mo–Re = 2.9702(8) Å], which has the effect of taking the phenyl ring closer to the Cp ligand. As a result, the energy of *syn***-6** might approach that of *anti***-6**, thus enabling their coexistence in solution. Isomer *syn***-6** still is the major isomer and can be identified by its anomalously shielded P-bound H atom, which gives rise to a NMR resonance at 4.17 ppm, some 3 ppm below the range of ca. 7–9 ppm found for all other PHMes*-bridged complexes reported in this work (see the Experimental Section). Indeed, the syn conformation involves not only a close proximity between Ph and Cp rings but also a close approach between the Ph ring and the P-bound H atom, which ends up close to the perpendicular to the ring plane. This is an ideal position to experience the shielding effect (through space) derived from the magnetic anisotropy of the Ph ring.^37^ Such a shielding effect should be absent in the anti conformer since the S-bound phenyl ring now points away from the intermetallic region and hence from the P-bound H atom. In agreement with this, the chemical shift of this atom in *anti***-6** is 7.16 ppm, a figure comparable to those of all other PHMes*-bridged complexes described in this work. The different P–H couplings of the P-bound hydrogens enabled us to assign the corresponding ^31^P resonances, which appear at −8.1 ppm (*syn***-6**) and −33.4 ppm (*anti***-6**), close to that of the hydride complex **2a** and some 200 ppm above the resonance of the parent hexacarbonyl complex **5**, in agreement with the presence of a metal–metal bond in these pentacarbonyl complexes.

### Reactions of Compounds **1** with Silanes and Stannanes

These heavier analogues of hydrocarbons have in common the presence of H–E bonds with negatively polarized H atoms and the absence of lone electron pairs at the E atom. Silanes H_*x*_SiPh_4–*x*_ (*x* = 1–3) did not react with compounds **1a,b** at room temperature but did react upon heating to give the hydrides **2a,b** as the only identified new products, along with other species, depending on the particular silane used. The selectivity of the reaction was very good for H_3_SiPh, which was also the one reacting faster at 333 K, whereas the reaction with HSiPh_3_ required prolonged heating at 363 K and produced only very small amounts of these hydrides, with extensive decomposition being observed. Not surprisingly, silane H_2_SiPh_2_ behaved in an intermediate way, yielding the hydride complexes **2a,b** in only modest yields (ca. 20%). In light of the results of reactions of compounds **1a,b** with HSnPh_3_ to be discussed below, it is likely that the eventual hydrogenation observed in the reactions of **1a,b** with silanes is initiated with a H–Si bond cleavage step with specific formation of P–H and Si–M bonds to give intermediate silyl complexes of type [MoReCp(μ-PHMes*)(CO)_6_(H_*x*–1_SiPh_4–*x*_)], the latter decomposing by extrusion of SiHPh or SiPh_2_ fragments to give the hydrides **2a,b**. The latter step would not render an M–H bond in the reaction with HSiPh_3_, thus explaining the very small amounts of hydrides formed in this case.

**Scheme 5:**
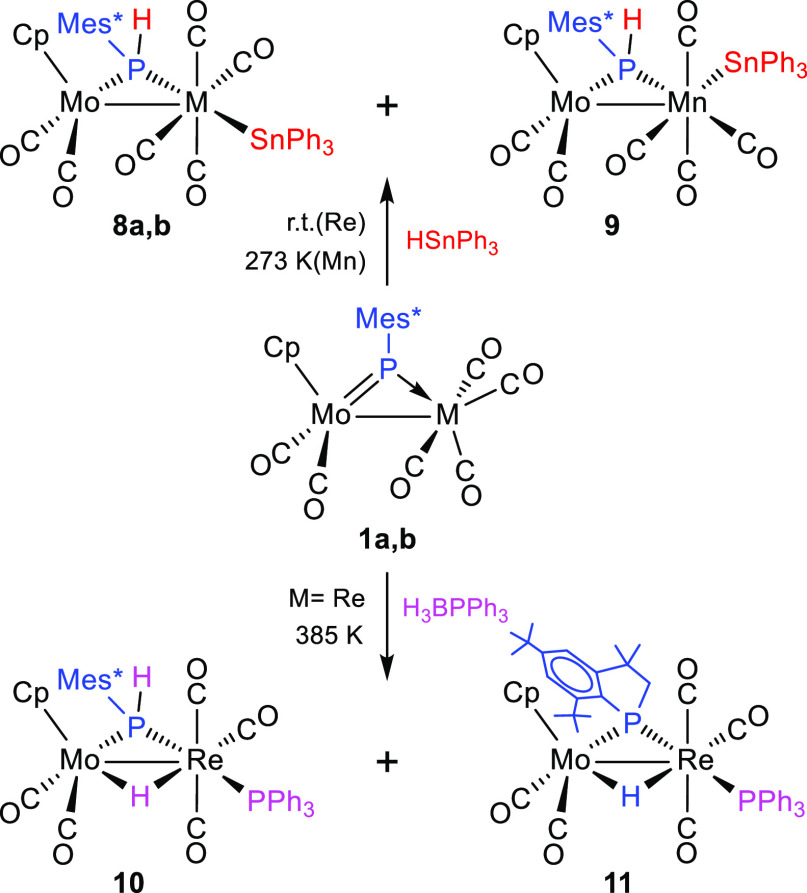
Reactions of Compounds **1** with HSnPh_3_ and H_3_BPPh_3_

Stannane HSnPh_3_ proved to be far more reactive than its silicon analogue and, in fact, reacted with compounds **1a,b** at room temperature (**1a**) or even 273 K (**1b**) in toluene solution. In both cases, the reaction proceeded selectively with cleavage of the Sn–H bond and specific formation of P–H and M–Sn bonds (Scheme 5). The reaction with the rhenium complex **1a** selectively yielded *trans*-[MoReCp(μ-PHMes*)(CO)_6_(SnPh_3_)] (**8a**), with a transoid arrangement of the stannyl group relative to the bridging phosphanide ligand. In contrast, the manganese complex **1b** yielded a mixture of the analogous complex *trans*-[MoMnCp(μ-PHMes*)(CO)_6_(SnPh_3_)] (**8b**) and its isomer *cis*-[MoMnCp(μ-PHMes*)(CO)_6_(SnPh_3_)] (**9**), the latter displaying a cisoid arrangement of the stannyl group relative to the PHMes* ligand. Isomers **8b** and **9** could be separated by fractional crystallization (see the Experimental Section) but would interconvert slowly upon dissolution in toluene or dichloromethane to reach in both cases an equilibrium ratio **8b**/**9** of ca. 2:1 at room temperature in ca. 30 min, irrespective of whether starting from **8b** or **9**.

The most energetic C–O stretch present in the IR spectrum of compounds **8a,b** appears at ca. 2065 cm^–1^ and has a very weak intensity in both cases, which denotes the presence of a transoid M(CO)_4_ fragment in the molecule with a local *D*_4*h*_ symmetry,^18^ only possible if the stannyl group is placed trans to the PHMes* ligand. Although we were not able to grow crystals of enough quality for a conventional single crystal X-ray diffraction analysis of these compounds, a study of a crystal of the manganese compound **8b** confirmed the proposed stereochemistry, even if the precision of the data was rather poor. In particular, the intermetallic distances were found to be 3.051(5) (Mo–Mn) and 2.658(5) Å (Mn–Sn), with the relevant angles being 153.1(1) (Mo–Mn–Sn) and 156.4(2)° (P–Mn–Sn). We note that the position of the stannyl group in this molecule is similar to the one determined for the related MoRe complex [MoReCp(μ-PCy_2_)(CO)_5_(NCMe)(SnPh_3_)] [Mo–Re–Sn = 143.67(2) and P–Re–Sn = 166.74(4)°].^26^ This transoid arrangement between P and Sn atoms is also supported by the observation in solution of a relatively large two-bond P–Sn coupling of 121 Hz in the ^31^P spectrum of the rhenium complex **8a** (cf. 183 Hz in the mentioned PCy_2_-bridged analogue).

**Figure 3:**
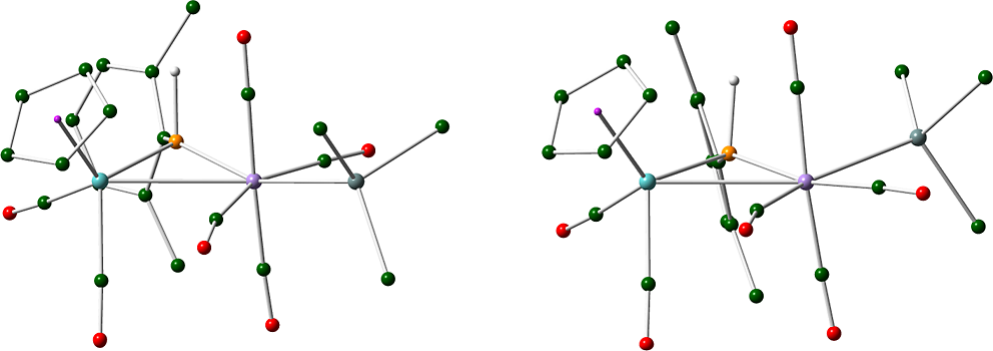
M06L-DFT-computed structures of isomers **8b** (left) and **9** (right), with ^*t*^Bu and Ph groups (except their C^1^ atoms) and most H atoms omitted for clarity.

In contrast to that in **8b**, the most energetic C–O stretch present in the IR spectrum of isomer **9** (2054 cm^–1^) is of high intensity, which denotes the presence of a disphenoidal Mn(CO)_4_ oscillator in the molecule with a local C_2v_ symmetry, this requiring the stannyl group to be positioned cis to the P atom. Incidentally, this would also imply that the P atom would now face a carbonyl ligand trans to it instead of the stannyl group. This strong increase in the electron-withdrawing properties of the ligand trans to phosphorus is expected to result in a significant shielding of the ^31^P resonance in **9** (relative to **8b**), which is in agreement with the observed chemical shift of **9** (δ_P_ 89.5 ppm), some 45 ppm below that of **8b**.^37^ Nevetheless, because of the low symmetry of the molecule, there are four different positions for the stannyl group fulfilling the condition of being positioned cis to P, which cannot be easily distinguished from each other spectroscopically. Then, we resorted to DFT calculations (see the Experimental Section) in search for the cisoid isomer having about the same energy as that of the major isomer **8b** to find that this is the one having the stannyl group placed in the MoPMn plane and pointing away from the dimetal center (Figure 3 and Table 4). The computed difference in the Gibbs free energies of **8b** and **9** in dichloromethane solution at 298 K is just 2.6 kJ/mol in favor of **8b**, which is consistent with the coexistence of these isomers in solution and with the observed equilibrium ratio. Interestingly, the optimized structure of **9** reveals the presence of a weak semibridging interaction of a carbonyl ligand (the one trans to Sn) with the Mo atom (Mo···C = 2.635 Å and Mn–C–O = 159.0°).^38^ This is consistent with the presence in the IR spectrum of **9** of a C–O stretch at a relatively low frequency of 1840 cm^–1^.

**Table 4:** Selected M06L-DFT-Computed Bond Lengths (Å) and Angles (deg) for Isomers **8b** and **9**

parameter	**8b**	**9**	parameter	**8b**	**9**
Mo–Mn	3.036	3.047	Mo–P–Mn	82.3	76.8
Mn–Sn	2.693	2.755	Mo–Mn–Sn	135.3	141.0
Mo–P	2.424	2.470	P–Mn–Sn	160.2	89.6
Re–P	2.333	2.437			

### Reactions of Compounds **1** with Boranes

Compounds **1a,b** failed to react with the borane adduct BH_3_·THF in toluene solution at room temperature. Upon increasing the temperature, only the manganese complex underwent some transformation to give the hydrogenation derivative **2b** in low yield, along with other minor uncharacterized species. Presumably, decomposition of the reagent prevails when these reactions are performed above room temperature.

The reaction of the rhenium complex **1a** with the more robust adduct BH_3_·PPh_3_ did not proceed at room temperature either, but in refluxing toluene solution it gave a mixture of the hydride complexes [MoReCp(μ-H)(μ-PHMes*)(CO)_5_(PPh_3_)] (**10**) and [MoReCp(μ-H){μ-P(CH_2_CMe_2_)C_6_H_2_^*t*^Bu_2_}(CO)_5_(PPh_3_)] (**11**) in comparable amounts (ca. 25% yield each) as major products (Scheme 5). The formation of **10** requires hydrogenation of **1a** (to give **2a**) and replacement of a carbonyl ligand with PPh_3_, and indeed a separate experiment indicated that the hydride **2a** reacts with PPh_3_ in refluxing toluene solution to yield selectively **10** in ca. 50 min (see the Experimental Section). On the other hand, the formation of **11** requires the intramolecular cleavage of a C–H bond in an *ortho*-^t^Bu group as well as CO/PPh_3_ substitution. Previous studies have shown that the first process takes place slowly when refluxing toluene solutions of **1a** to yield its hydride-bridged isomer [MoReCp(μ-H){μ-P(CH_2_CMe_2_)C_6_H_2_^*t*^Bu_2_}(CO)_6_].^11^ Not surprisingly, a separate experiment now indicated that the latter compound reacts slowly with PPh_3_ at 363 K to give **11** in a selective way. In all, these experiments suggest that the formation of compounds **10** and **11** does not stem from a genuine reaction with the reagent but rather with the likely products of its thermal degradation, H_2_ and PPh_3_, among others. The formation of **10** likely follows from the sequence hydrogenation/CO substitution, whereas that of **11** would most certainly follow from the sequence C–H cleavage/CO substitution.

Spectroscopic data for compounds **10** and **11** are comparable to those of the corresponding hexacarbonyl precursors noted above and deserve no detailed comment except for the features associated with the presence of the PPh_3_ ligand at the Re atom. Its transoid positioning relative to the bridging phosphanide ligand in each case can be first inferred from the high P–P coupling of 85 Hz observed in both cases, comparable to that measured for the phosphinidene complex **7a** (92 Hz). In addition, no large P–C couplings of ca. 32 Hz (corresponding to CO ligands trans to P) are observed among the Re-bound carbonyls, in contrast to the parent complexes. Moreover, their IR spectra display their most energetic band at ca. 2037 cm^–1^ with a weak intensity, thus denoting the meridional or T-shaped arrangement of the carbonyl ligands around the Re atom that the position of PPh_3_ forces in these molecules. Finally, we note that the resonance for the bridging hydride in these complexes, which appear at ca. – 12.5 ppm, display similar couplings of 20 and 12 Hz to the inequivalent P atoms of these molecules, in agreement with the cisoid positioning of the hydride ligand with respect to both P donor ligands.

### Mechanism of the Reaction of Compounds **1** with H_2_

**Figure 4:**
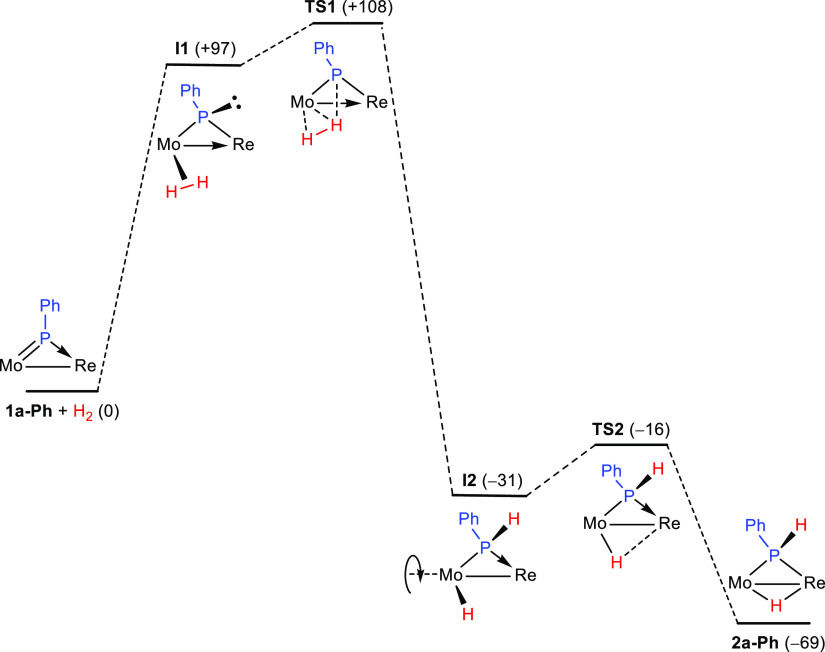
M06L-DFT-computed kinetic profile for the hydrogenation of the model compound **1a-Ph**, with Gibbs free energies relative to reactants (in kJ/mol) indicated between brackets.

As noted above, the mild conditions under which compounds **1a,b** react with hydrogen are very unusual features of the reactivity of these phosphinidene complexes, only paralleled by the diiron complex depicted in Scheme 2. Thus, it was of interest to gain further insight into this unusual hydrogenation reaction by analyzing its possible reaction pathway, which we have performed on the model rhenium complex [MoReCp(μ-PPh)(CO)_6_] (**1a-Ph**) by using DFT methods. Sterenberg and co-workers have reported similar calculations on the addition of SiH_4_ or H_2_ to the P atom of the model phosphinidene complex [FeCp(PNMe_2_)(CO)_2_]^+^. These unveiled the relevance of the orientation of the reagent in approaching the P atom to better facilitate the electrostatic and orbital interactions eventually needed to cleave the pertinent H–Si (or H–H) bond.^39^ Our reaction is more complex as it might involve all P, Mo, and Re atoms in one way or another since one H atom of the H_2_ molecule ends up bridging Mo and Re atoms, while the other one binds the P atom. Our calculations suggest that the approach of the H_2_ molecule to **1a-Ph** is an endergonic process taking place at the Mo atom with a side-on orientation and from a direction roughly perpendicular to the Mo–P–Re plane to give a true η^2^-dihydrogen complex^1^ (intermediate **I1**, 97 kJ/mol above reactants, see Figure 4 and the Supporting Information). This would follow from the interaction between the σ-bonding orbital of H_2_ and the LUMO of **1a-Ph** (π*-antibonding component of the Mo=P double bond; see the Supporting Information). As a result of it, the H–H bond is moderately elongated (H–H = 0.807 Å), and the Mo–P bond order is reduced to 1 and strongly elongated (Mo–P = 2.645 Å, vs 2.294 Å in **1a-Ph**), while the two electrons added to the molecule cause a strong pyramidalization at the P atom to keep the electron count of the complex constant (34 electrons), with retention of the intermetallic bond (Mo–Re = 3.083 Å). From here, the system evolves for full H–H bond cleavage through the transition state **TS1** (+108 kJ/mol), a sort of elongated η^2^-dihydrogen complex (Mo–H = 1.843 Å and H–H = 1.022 Å),^1^ with one of the H atoms initiating its binding to the P atom (P···H = 1.756 Å). This is possible since the lone pair developed at the P atom points in a direction close to the H–H bond axis, thus enabling its interaction with the σ*-antibonding orbital of the H_2_ molecule. Such an interaction promotes the progressive stretching and eventual cleavage of the H–H bond, while the P–H and Mo–H distances become shorter to yield intermediate **I2** (−31 kJ/mol), which bears conventional P–H (1.409 Å) and Mo–H (1.738 Å) bonds. We note that the formation of **I2** is itself an exergonic process and completes the addition of the dihydrogen molecule over the Mo=P double bond of **1a-Ph**. However, intermediate **I2** then rearranges easily by rotation of the MoCp(CO)_2_H fragment through low-energy transition state **TS2** (−16 kJ/mol), whereby the terminal Mo-bound hydride progressively approaches the Re atom, to eventually yield the hydride-bridged isomer **2a-Ph**, significantly more stable (−69 kJ/mol). The Gibbs free energy of 108 kJ/mol for **TS1** defines the overall kinetic barrier of the process, consistent with reactions taking place slowly at room temperature, as experimentally observed for the actual PHMes*-bridged complexes **1a,b**.

## Conclusions

The phosphinidene complexes [MoMCp(μ-PMes*)(CO)_6_] (M = Re and Mn) react with hydrogen under mild conditions to selectively give the hydride- and phosphanide-bridged complexes [MoMCp(μ-H)(μ-PHMes*)(CO)_6_]. DFT calculations on the PPh-bridged model of the rhenium complex suggest that these reactions might be initiated with coordination of the H_2_ molecule at the Mo atom to give a η^2^-H_2_ intermediate complex, which then evolves with full H–H bond cleavage over the Mo–P bond to eventually yield the hydride-bridged complexes actually isolated. This would be providing the first example of a genuine dihydrogen addition taking place at a phosphinidene-bridged complex. E–H bond cleavage also takes place in reactions with thiols, silanes, and stannanes, with specific formation of P–H bonds irrespective of the polarity of H in the reagent, to give either 36-electron complexes as [MoMCp(μ-PHMes*)(μ-SPh)(CO)_6_] or 34-electron complexes as [MoReCp(μ-PHMes*)(CO)_6_(SnPh_3_)]. In contrast, no P–H bond activation was observed in the reactions with phosphine PHCy_2_, which instead yielded the CO substitution products at the M atom [MoMCp(μ-PMes*)(CO)_5_(PHCy_2_)], with the transoid positioning of the phosphine relative to the PMes* ligand perhaps preventing any further P–H bond cleavage processes. One electron reduction of the rhenium complex also provides an alternative route to hydrogenation of these reactive phosphinidene complexes as spontaneous H atom abstraction (likely from trace water) takes place at the P atom of the putative radical intermediate [MoReCp(μ-PMes*)(CO)_6_]^−^ first formed to give the phosphanide-bridged anion [MoReCp(μ-PHMes*)(CO)_6_]^−^, which is easily transformed into the corresponding neutral hydride-bridged complex upon protonation.

## Experimental Section

### General Procedures and Starting Materials

General experimental procedures, as well as the preparation of compounds [MoReCp(μ-PMes*)(CO)_6_] (**1a**), [MoMnCp(μ-PMes*)(CO)_6_] (**1b**), and [MoReCp(μ-H){μ-P(CH_2_CMe_2_)C_6_H_2_^*t*^Bu_2_}(CO)_6_], were carried out as described previously (Cp = η^5^-C_5_H_5_; Mes* = 2,4,6-C_6_H_2_^*t*^Bu_3_).^11a,13^ Complex [AuCl{P(*p*-tol)_3_}] was prepared by literature methods,^40^ and other reagents were obtained from commercial suppliers and used as received.

### Preparation of [MoReCp(μ-H)(μ-PHMes*)(CO)_6_] (**2a**)

Method A: Compound **1a** (0.020 g, 0.025 mmol) was dissolved in toluene (6 mL) in a Schlenk tube equipped with a Young’s valve, and the solution was set under a hydrogen atmosphere at ca. 77 K. After closing the valve, the solution was stirred at 363 K for 3 h to give an orange solution. The solvent was then removed under vacuum, the residue was extracted with dichloromethane/petroleum ether (1/4), and the extracts were chromatographed on alumina at 258 K. Elution with the same solvent mixture gave a yellow orange fraction yielding, after removal of solvents, compound **2a** as a yellow solid (0.015 g, 76%). Method B: Neat SiH_3_Ph (4 μL, 0.032 mol) was added to a toluene solution (6 mL) of compound **1a** (0.020 g, 0.025 mmol), and the mixture was stirred at 333 K for 1 h to give an orange solution. Workup as before yielded 0.013 g (66%) of compound **2a**. Method C: Excess [NH_4_]PF_6_ (0.010 g, 0.062 mmol) was added to a solution of compound **Na-3**, prepared in situ from compound **1a** (0.020 g, 0.025 mmol), as described below, and the mixture was stirred at room temperature for 5 min to give a yellow solution. Workup as before yielded 0.014 g (71%) of compound **2a**. Anal. Calcd for C_29_H_36_MoO_6_PRe: C, 43.88; H, 4.57. Found: C, 44.16; H, 4.34. ^1^H NMR (300.13 MHz, CD_2_Cl_2_): δ 7.63 (d, *J*_HP_ = 343, 1H, PH), 7.38 (d, *J*_HP_ = 4, 2H, C_6_H_2_), 5.17 (s, 5H, Cp), 1.52 (s, br, 18H, *o*-^*t*^Bu), 1.31 (s, 9H, *p*-^*t*^Bu), and −13.32 (d, *J*_HP_ = 20, μ-H). ^13^C{^1^H} NMR (100.63 MHz, CD_2_Cl_2_): δ 239.8 (d, *J*_CP_ = 25, MoCO), 238.8 (s, MoCO), 187.1 (d, *J*_CP_ = 32, ReCO), 185.5 (d, *J*_CP_ = 7, ReCO), 185.3 (s, ReCO), 183.5 (d, *J*_CP_ = 6, ReCO), 157.6 [d, *J*_CP_ = 4, C^2^(C_6_H_2_)], 150.5 [d, *J*_CP_ = 4, C^4^(C_6_H_2_)], 129.2 [d, *J*_CP_ = 27, C^1^(C_6_H_2_)], 123.4 [d, *J*_CP_ = 11, C^3^(C_6_H_2_)], 91.5 (s, Cp), 39.3 [s, C^1^(*o*-^*t*^Bu)], 35.1 [s, C^1^(*p*-^*t*^Bu)], 34.1 [s, br, C^2^(*o*-^*t*^Bu)], 31.2 [s, C^2^(*p*-^*t*^Bu)].

### Preparation of [MoMnCp(μ-H)(μ-PHMes*)(CO)_6_] (**2b**)

Compound **1b** (0.020 g, 0.030 mmol) was dissolved in toluene (6 mL) in a Schlenk tube equipped with a Young’s valve, and the solution was set under a hydrogen atmosphere at 77 K. After closing the valve, the solution was stirred at room temperature for 3 days to give an orange solution. Workup as described for **2a** yielded compound **2b** as an orange solid (0.015 g, 76%). Anal. Calcd for C_29_H_36_MoMnO_6_P: C, 52.58; H, 5.48. Found: C, 52.47; H, 4.96. ^1^H NMR (300.13 MHz, CD_2_Cl_2_): δ 7.51 (d, *J*_HP_ = 336, 1H, PH), 7.38 (d, *J*_HP_ = 3, 2H, C_6_H_2_), 5.16 (s, 5H, Cp), 1.50 (s, 18H, *o*-^*t*^Bu), 1.31 (s, 9H, *p*-^*t*^Bu), −13.94 (d, *J*_HP_ = 33, μ-H). ^13^C{^1^H} NMR (100.63 MHz, CD_2_Cl_2_): δ 241.2 (d, *J*_CP_ = 23, MoCO), 236.6 (s, MoCO), 218.4, 217.8, 212.6, 209.9 (4s, br, MnCO), 158.0 [d, *J*_CP_ = 4, C^2^(C_6_H_2_)], 150.8 [d, *J*_CP_ = 4, C^4^(C_6_H_2_)], 132.1 [d, *J*_CP_ = 24, C^1^(C_6_H_2_)], 123.3 [d, *J*_CP_ = 10, C^3^(C_6_H_2_)], 91.7 (s, Cp), 39.3 [s, C^1^(*o*-^*t*^Bu)], 35.1 [s, C^1^(*p*-^*t*^Bu)], 34.1 [s, C^2^(*o*-^*t*^Bu)], 31.2 [s, C^2^(*p*-^*t*^Bu)].

### Preparation of Tetrahydrofuran Solutions of Na[MoReCp(μ-PHMes*)(CO)_6_] (**Na-3**)

Excess Na(Hg) (ca. 0.5 mL of a 0.5% amalgam, ca. 1.5 mmol) was added to a tetrahydrofuran solution (6 mL) of compound **1a** (0.020 g, 0.025 mmol), and the mixture was stirred at 273 K for 30 min to give a green solution containing compound **Na-3** as the major product, ready for use in further reactions (assumed 100% yield).

### Preparation of [MoReAuCp(μ-PHMes*)(CO)_6_{P(*p*-tol)_3_}] (**4**)

Solid [AuCl{P(*p*-tol)_3_}] (0.020 g, 0.037 mmol) was added to a filtered solution of compound **Na-3** (ca. 0.025 mmol) prepared as described before, and the mixture was stirred at 273 K for 30 min to give a brown solution. Workup as described for **2a** [extraction and elution with toluene/petroleum ether (1/2)] yielded compound **4** as a yellow microcrystalline solid (0.016 g, 49%). X-ray quality crystals of **4** were grown by the slow diffusion of a layer of petroleum ether into a concentrated toluene solution of the complex at 253 K. Anal. Calcd for C_50_H_56_AuMoO_6_P_2_Re: C, 46.41; H, 4.36. Found: C, 46.15; H, 4.19. ^1^H NMR (300.13 MHz, C_6_D_6_): δ 8.03 (d, *J*_HP_ = 341, 1H, PH), 7.76 [dd, *J*_HP_ = 12, *J*_HH_ = 8, 6H, H^2^(C_6_H_4_)], 7.63, 7.55 (2s, br, 2 × 1H, C_6_H_2_), 6.98 [d, *J*_HH_ = 8, 6H, H^3^(C_6_H_4_)], 4.75 (s, 5H, Cp), 1.97 (s, 9H, Me), 1.76, 1.53 (2s, br, 2 × 9H, *o*-^*t*^Bu), 1.38 (s, 9H, *p*-^*t*^Bu). ^13^C{^1^H} NMR (100.63 MHz, C_6_D_6_): δ 237.7 (d, *J*_CP_ = 23, MoCO), 233.6 (d, *J*_CP_ = 4, MoCO), 202.9 (d, *J*_CP_ = 33, ReCO), 190.5 (s, br, 2ReCO), 187.7 (d, *J*_CP_ = 6, ReCO), 157.9 [s, br, C^1^(C_6_H_2_)], 150.4 [s, C^4^(C_6_H_2_)], 149.6, 149.5 [2s, C^2^(C_6_H_2_)], 141.3 [s, C^4^(C_6_H_4_)], 134.4 [d, *J*_CP_ = 15, C^2^(C_6_H_4_)], 130.8 [d, *J*_CP_ = 46, C^1^(C_6_H_4_)], 130.1 [d, *J*_CP_ = 11, C^3^(C_6_H_4_)], 123.0 [s, br, C^3^(C_6_H_2_)], 88.5 (s, Cp), 39.5 [s, C^1^(*o*-^*t*^Bu)], 34.9 [s, C^1^(*p*-^*t*^Bu)], 34.7, 34.1 [2s, br, C^2^(*o*-^*t*^Bu)], 31.4 [s, C^2^(*p*-^*t*^Bu)], 21.2 (s, Me).

### Reaction of Compound **1a** with HSPh

Neat thiophenol (6 μL, 0.059 mmol) was added to a toluene solution (6 mL) of compound **1a** (0.040 g, 0.051 mmol) in a Schlenk tube equipped with a Young’s valve. After closing the valve, the solution was stirred at 333 K for 3 d to give an orange solution. Workup was similar to that described for **2a**. Elution with dichloromethane/petroleum ether (1/20) gave orange and yellow fractions, yielding compounds [MoReCp(μ-PHMes*)(μ-SPh)(CO)_6_] (**5**) (0.010 g, 22%) and [MoReCp(μ-PHMes*)(μ-SPh)(CO)_5_] (**6**) (0.012 g, 27%) as orange and yellow solids, respectively, with the latter appearing as an equilibrium mixture of *syn* and *anti* isomers in solution. Another yellow fraction could be collected by elution with dichloromethane/petroleum ether (2/1), this one likely containing the dithiolate complex [MoReCp(μ-SPh)_2_(CO)_5_], not further investigated (see the text). X-ray quality crystals of **5** were grown by the slow diffusion of a layer of petroleum ether into a concentrated dichloromethane solution of the complex at 253 K. Data for compound **5**: Anal. Calcd for C_35_H_40_MoO_6_PReS: C, 46.61; H, 4.47; S, 3.56. Found: C, 46.35; H, 4.11; S, 3.37. ^1^H NMR (400.13 MHz, CD_2_Cl_2_, 253 K): δ 7.32–7.24 (m, 7H, C_6_H_2_ + Ph), 6.90 (d, *J*_HP_ = 304, PH), 5.12 (s, 5H, Cp), 1.90, 1.58, 1.25 (3s, 3 × 9H, ^*t*^Bu). ^13^C{^1^H} NMR (100.63 MHz, CD_2_Cl_2_, 253 K): δ 255.2 (s, MoCO), 249.3 (d, *J*_CP_ = 22, MoCO), 195.9 (s, ReCO), 192.0 (d, *J*_CP_ = 8, ReCO), 187.6 (s, ReCO), 187.2 (d, *J*_CP_ = 4, ReCO), 156.6, 156.5 [2s, C^2,6^(C_6_H_2_)], 155.5 [s, C^4^(C_6_H_2_)], 149.4 [s, C^1^(Ph)], 141.3 [d, *J*_CP_ = 17, C^1^(C_6_H_2_)], 132.4 [s, C^2^(Ph)], 129.0 [s, C^3^(Ph)], 126.1 [s, C^4^(Ph)], 122.7 [d, *J*_CP_ = 7, C^3,5^(C_6_H_2_)], 122.0 [d, *J*_CP_ = 10, C^5,3^(C_6_H_2_)], 95.6 (s, Cp), 40.2, 38.8 [2s, C^1^(^*t*^Bu)], 34.3 [s, C^2^(^*t*^Bu)], 34.1 [d, *J*_CP_ = 4, C^2^(^*t*^Bu)], 32.1 [s, C^1^(^*t*^Bu)], 31.2 [s, C^2^(^*t*^Bu)]. Data for compound **6**: Anal. Calcd for C_34_H_40_MoO_5_PReS: C, 46.73; H, 4.61; S, 3.67. Found: C, 46.40; H, 4.31; S, 3.45. ^1^H NMR (400.13 MHz, CD_2_Cl_2_): Isomer *syn*: δ 7.47–7.23 (m, 7H, C_6_H_2_ + Ph), 5.18 (s, 5H, Cp), 4.17 (d, *J*_HP_ = 352, PH), 1.57 (s, br, 18H, *o*-^*t*^Bu), 1.32 (s, br, 9H, *p*-^*t*^Bu). Isomer *anti*: δ 7.47–7.23 (m, 7H, C_6_H_2_ + Ph), 7.16 (d, *J*_HP_ = 368, PH), 5.18 (s, 5H, Cp), 1.57 (s, 18H, *o*-^*t*^Bu), 1.32 (s, br, 9H, *p*-^*t*^Bu). Ratio syn/anti = ca. 3:2.

### Preparation of [MoReCp(μ-PMes*)(CO)_5_(PHCy_2_)] (**7a**)

Neat PHCy_2_ (8 μL, 0.036 mol) was added to a toluene solution (6 mL) of compound **1a** (0.020 g, 0.025 mmol), and the mixture was stirred at room temperature for 1 h to give a purple solution. Workup as described for **2a** (extraction and elution with petroleum ether) yielded compound **7a** as a purple microcrystalline solid (0.018 g, 75%). Anal. Calcd for C_40_H_57_MoO_5_P_2_Re: C, 49.94; H, 5.97. Found: C, 49.78; H, 6.21. ^1^H NMR (CD_2_Cl_2_, 400.13 MHz): δ 7.43 (s, 2H, C_6_H_2_), 5.50 (d, *J*_HP_ = 339, 1H, PH), 4.97 (s, 5H, Cp), 2.45–1.72 (m, 22H, Cy), 1.49 (s, br, 18H, *o*-^*t*^Bu), 1.39 (s, 9H, *o*-^*t*^Bu). ^13^C{^1^H} NMR (100.63 MHz, CD_2_Cl_2_): δ 233.3 (s, MoCO), 200.6 (s, ReCO), 196.6 (t, *J*_CP_ = 9, 2ReCO), 153.9 [d, *J*_CP_ = 33, C^1^(C_6_H_2_)], 151.2 [s, C^4^(C_6_H_2_)], 150.6 [s, C^2^(C_6_H_2_)], 122.3 [d, *J*_CP_ = 5, C^3^(C_6_H_2_)], 93.2 (s, Cp), 39.0 [s, C^1^(*o*-^*t*^Bu)], 36.3 [d, *J*_CP_ = 29, C^1^(Cy)], 35.4 [s, C^1^(*p*-^*t*^Bu)], 33.3 [s, C^2^(*o*-^*t*^Bu)], 32.8 [s, C^2^(Cy)], 31.3 [s, C^2^(*p*-^*t*^Bu)], 30.4 [s, C^2^(Cy)], 27.5 [d, *J*_CP_ = 11, C^3^(Cy)], 27.4 [d, *J*_CP_ = 11, C^3^(Cy)], 26.4 [s, C^4^(Cy)].

### Preparation of [MoMnCp(μ-PMes*)(CO)_5_(PHCy_2_)] (**7b**)

Neat PHCy_2_ (10 μL, 0.046 mol) was added to a toluene solution (6 mL) of compound **1b** (0.020 g, 0.030 mmol), and the mixture was stirred at room temperature for 1.5 h to give a purple solution. Workup as described for **2a** [extraction and elution with dichloromethane/petroleum ether (1/20)] yielded compound **7b** as a purple microcrystalline solid (0.018 g, 72%). Anal. Calcd for C_40_H_57_MoMnO_5_P_2_: C, 57.83; H, 6.92. Found: C, 57.69; H, 7.32. ^1^H NMR (400.13 MHz, CD_2_Cl_2_): δ 7.42 (s, 2H, C_6_H_2_), 5.15 (d, *J*_HP_ = 325, 1H, PH), 4.94 (s, 5H, Cp), 2.50–1.74 (m, 22H, Cy), 1.53 (s, 18H, *o*-^*t*^Bu), 1.39 (s, 9H, *o*-^*t*^Bu). ^13^C{^1^H} NMR (100.63 MHz, CD_2_Cl_2_, 233 K): δ 233.5 (s, MoCO), 231.4 (s, MnCO), 219.2 (s, br, 2MnCO), 155.8 [d, *J*_CP_ = 31, C^1^(C_6_H_2_)], 150.7 [s, C^4^(C_6_H_2_)], 149.3 [s, C^2^(C_6_H_2_)], 121.3 [s, C^3^(C_6_H_2_)], 93.3 (s, Cp), 38.2 [s, C^1^(*o*-^*t*^Bu)], 34.9 [s, C^1^(*p*-^*t*^Bu)], 34.7 [s, C^1^(Cy)], 32.4 [s, C^2^(*o*-^*t*^Bu)], 32.3 [s, C^2^(Cy)], 30.7 [s, C^2^(*p*-^*t*^Bu)], 29.4 [s, C^2^(Cy)], 27.2 [d, *J*_CP_ = 11, C^3^(Cy)], 27.1 [d, *J*_CP_ = 11, C^3^(Cy)], 25.7 [s, C^4^(Cy)].

### Preparation of *trans*-[MoReCp(μ-PHMes*)(CO)_6_(SnPh_3_)] (**8a**)

Compound **1a** (0.030 g, 0.038 mmol) and HSnPh_3_ (0.013 g, 0.037 mol) were dissolved in toluene (8 mL), and the mixture was stirred at room temperature for 1.5 h to give an orange solution. Workup as described for **2a** [extraction and elution with toluene/petroleum ether (1/8)] yielded compound **8a** as a red microcrystalline solid (0.025 g, 58%). Anal. Calcd for C_47_H_50_MoO_6_PReSn: C, 49.40; H, 4.41. Found: C, 49.23; H, 4.32. ^1^H NMR (400.13 MHz, toluene-*d*_8_): δ 8.84 (d, *J*_HP_ = 348, 1H, PH), 7.79 [d, *J*_HH_ = 6, *J*_117SnH∼_–*J*_119SnH_ = 46, 6H, H^2^(Ph)], 7.39 (s, br, 2H, C_6_H_2_), 7.21–7.10 (m, 9H, Ph), 4.75 (s, 5H, Cp), 1.59, 1.40 (2s, br, 2 × 9H, *o*-^*t*^Bu), 1.21 (s, 9H, *p*-^*t*^Bu). ^13^C{^1^H} NMR (100.63 MHz, toluene-*d*_8_): δ 242.7 (d, *J*_CP_ = 23, MoCO), 233.5 (s, MoCO), 194.1 (s, br, *J*_117SnH_∼*J*_119SnH_ = 44, 4ReCO), 159.4 [s, C^4^(C_6_H_2_)], 157.7 [d, *J*_CP_ = 6, C^1^(C_6_H_2_)], 151.2, 151.1 [2s, C^2,6^(C_6_H_2_)], 142.0 [s, *J*_119SnH_ = 399, *J*_117SnH_ = 381, C^1^(Ph)], 129.3 [s, C^2^(Ph)], 128.5 [s, C^3^(Ph)], 125.7 [s, C^4^(Ph)], 123.5, 123.4 [2s, C^3,5^(C_6_H_2_)], 92.6 (s, Cp), 40.0, 39.7, 34.8 [3s, C^1^(^*t*^Bu)], 34.3, 34.1, 31.0 [3s, C^2^(^*t*^Bu)].

### Reaction of Compound **1b** with HSnPh_3_

Compound **1b** (0.020 g, 0.030 mmol) and HSnPh_3_ (0.011 g, 0.031 mol) were dissolved in toluene (8 mL) at 273 K, and the mixture was stirred at this temperature for 3 h to give an orange solution containing the isomers *trans*-[MoMnCp(μ-PHMes*)(CO)_6_(SnPh_3_)] (**8b**) and *cis*-[MoMnCp(μ-PHMes*)(CO)_6_(SnPh_3_)] (**9**) in a ratio of ca. 2:1. After removal of the solvent, the residue was extracted with dichloromethane and filtered. Removal of the solvent from the filtrate and washing of the residue with petroleum ether (2 × 5 mL) gave a brownish powder containing both isomers (0.020 g, 66%). Crystallization of the mixture of isomers by the slow diffusion of a layer of petroleum ether into a concentrated dichloromethane solution of the crude product at 253 K yielded red-brown crystals of **8b** and orange crystals of **9**, which could be separated manually from each other by using a microscope. Data for **8b**: Anal. Calcd for C_47_H_50_MnMoO_6_PSn: C, 55.81; H, 4.98. Found: C, 55.63; H, 4.72. ^1^H NMR (400.13 MHz, CD_2_Cl_2_): δ 8.56 (d, *J*_HP_ = 340, 1H, PH), 7.60–7.16 (m, 17H, C_6_H_2_ + Ph), 5.28 (s, 5H, Cp), 1.71, 1.43, 1.28 (3s, 3 × 9H, ^*t*^Bu). Data for **9**: Anal. Calcd for C_47_H_50_MnMoO_6_PSn: C, 55.81; H, 4.98. Found: C, 55.55; H, 4.60. ^1^H NMR (400.13 MHz, CD_2_Cl_2_): δ 7.43 (d, *J*_HP_ = 344, 1H, PH), 7.78–7.75 (m, 5H, Ph), 7.39, 7.38 (2s, 2 × 1H, C_6_H_2_), 7.16–7.12 (m, 10H, Ph), 4.74 (s, 5H, Cp), 1.42, 1.40, 1.20 (3s, 3 × 9H, ^*t*^Bu).

### Reaction of Compound **1a** with H_3_BPPh_3_

Compound **1a** (0.040 g, 0.051 mmol) and H_3_BPPh_3_ (0.030 g, 0.109 mol) were dissolved in toluene (8 mL), and the mixture was refluxed for 4 h to give an orange solution. Workup as described for **2a** (extraction and elution with dichloromethane/petroleum ether 1/12) gave first a minor brown fraction of unreacted **1a** and then orange and yellow fractions, yielding, respectively, yellow orange [MoReCp(μ-H)(μ-PHMes*)(CO)_5_(PPh_3_)] (**10**) (0.013 g, 25%) and [MoReCp(μ-H){μ-P(CH_2_CMe_2_)C_6_H_2_^*t*^Bu_2_}(CO)_5_(PPh_3_)] (**11**). The latter was invariably contaminated with significant amounts of the residual borane-phosphine adduct. Specific methods of preparation of compounds **10** and **11** are given below.

### Preparation of [MoReCp(μ-H)(μ-PHMes*)(CO)_5_(PPh_3_)] (**10**)

Compound **2a** (0.020 g, 0.025 mmol) and PPh_3_ (0.008 g, 0.030 mmol) were dissolved in toluene (6 mL), and the mixture was refluxed for 50 min to give an orange solution. Workup as described for **2a** [extraction and elution with dichloromethane/petroleum ether (1/6)] yielded compound **10** as a yellow microcrystalline solid (0.015 g, 58%). Anal. Calcd for C_47_H_53_Cl_2_MoO_5_P_2_Re (**10**·CH_2_Cl_2_): C, 50.72; H, 4.80. Found: C, 51.13; H, 5.46. ^1^H NMR (400.13 MHz, CD_2_Cl_2_): δ 7.82 (d, *J*_HP_ = 345, 1H, PH), 7.68–7.63 (m, 5H, Ph), 7.49–7.47 (m, 10H, Ph), 7.35 (s, br, 2H, C_6_H_2_), 4.65 (s, 5H, Cp), 1.51 (s, br, 18H, *o*-^*t*^Bu), 1.31 (s, 9H, *p*-^*t*^Bu), −12.78 (dd, *J*_HP_ = 20, 13, 1H, μ-H). ^13^C{^1^H} NMR (100.63 MHz, CD_2_Cl_2_): δ 241.6 (s, MoCO), 241.1 (d, *J*_CP_ = 23, MoCO), 193.4 (s, br, ReCO), 192.4 (dd, *J*_CP_ = 9, 3, ReCO), 190.9 (t, br, *J*_CP_ = 9, ReCO), 157.7 [d, *J*_CP_ = 4, C^2^(C_6_H_2_)], 149.6 [s, C^4^(C_6_H_2_)], 136.6 [d, *J*_CP_ = 46, C^1^(Ph)], 133.8 [d, *J*_CP_ = 11, C^2^(Ph)], 132.0 [d, *J*_CP_ = 23, C^1^(C_6_H_2_)], 130.5 [s, C^4^(Ph)], 128.9 [d, *J*_CP_ = 9, C^3^(Ph)], 122.9 [d, *J*_CP_ = 8, C^3^(C_6_H_2_)], 90.9 (s, Cp), 39.4 [s, C^1^(*o*-^*t*^Bu)], 35.1 [s, C^1^(*p*-^*t*^Bu)], 34.1 [s, br, C^2^(*o*-^*t*^Bu)], 31.2 [s, C^2^(*p*-^*t*^Bu)].

### Preparation of [MoReCp(μ-H){μ-P(CH_2_CMe_2_)C_6_H_2_^*t*^Bu_2_}(CO)_5_(PPh_3_)] (**11**)

Compound [MoReCp(μ-H){μ-P(CH_2_CMe_2_)C_6_H_2_^*t*^Bu_2_}(CO)_6_] (0.030 g, 0.038 mmol) and PPh_3_ (0.010 g, 0.038 mol) were dissolved in toluene (6 mL), and the mixture was stirred at 363 K for 20 h to give a yellow solution. Workup as described for **2a** [extraction and elution with dichloromethane/petroleum ether (1/8)] gave a minor fraction of the unreacted starting complex. Elution with dichloromethane/petroleum ether (1/3) gave a major yellow fraction, yielding, after removal of solvents, compound **11** as a yellow microcrystalline solid (0.025 g, 64%). Anal. Calcd for C_46_H_49_MoO_5_P_2_Re: C, 53.85; H, 4.81. Found: C, 54.13; H, 4.82. ^1^H NMR (400.13 MHz, CD_2_Cl_2_): δ 7.70 (m, 6H, Ph), 7.48 (m, 9H, Ph), 7.37 (dd, *J*_HP_ = 5, *J*_HH_ = 2, 1H, C_6_H_2_), 7.23 (s, br, 1H, C_6_H_2_), 4.76 (s, 5H, Cp), 3.32 (t, *J*_HH_ = *J*_HP_ = 13, 1H, CH_2_), 2.05 (dd, *J*_HH_ = 13, *J*_HP_ = 6, 1H, CH_2_), 1.48, 1.43 (2s, 2 × 3H, CMe), 1.36, 1.29 (2s, 2 × 9H, ^*t*^Bu), −12.41 (dd, *J*_HP_ = 20, 12, 1H, μ-H). ^13^C{^1^H} NMR (100.63 MHz, CD_2_Cl_2_): δ 242.2 (d, *J*_CP_ = 25, MoCO), 240.3 (s, MoCO), 194.0 (s, br, ReCO), 192.0 (dd, *J*_CP_ = 9, 3, ReCO), 191.5 (dd, *J*_CP_ = 9, 7, ReCO), 158.4 [d, *J*_CP_ = 15, C^2,6^(C_6_H_2_)], 154.7 [d, *J*_CP_ = 6, C^6,2^(C_6_H_2_)], 152.5 [d, *J*_CP_ = 3, C^4^(C_6_H_2_)], 136.2 [d, *J*_CP_ = 47, C^1^(Ph)], 135.9 [d, *J*_CP_ = 22, C^1^(C_6_H_2_)], 133.9 [d, *J*_CP_ = 11, C^2^(Ph)], 130.5 [s, C^4^(Ph)], 128.9 [d, *J*_CP_ = 10, C^3^(Ph)], 122.8 [d, *J*_CP_ = 8, C^3,5^(C_6_H_2_)], 118.8 [d, *J*_CP_ = 9, C^5,3^(C_6_H_2_)], 91.7 (s, Cp), 57.2 (d, *J*_CP_ = 26, PCH_2_), 45.5 (s, *C*Me), 38.4 (s, Me), 35.3 [s, C^1^(^*t*^Bu)], 32.8 [s, C^2^(^*t*^Bu)], 32.0 [d, *J*_CP_ = 8, C^1^(^*t*^Bu)], 31.4 [s, C^2^(^*t*^Bu)], 29.6 (s, Me).

### X-ray Structure Determination of Compounds **4** and **5**

Data collection for these compounds was performed at low temperature on an Oxford Diffraction Xcalibur Nova single crystal diffractometer using Cu Kα radiation. Structure solution and refinements were carried out as described before^11a,13^ to give the residuals shown in Table S1. In compound **4**, the P-bound H atom was located in the Fourier difference map and refined riding on its parent atom, although a restraint had to be applied to the P–H distance to achieve a consistent model. In compound **5**, the P-bound H atom was located and refined analogously but with no restraints.

### Computational Details

DFT calculations were carried out using the GAUSSIAN16 package, the M06L functional, with Grimme D3 dispersion correction, effective core potentials, and their associated double-ζ LANL2DZ basis set for metal atoms and 6-31G* basis for light elements (P, O, C, and H), as described previously.^11a,13^ The effect of the solvent (dichloromethane) on the stability of isomers **8b** and **9** in solution was modeled through the polarized continuum model (PCM) of Tomasi and co-workers,^41^ using the SMD solvation model of Truhlar and co-workers^42^ on the gas-phase optimized structures.

## Supporting Information Available

Crystal data for compounds **4** and **5** (CCDC 2277665 and 2277666), IR and NMR spectra for all new compounds, and results of DFT calculations (PDF)
Cartesian coordinates of all DFT-computed species (XYZ)

## Supplementary Material

om3c00295_si_001.pdf

om3c00295_si_002.xyz
