# Supplementary material for: E–H Bond Cleavage Processes in Reactions of Heterometallic Phosphinidene-Bridged MoRe and MoMn Complexes with Hydrogen and p-Block Element Hydrides
Source: Organometallics. 2023 Aug 31;42(19):2826–38. doi: 10.1021/acs.organomet.3c00295 (PMC10568631; doi:10.1021/acs.organomet.3c00295)
Supplement: Supplementary file 1 — om3c00295_si_001.pdf [file om3c00295_si_001.pdf]

Supporting Information

E–H Bond Cleavage Processes in Reactions of Heterometallic Phosphinidene-Bridged MoRe and MoMn Complexes with Hydrogen and p-Block Element Hydrides

M. Angeles Alvarez, M. Esther García, Daniel García-Vivó,* Miguel A. Ruiz,* and Patricia Vega

Departamento de Química Orgánica e Inorgánica/IUQOEM, Universidad de Oviedo, E-33071 Oviedo, Spain.

Corresponding Author E-mail: garciavdaniel@uniovi.es (D.G.V.), mara@uniovi.es (M.A.R).

Table of Contents

		page
1.	Crystal data for new compounds (Table S1)	S-3
2.	IR and NMR data for compound **2a** (Figs. S1 to S4)	S-4
3.	IR and NMR data for compound **2b** (Figs. S5 to S8)	S-6
4.	IR and NMR data for compound **Na-3** (Figs. S9 and S10)	S-8
5.	IR and NMR data for compound **4** (Figs. S11 to S14)	S-9
6.	IR and NMR data for compound **5** (Figs. S15 to S18)	S-11
7.	IR and NMR data for compound **6** (Figs. S19 to S21)	S-13
8.	IR and NMR data for compound **7a** (Figs. S22 to S27)	S-14
9.	IR and NMR data for compound **7b** (Figs. S28 to S32)	S-18
10.	IR and NMR data for compound **8a** (Figs. S33 to S36)	S-21
11.	IR and NMR data for compounds **8b** and **9** (Figs. S37 to S41)	S-23
12.	IR and NMR data for compound **10** (Figs. S42 to S45)	S-26
13.	IR and NMR data for compound **11** (Figs. S46 to S49)	S-28
14.	DFT optimized structures of hydrogenation derivatives of **1a-Ph** (Figure S50).	S-30
15.	Selected DFT-computed molecular orbitals of **1a-Ph** (Figure S51)	S-31

Table S1. Crystal Data for New Compounds

	**4·**	**5**
mol formula	$C_{50}H_{56}AuMoO_6P_2Re$	$C_{35}H_{40}MoO_6PReS$
mol wt	1294.01	901.85
cryst syst	triclinic	orthorhombic
space group	*P*–1	*P*bca
radiation (*λ*, Å)	1.54184	1.54184
a, Å	10.7402(5)	16.8887(1)
b, Å	11.8815(8)	16.2291(1)
c, Å	20.5937(12)	26.1448(2)
α, deg	76.707(5)	90
β, deg	77.242(4)	90
γ, deg	74.492(5)	90
V, $Å^3$	2428.6(3)	7165.99(8)
Z	2	8
calcd density, g cm^{-3}	1.770	1.670
absorp coeff, mm^{-1}	13.408	10.678
temperature, K	130.0(1)	150.2(3)
θ range (deg)	3.93 / 69.33	3.38 / 69.50
index ranges (*h*, *k*, *l*)	–10, 13; –14, 14<br>–24, 23	–20, 20; –19, 18<br>–24, 31
no. of reflns collected	19561	20044
no. of indep reflns (R_{int})	8906 (0.0326)	6632 (0.0285)
reflns with $I > 2\sigma(I)$	7709	6279
R indexes<br>[data with $I > 2\sigma(I)$][a]	$R_1 = 0.0338$<br>$wR_2 = 0.0854^b$	$R_1 = 0.0488$<br>$wR_2 = 0.1193^c$
R indexes (all data)[a]	$R_1 = 0.0405$<br>$wR_2 = 0.0892^b$	$R_1 = 0.0503$<br>$wR_2 = 0.1214^c$
GOF	1.111	1.123
no. of restraints/params	1 / 565	0 / 418
$\Delta\rho$(max., min.), $eÅ^{-3}$	1.264 / –1.741	2.191 / –0.551
CCDC deposition no	2277665	2277666

[a] $R = \Sigma||F_o| - |F_c|| / \Sigma|F_o|$. $wR = [\Sigma w(|F_o|^2 - |F_c|^2)^2 / \Sigma w|F_o|^2]^{1/2}$. $w = 1/[\sigma^2(F_o^2) + (aP)^2 + bP]$ where $P = (F_o^2 + 2F_c^2)/3$. [b] $a = 0.0390$, $b = 3.6210$. [c] $a = 0.0860$, $b = 0.0000$.

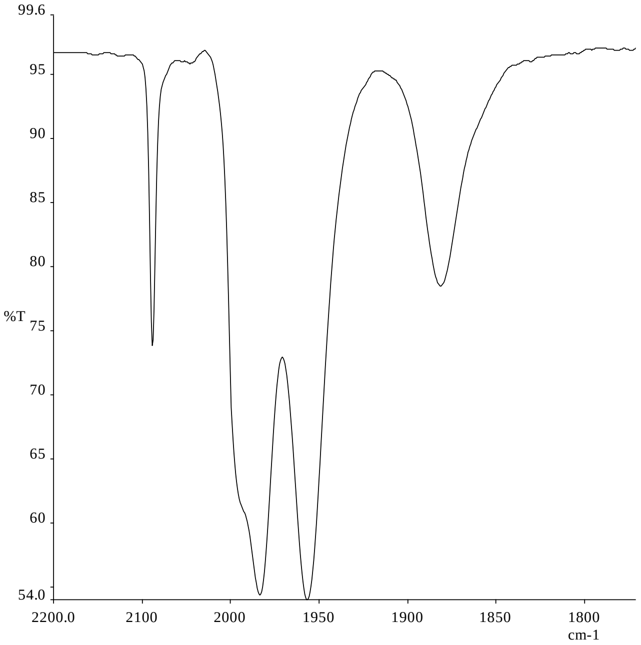

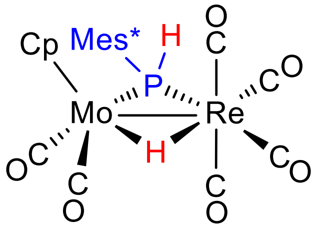

2a

Figure S1. IR spectrum of compound **2a** in dichloromethane solution.

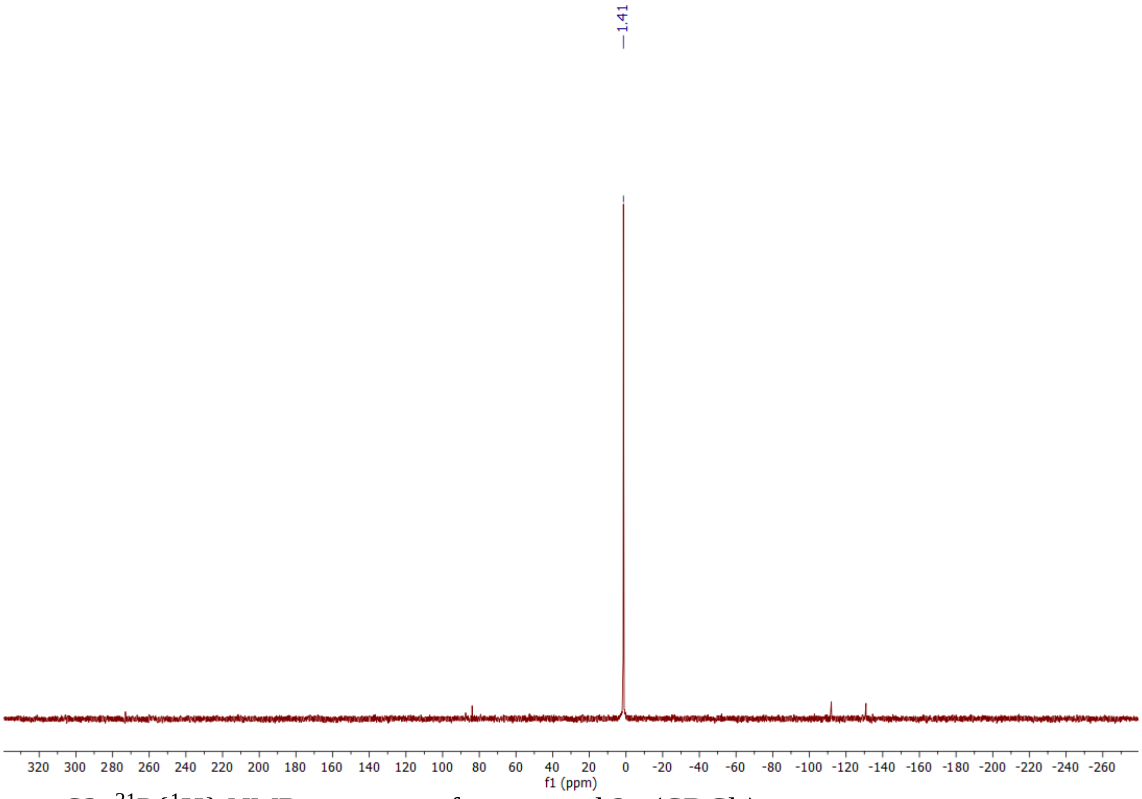

Figure S2. ^{31}P{^{1}H} NMR spectrum of compound **2a** (CDCl$_2$).

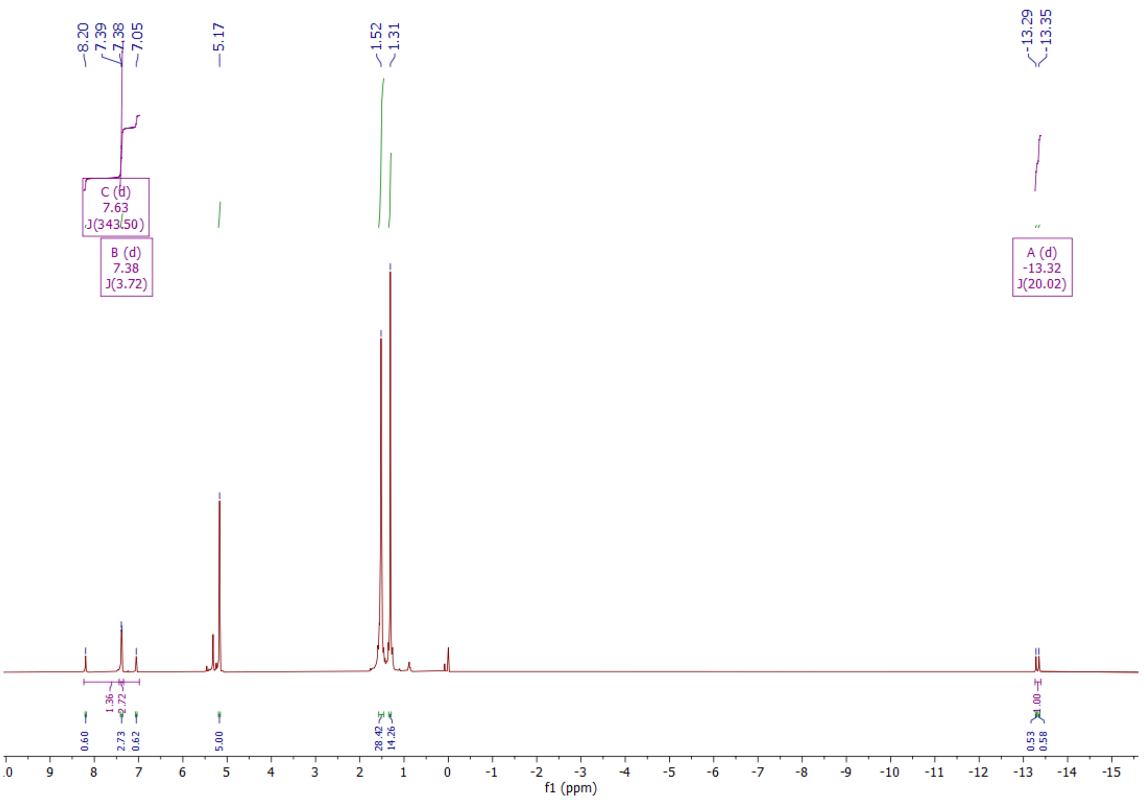

Figure S3. ^{1}H NMR spectrum of compound **2a** (CD_2Cl_2).

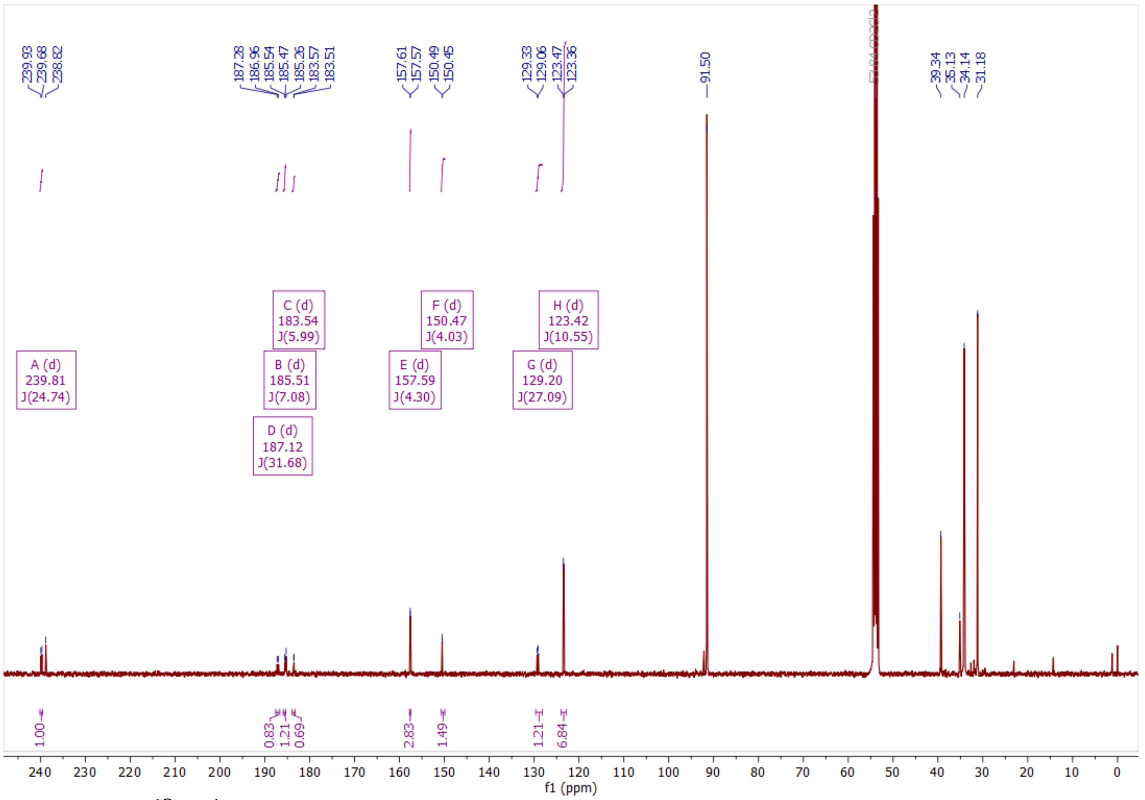

Figure S4. $^{13}C\{^{1}H\}$ NMR spectrum of compound **2a** (CD_2Cl_2).

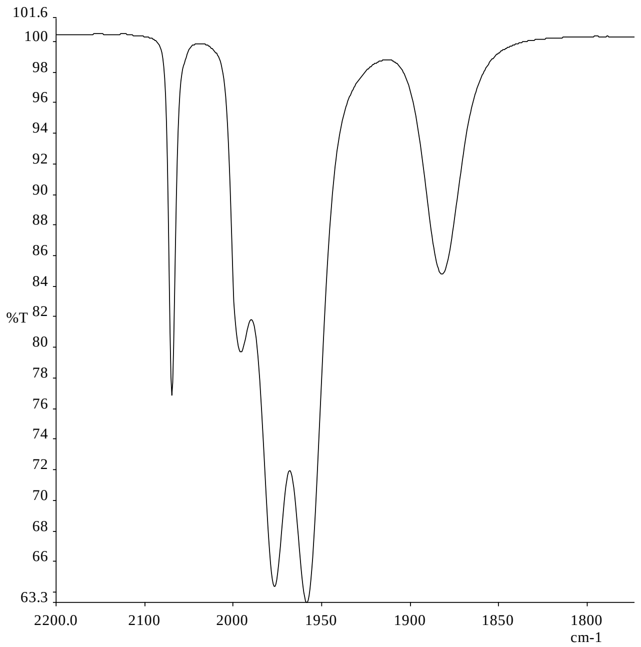

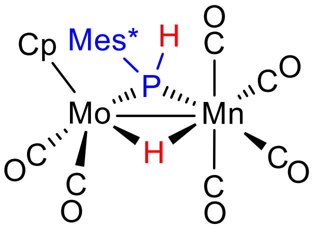

2b

Figure S5. IR spectrum of compound **2b** in dichloromethane solution.

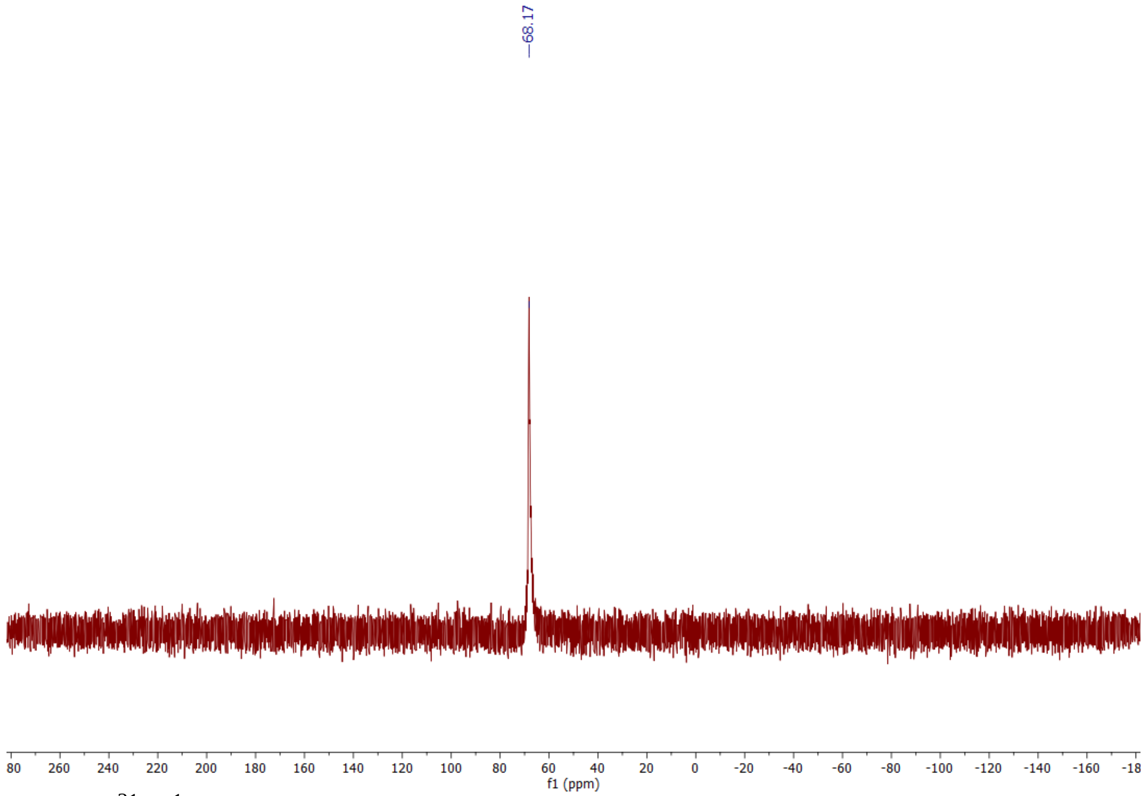

Figure S6. $^{31}P\{^1H\}$ NMR spectrum of compound **2b** (CD_2Cl_2).

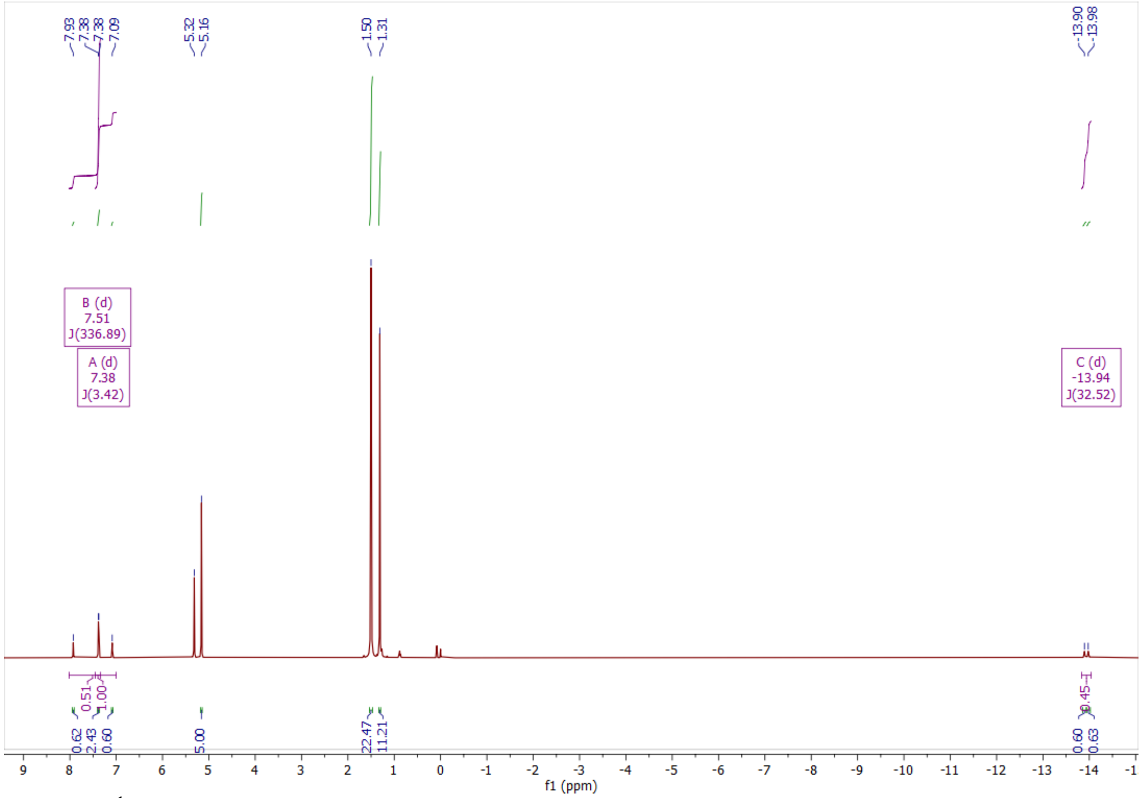

Figure S7. ^{1}H NMR spectrum of compound **2b** (CD_2Cl_2).

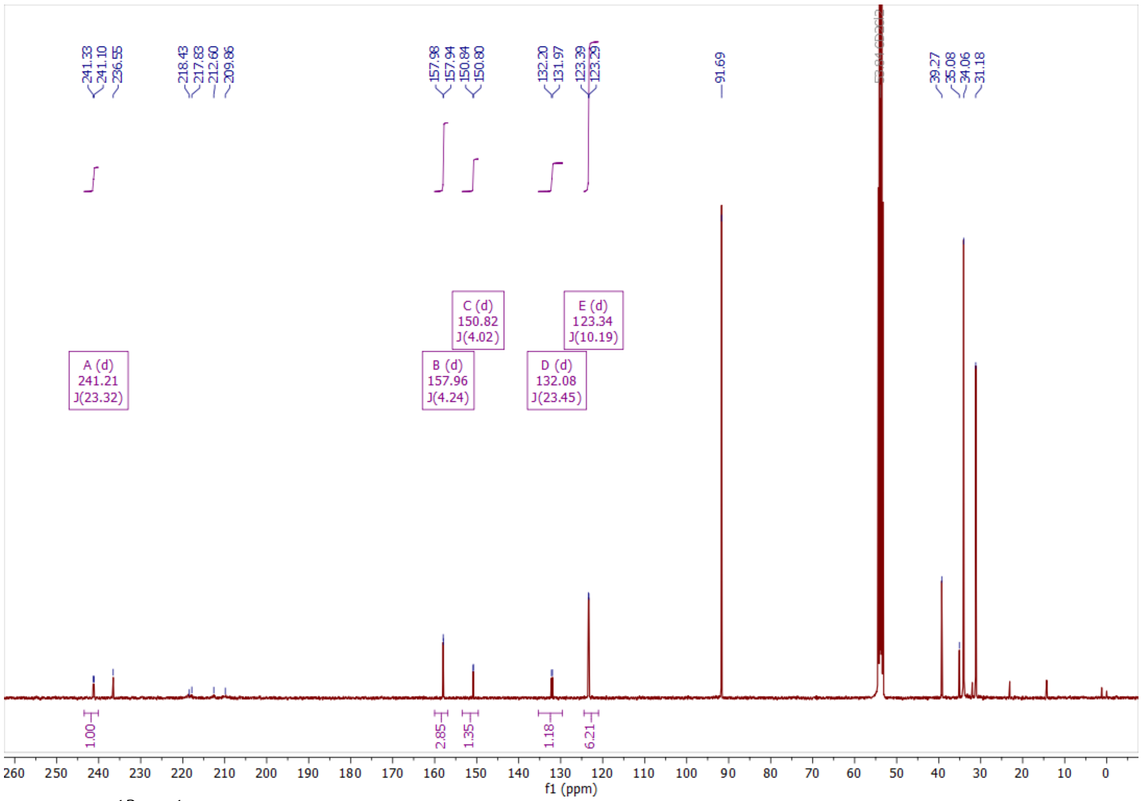

Figure S8. $^{13}C\{^{1}H\}$ NMR spectrum of compound **2b** (CD_2Cl_2).

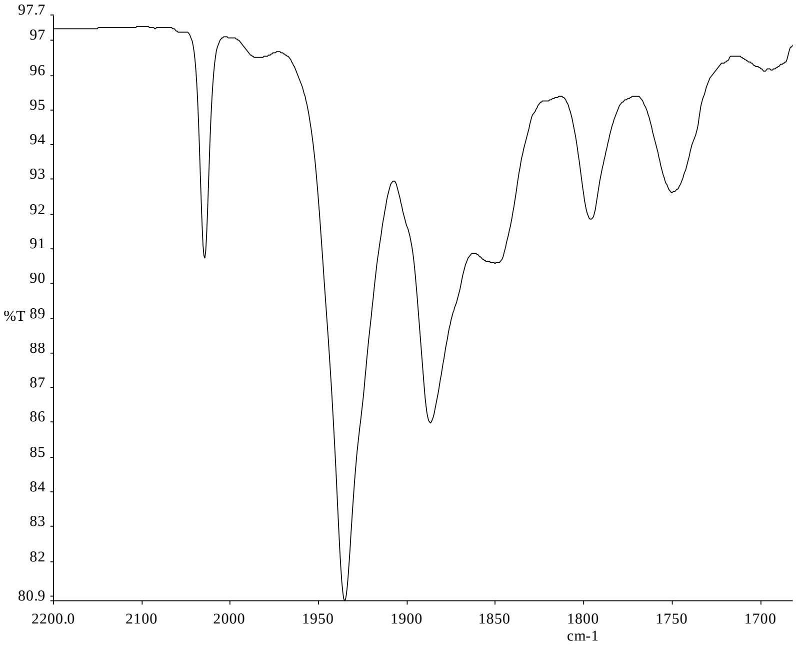

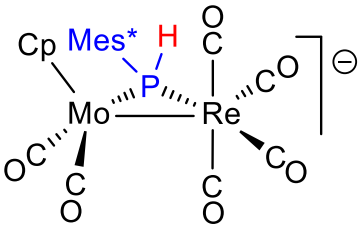

3

Figure S9. IR spectrum of compound **Na-3** in tetrahydrofuran solution.

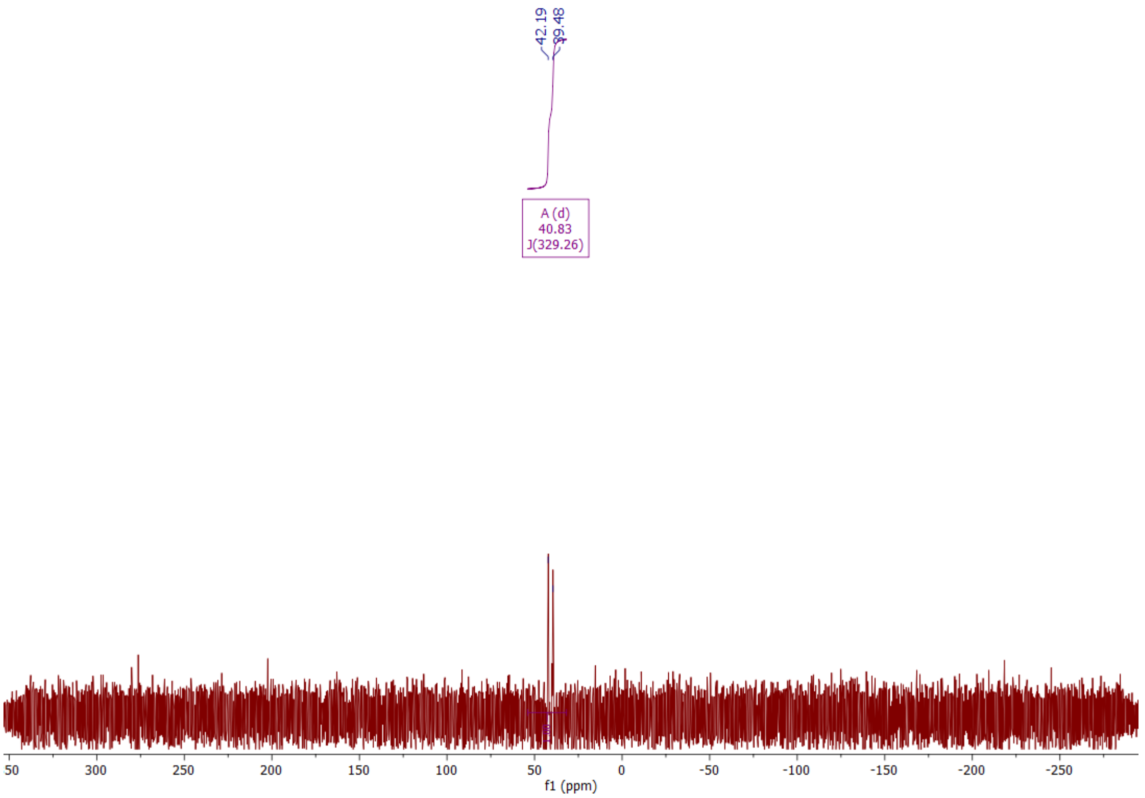

Figure S10. ^{31}P NMR spectrum of compound **Na-3** (tetrahydrofuran).

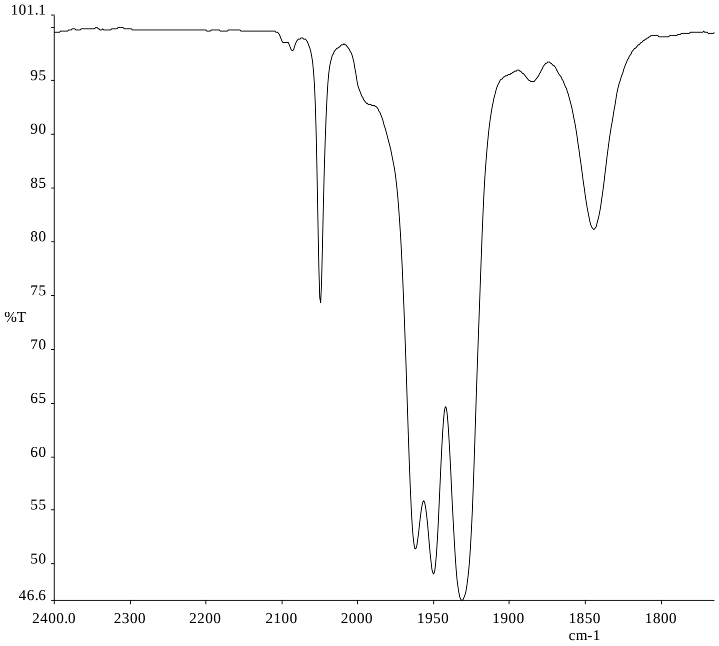

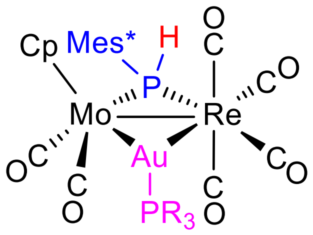

4

Figure S11. IR spectrum of compound **4** in toluene solution.

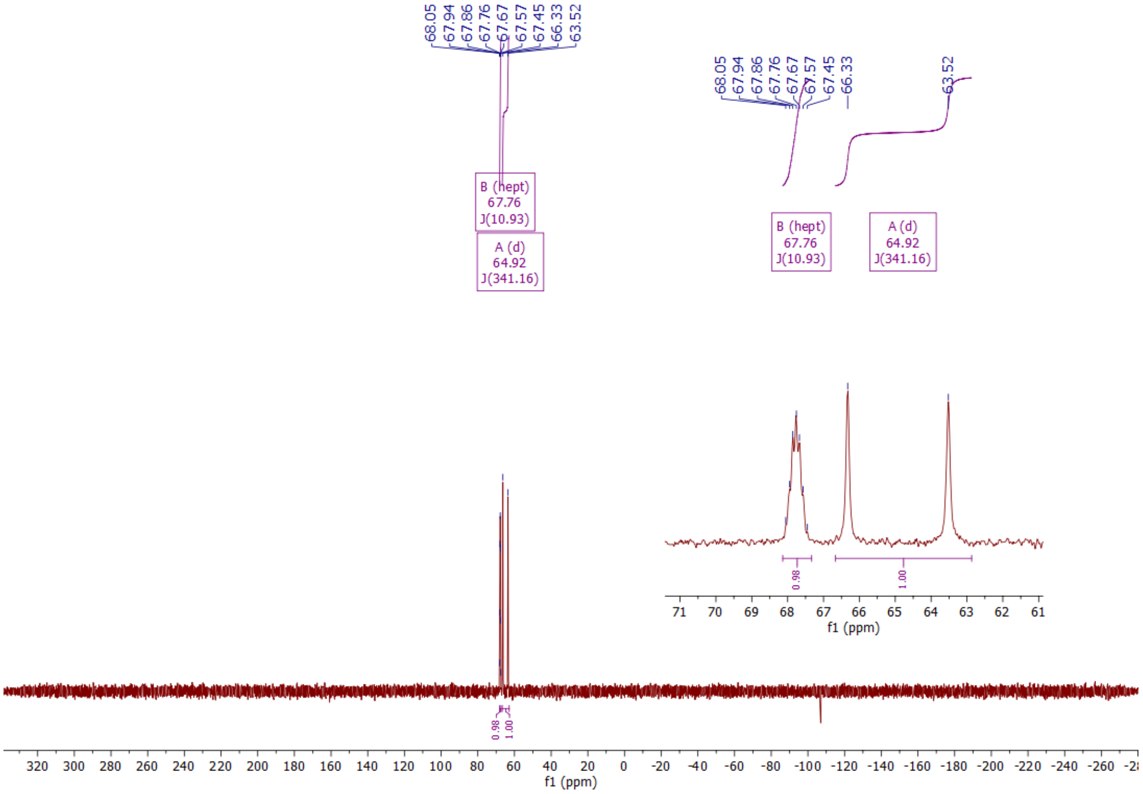

Figure S12. ^{31}P NMR spectrum of compound **4** (C_6D_6).

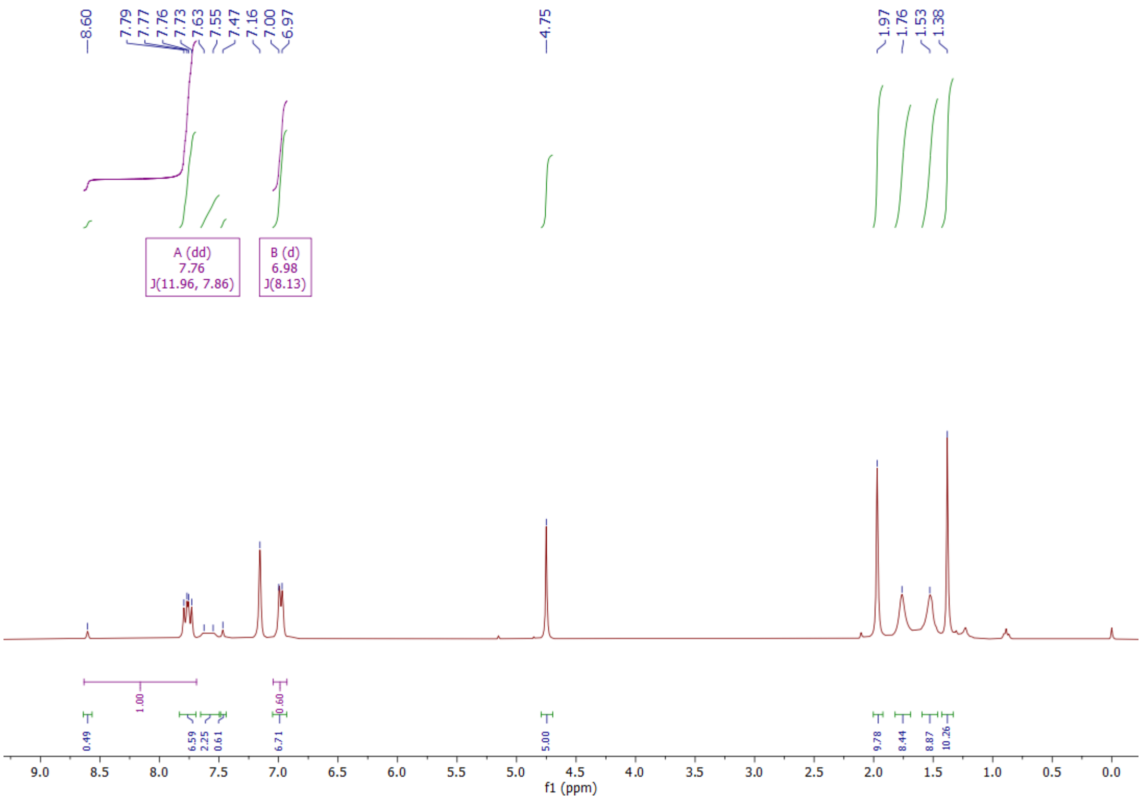

Figure S13. ^{1}H NMR spectrum of compound **4** (C_6D_6).

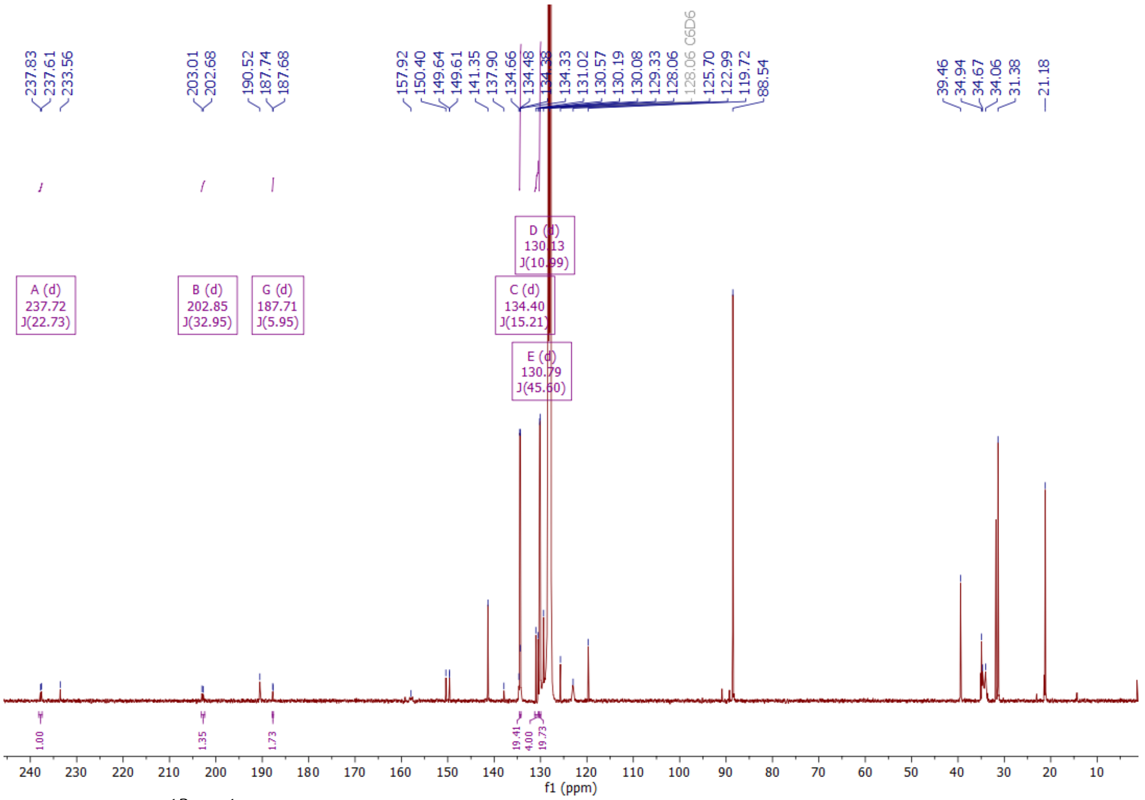

Figure S14. $^{13}C\{^{1}H\}$ NMR spectrum of compound **4** (C_6D_6).

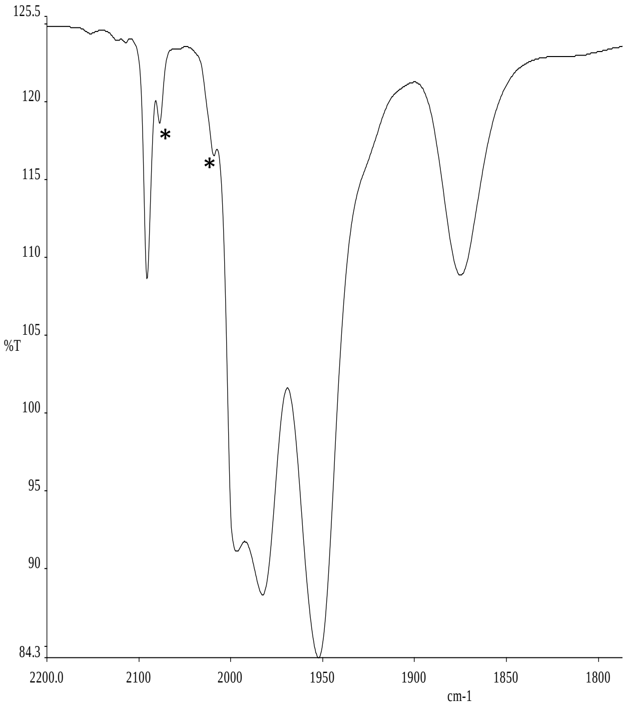

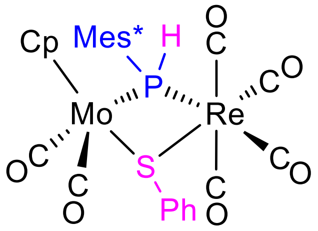

5

Figure S15. IR spectrum of compound **5** in dichloromethane solution (* bands of unidentified species).

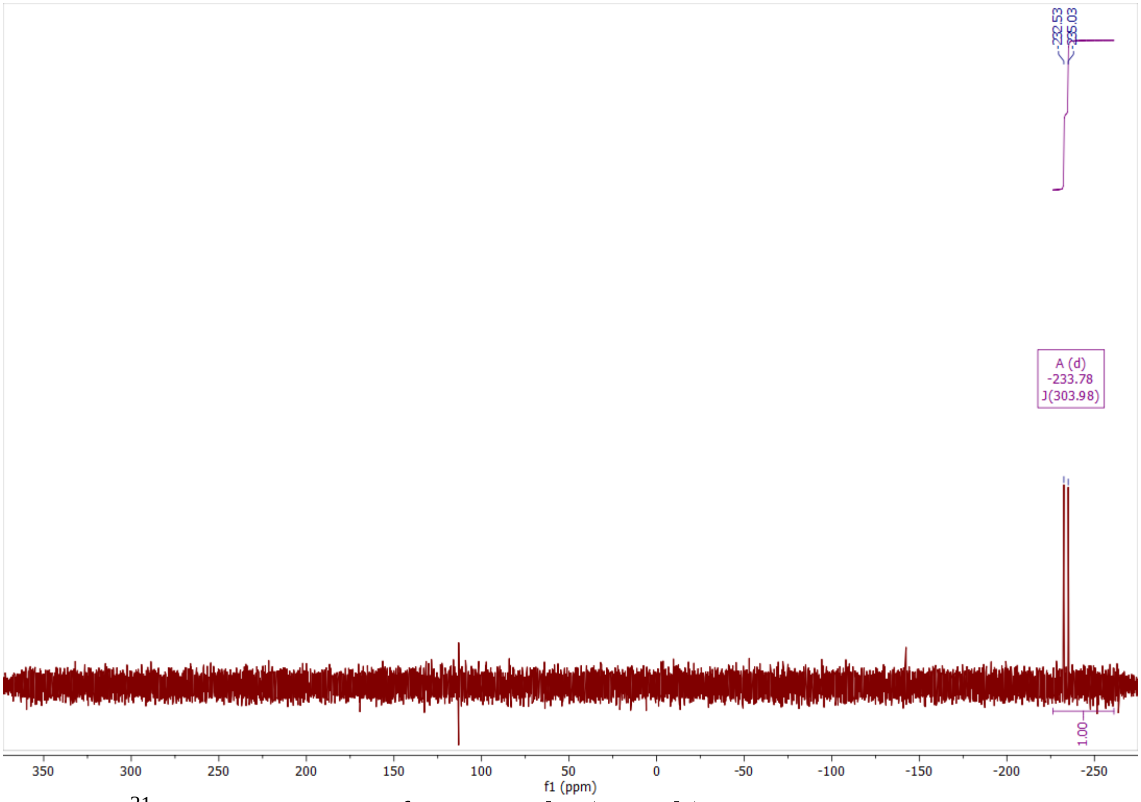

Figure S16. ^{31}P NMR spectrum of compound **5** (CD_2Cl_2).

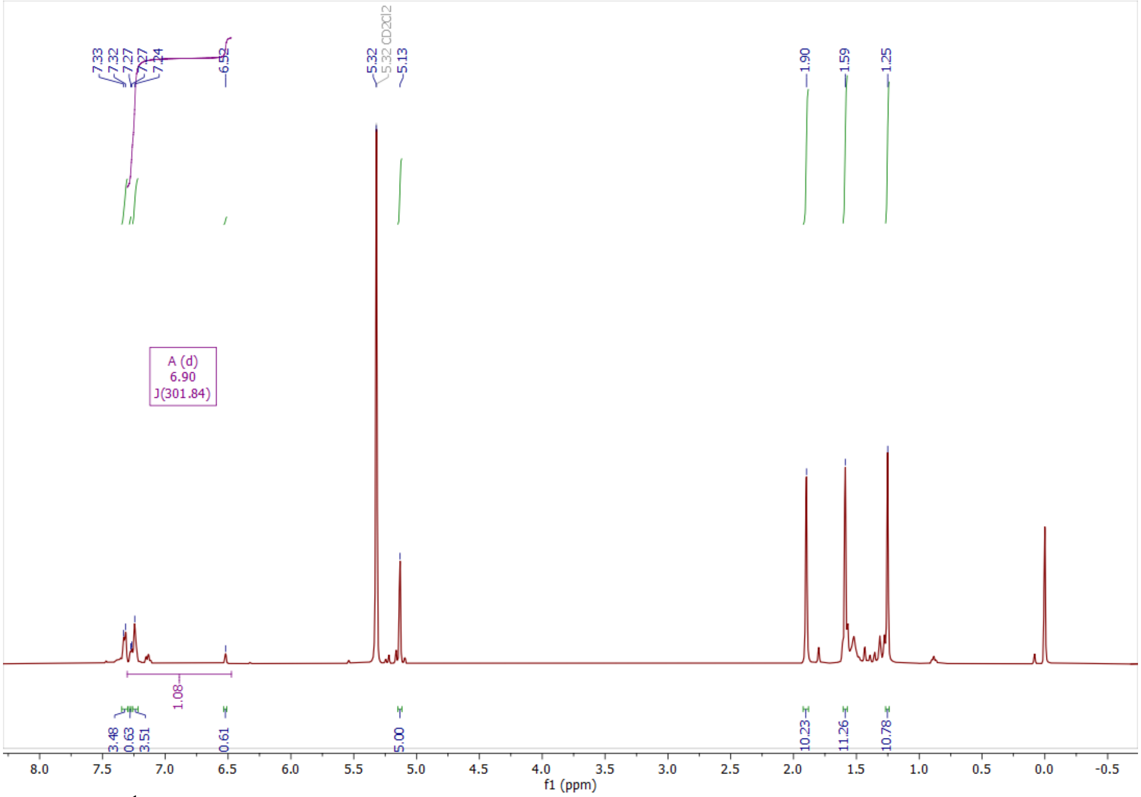

Figure S17. ^{1}H NMR spectrum of compound **5** (CD_2Cl_2).

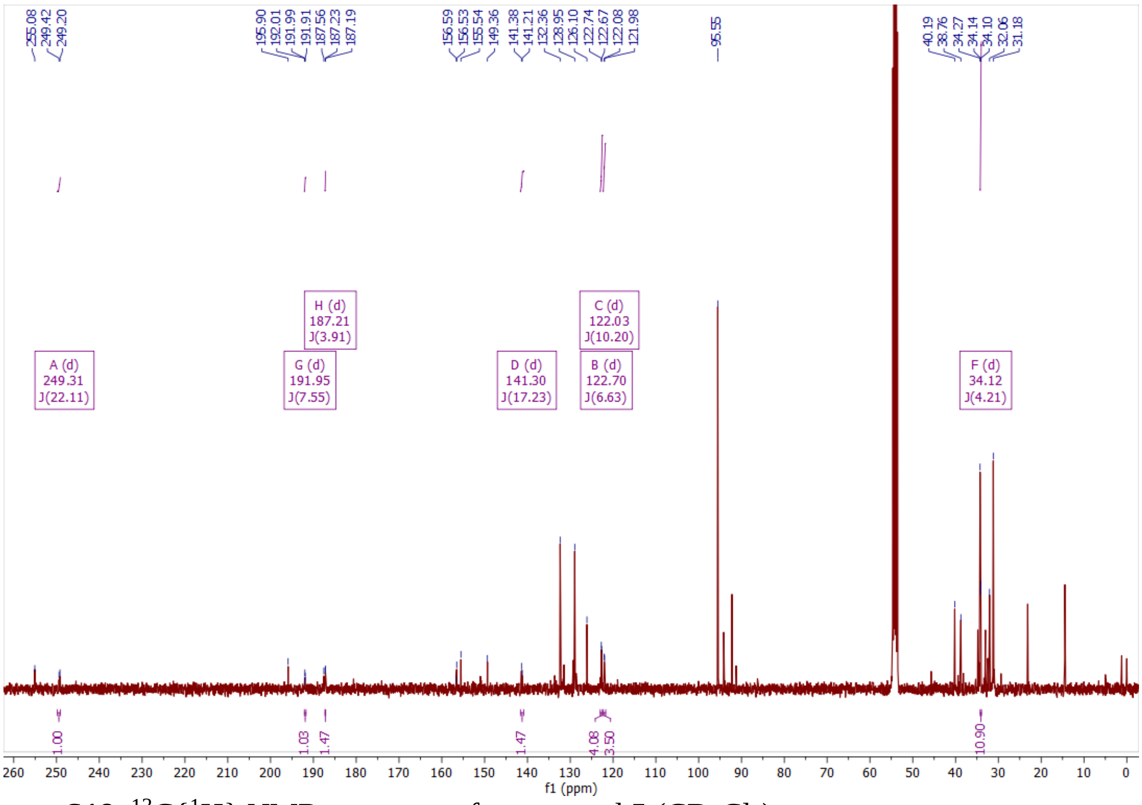

Figure S18. $^{13}C\{^{1}H\}$ NMR spectrum of compound **5** (CD_2Cl_2).

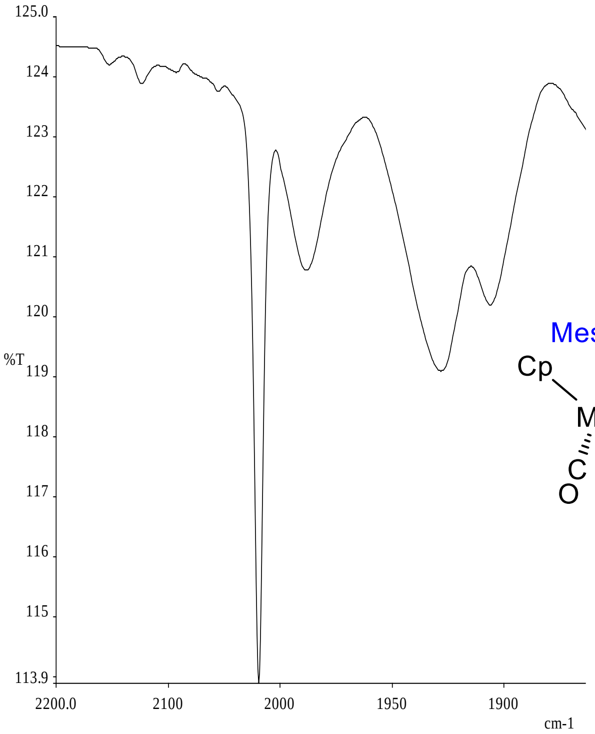

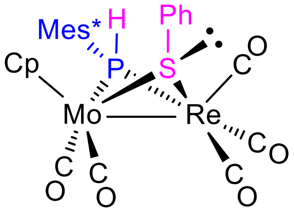

***syn*-6**

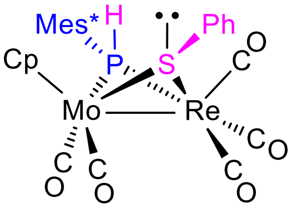

***anti*-6**

Figure S19. IR spectrum of compound **6** in dichloromethane solution.

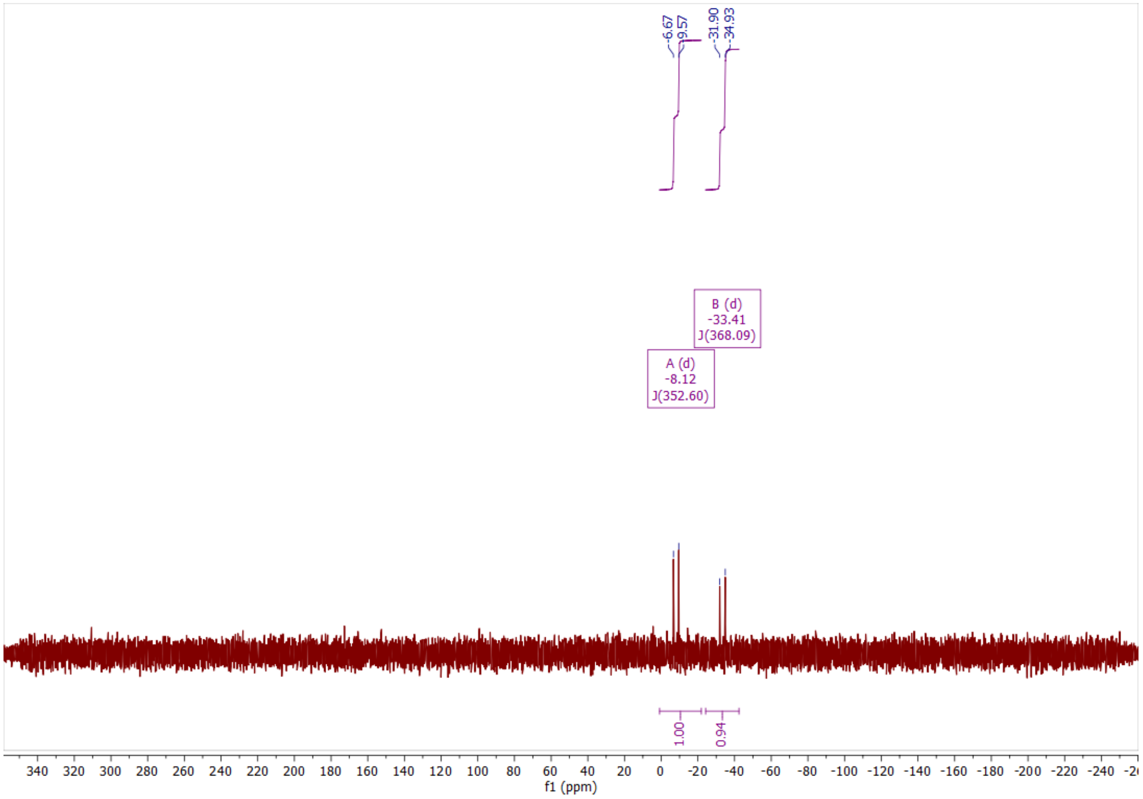

Figure S20. ^{31}P NMR spectrum of compound **6** (CD_2Cl_2; *syn* and *anti* isomers)

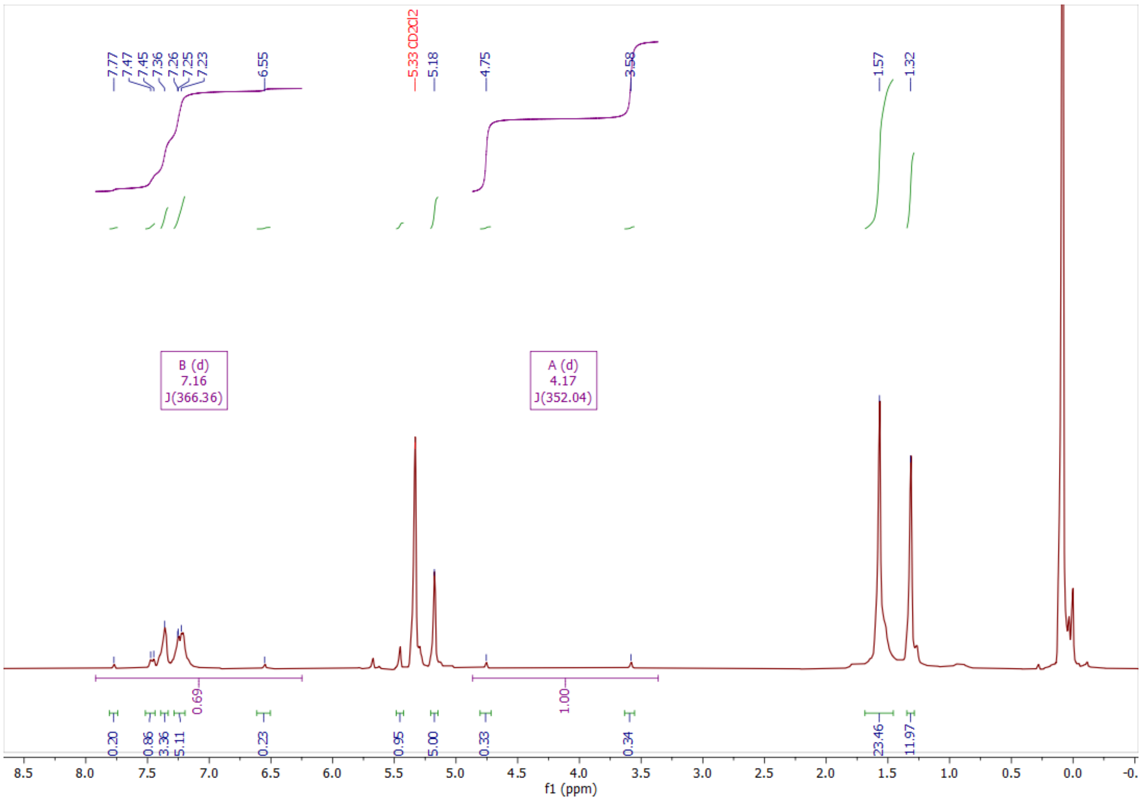

Figure S21. ^{1}H NMR spectrum of compound **6** (CD_2Cl_2; *syn* and *anti* isomers).

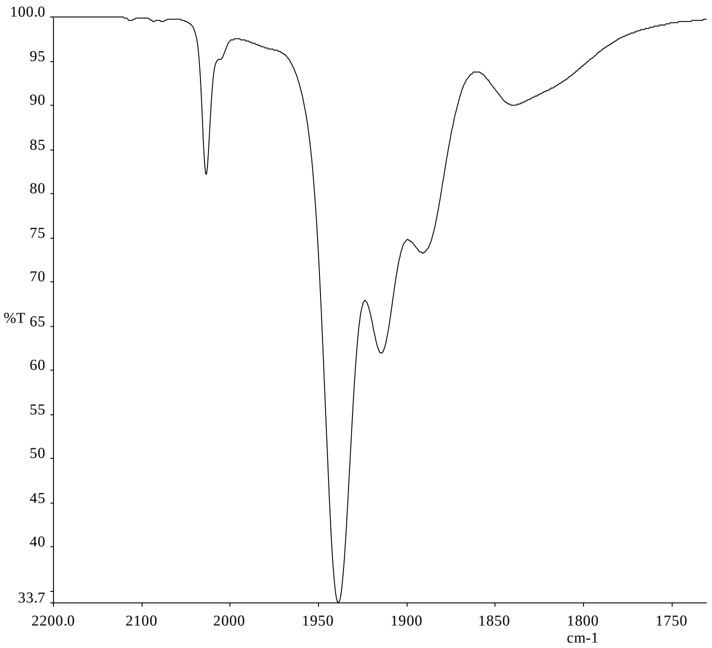

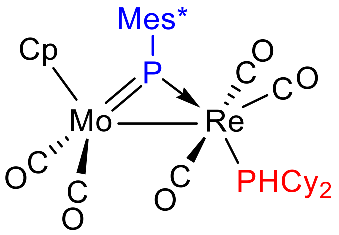

7a

Figure S22. IR spectrum of compound **7a** in dichloromethane solution.

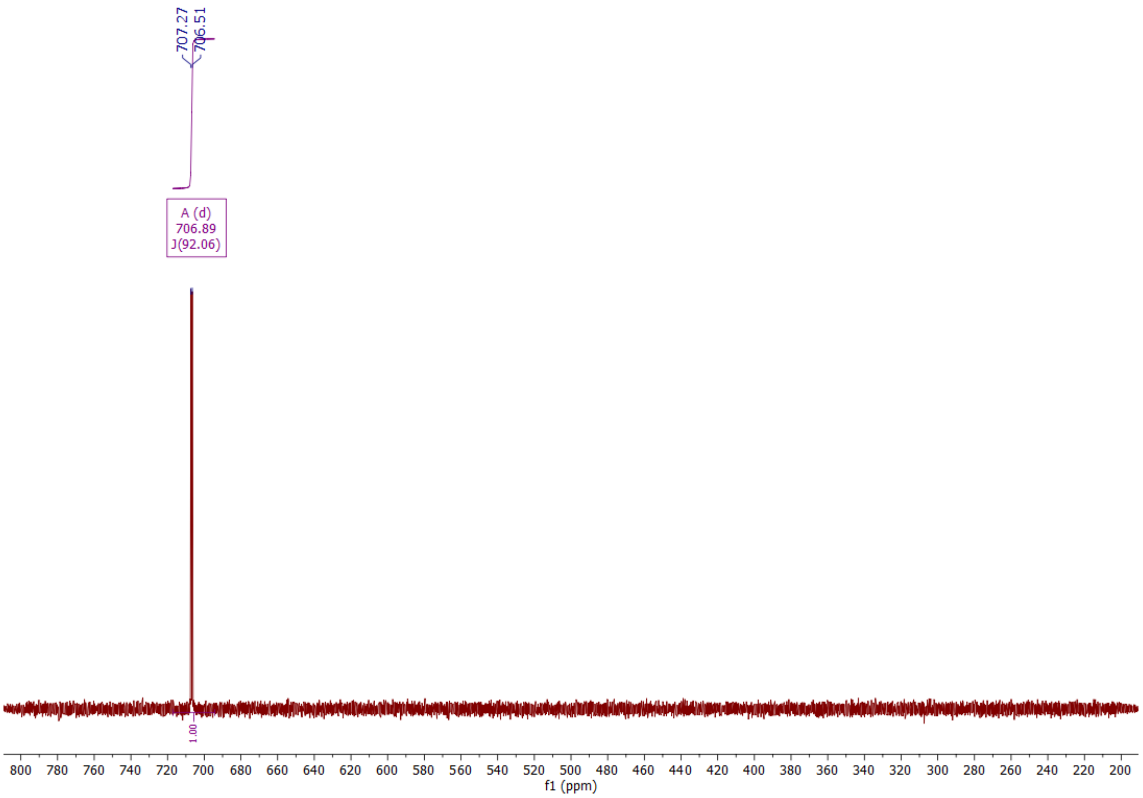

Figure S23. $^{31}P\{^{1}H\}$ NMR spectrum of compound **7a** (CD_2Cl_2, high-frequency region).

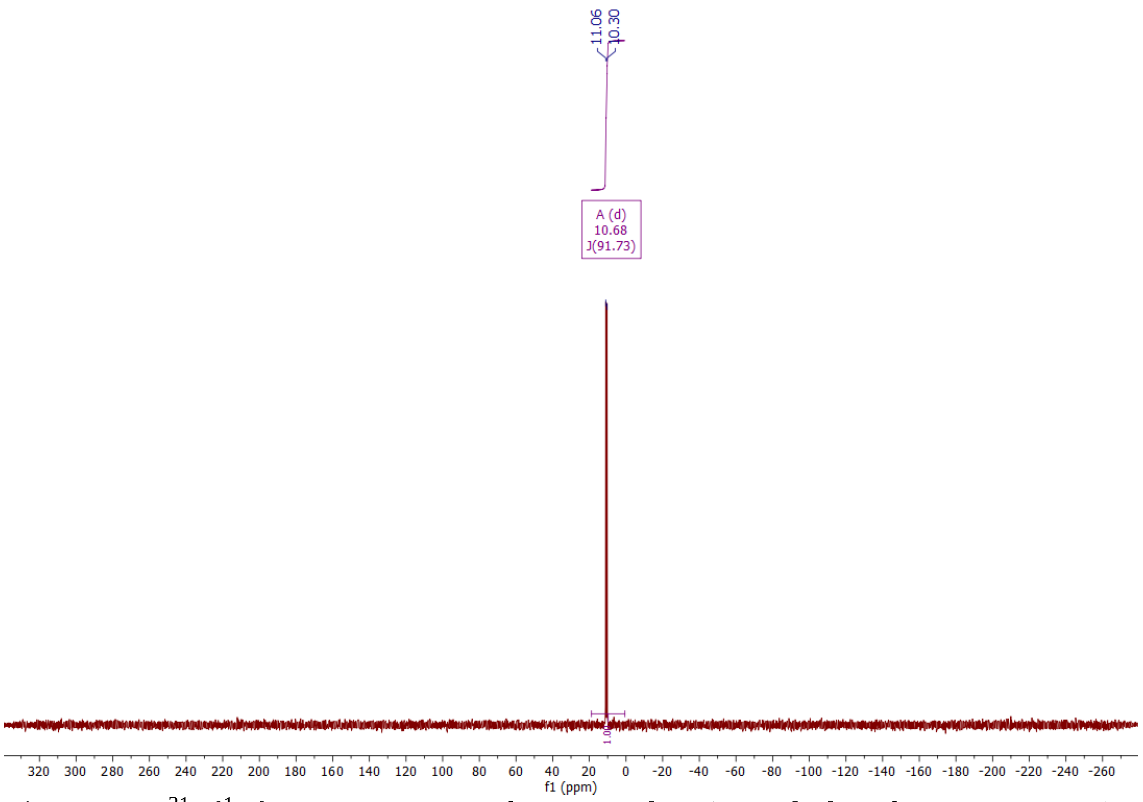

Figure S24. $^{31}P\{^{1}H\}$ NMR spectrum of compound **7a** (CD_2Cl_2, low-frequency region).

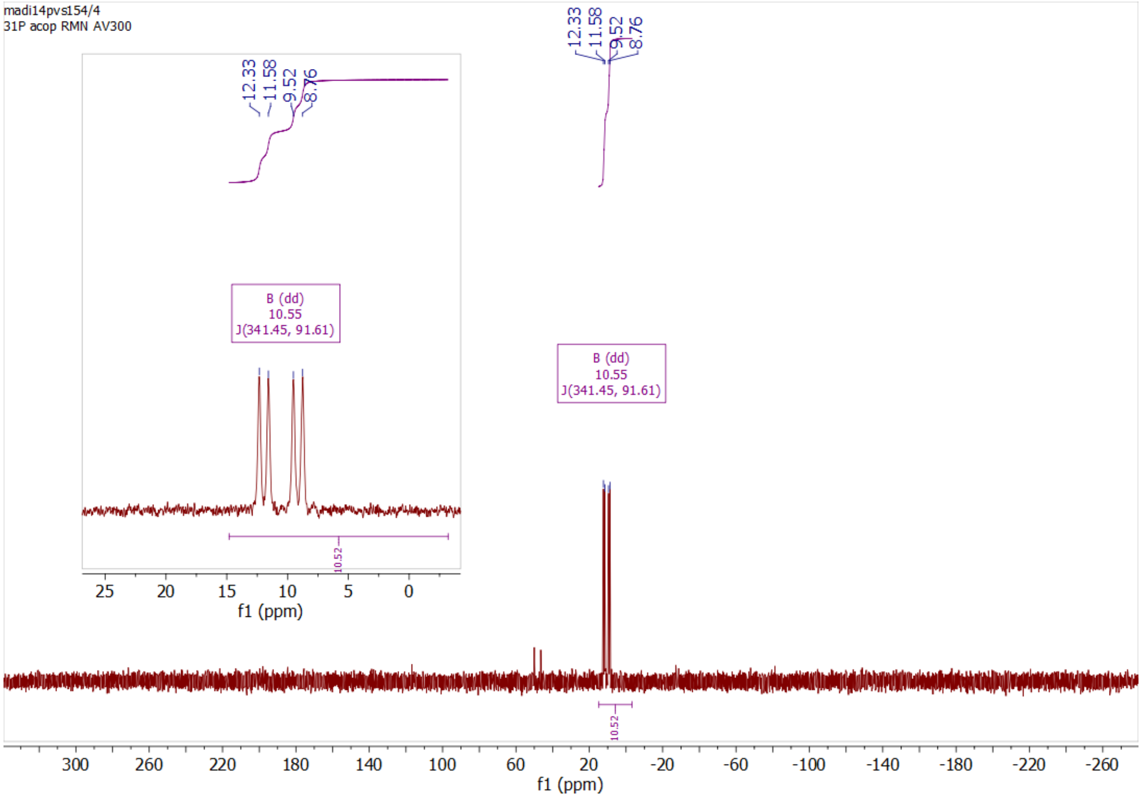

Figure S25. ^{31}P NMR spectrum of compound **7a** (CD_2Cl_2, low-frequency region).

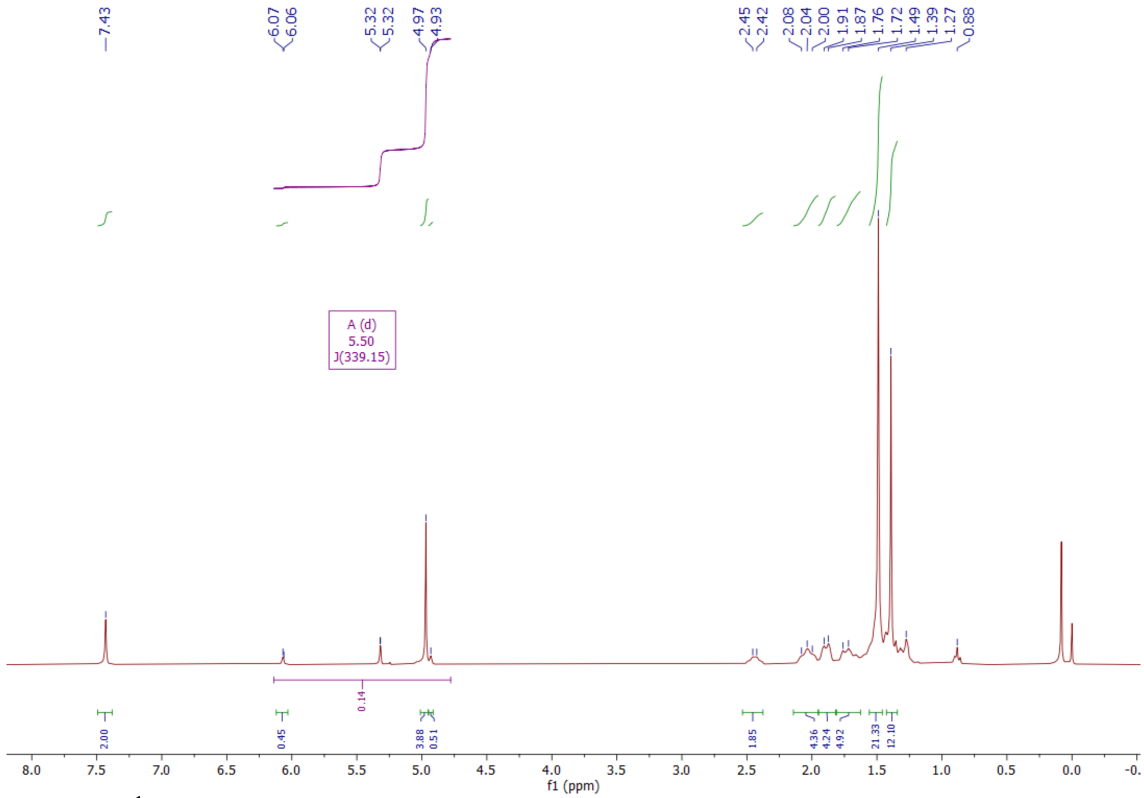

Figure S26. ^{1}H NMR spectrum of compound **7a** (CD_2Cl_2).

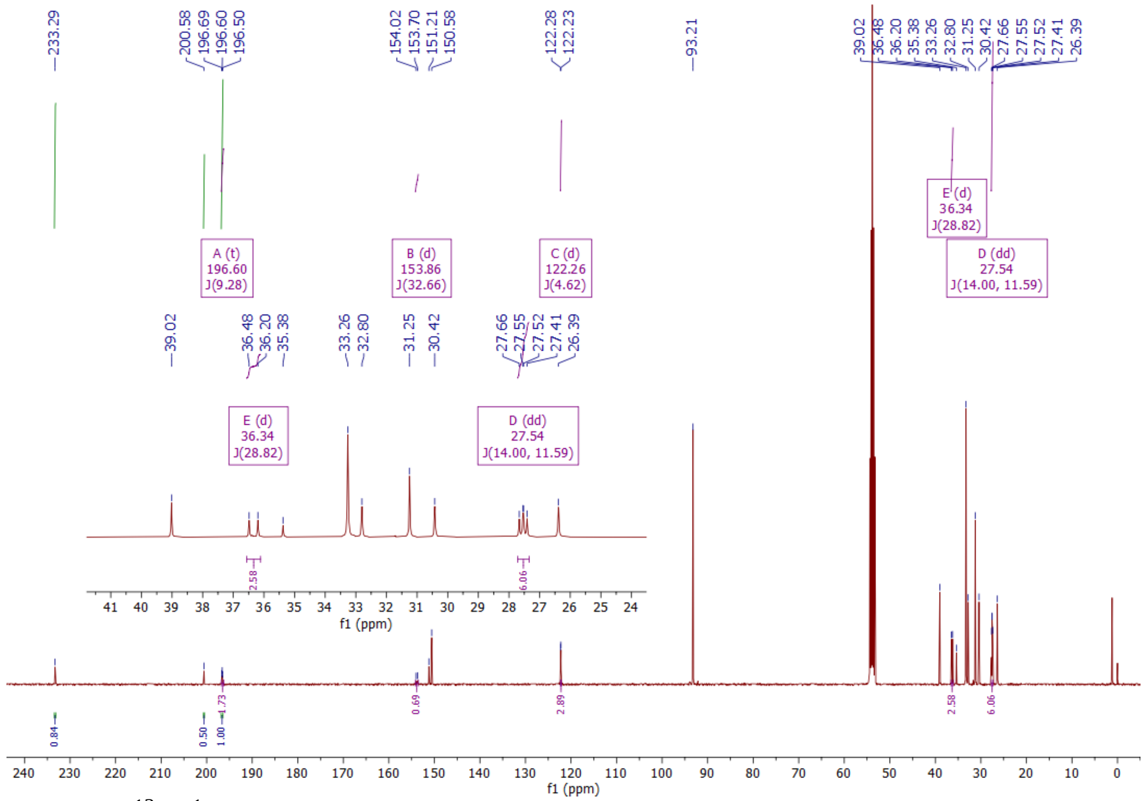

Figure S27. $^{13}C\{^1H\}$ NMR spectrum of compound **7a** (CD_2Cl_2)

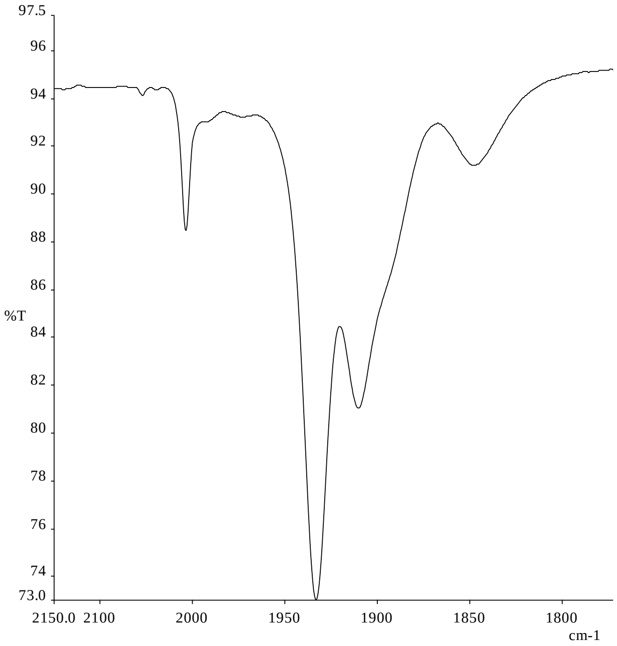

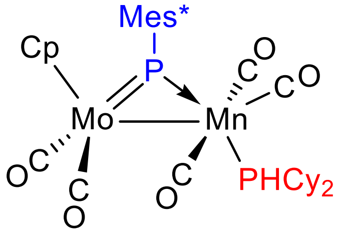

7b

Figure S28. IR spectrum of compound **7b** in dichloromethane solution.

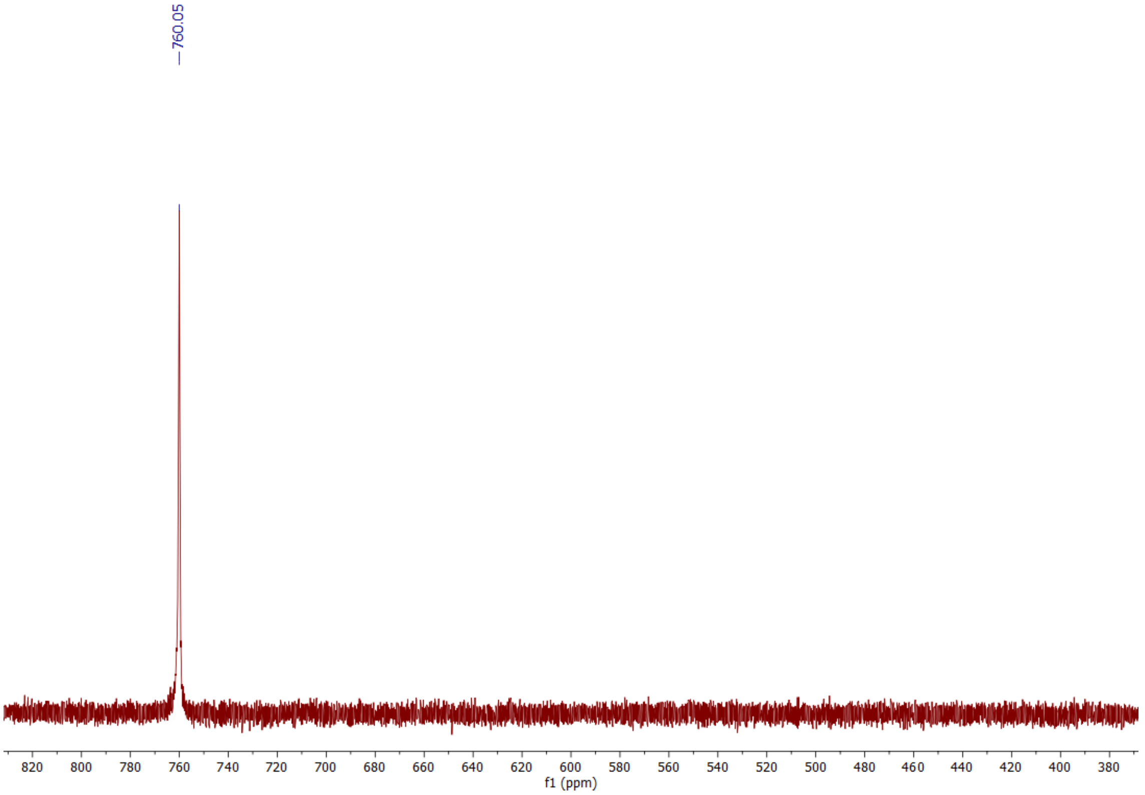

Figure S29. $^{31}P\{^{1}H\}$ NMR spectrum of compound **7b** (CD_2Cl_2, 233 K, high-frequency region).

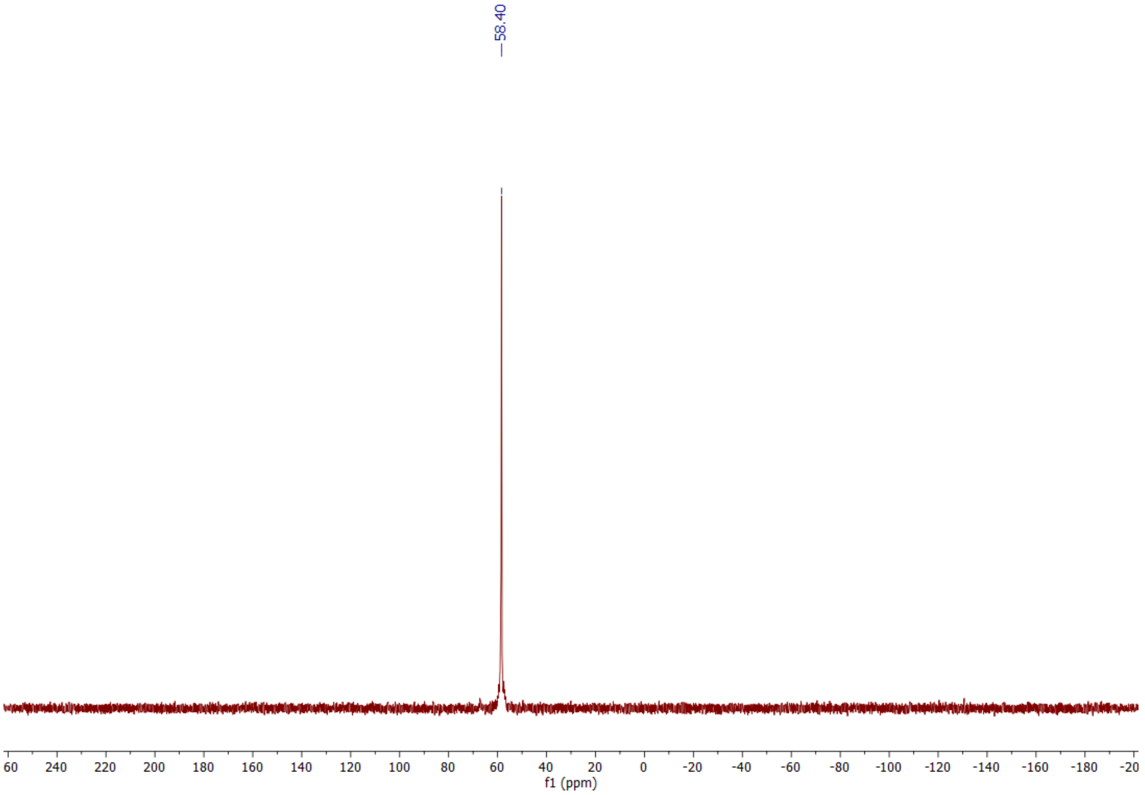

Figure S30. $^{31}P\{^{1}H\}$ NMR spectrum of compound **7b** (CD_2Cl_2, 233 K, low-frequency region).

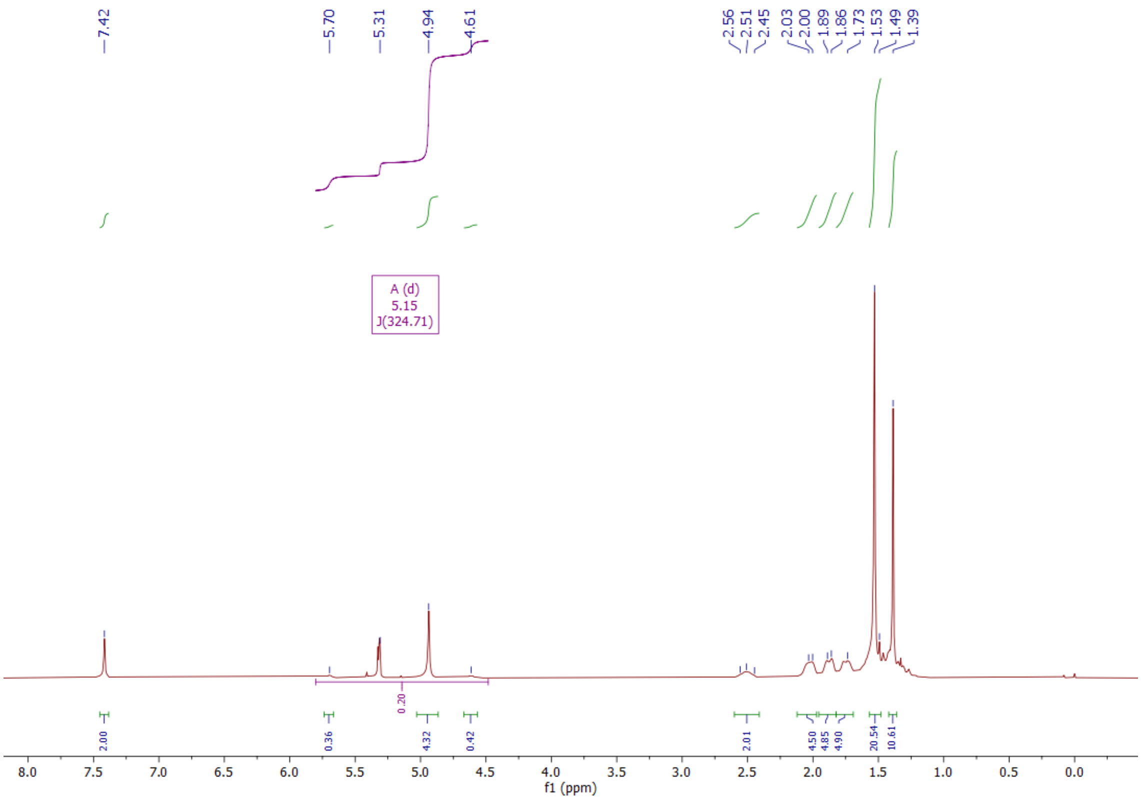

Figure S31. ^{1}H NMR spectrum of compound **7b** (CD_2Cl_2).

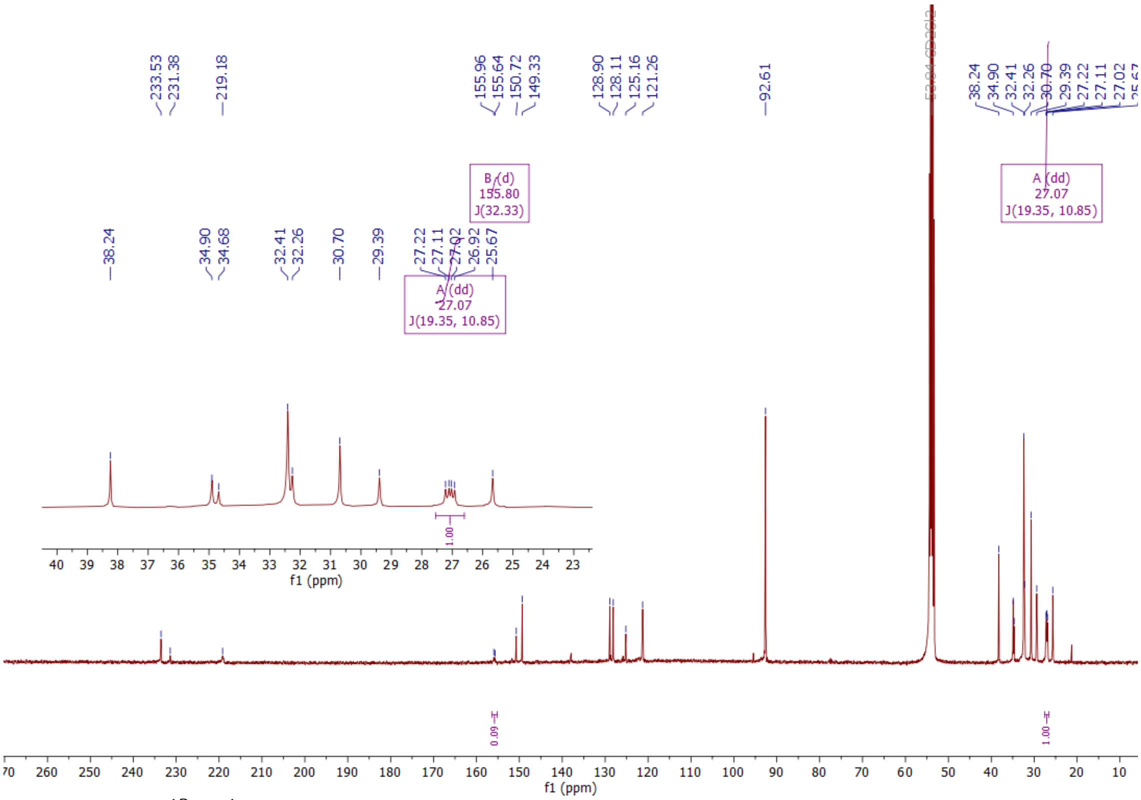

Figure S32. $^{13}C\{^{1}H\}$ NMR spectrum of compound **7b** (CD_2Cl_2, 233 K).

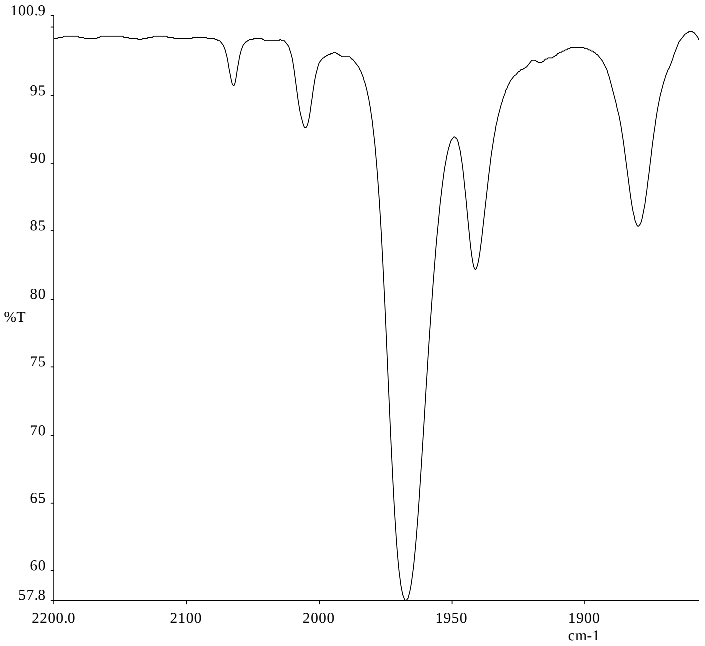

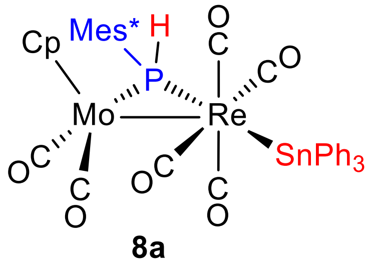

8a

Figure S33. IR spectrum of compound **8a** in toluene solution.

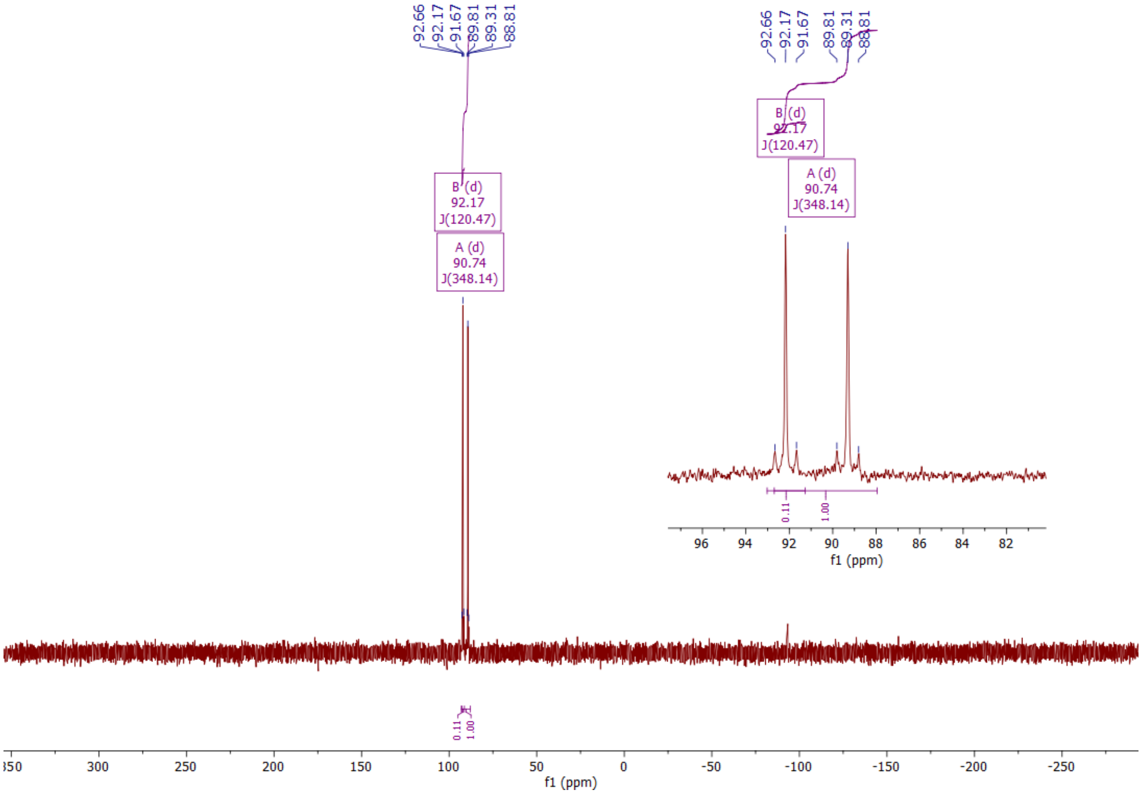

Figure S34. ^{31}P NMR spectrum of compound **8a** (toluene-d_8).

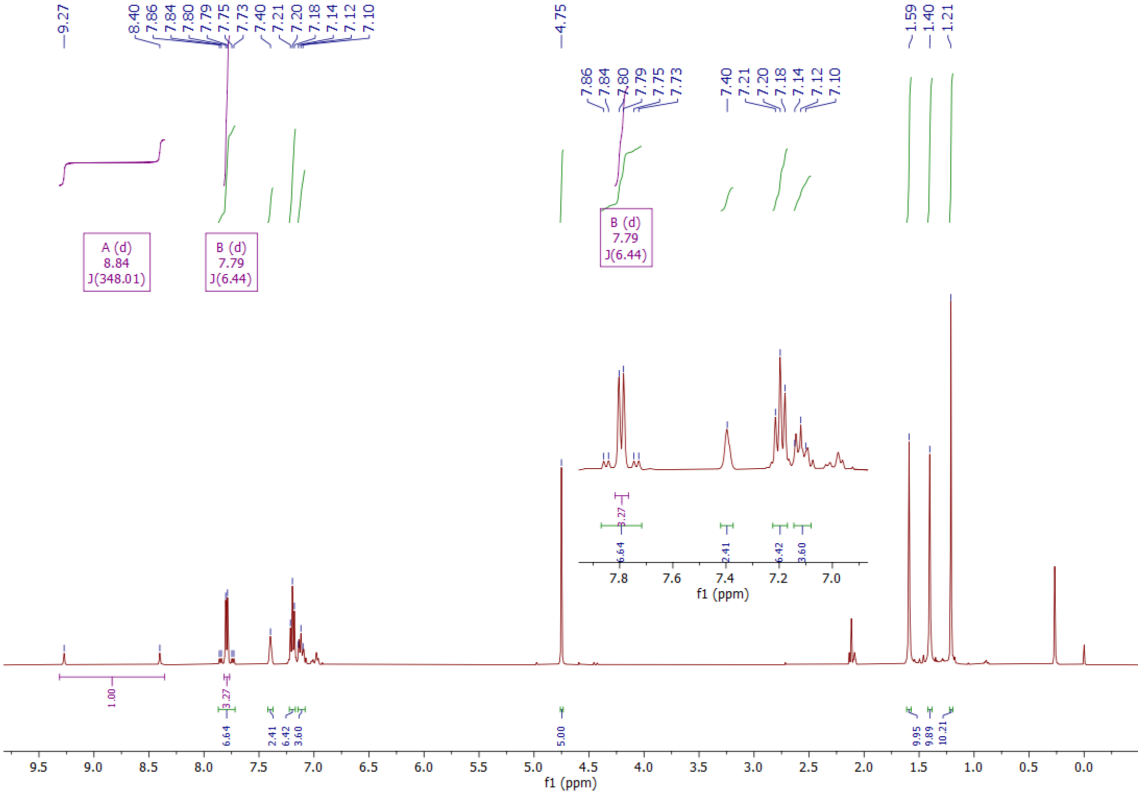

Figure S35. ^{1}H NMR spectrum of compound **8a** (toluene-d_8).

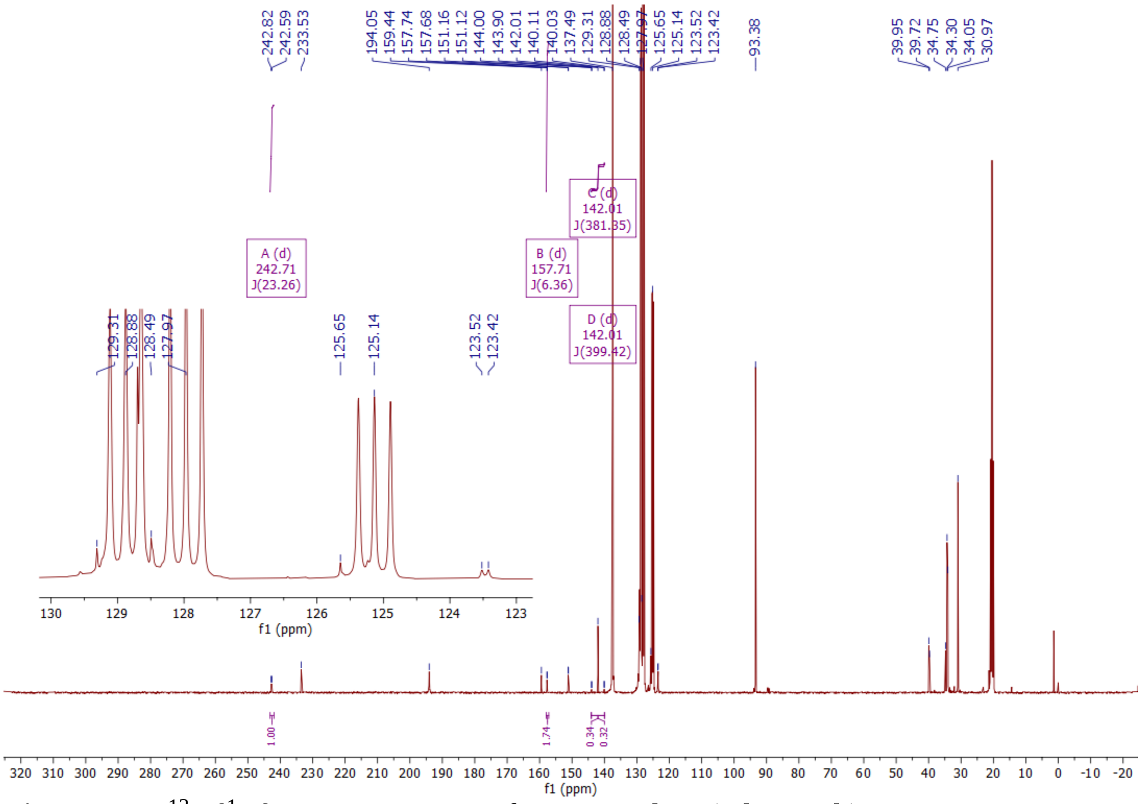

Figure S36. $^{13}C\{^{1}H\}$ NMR spectrum of compound **8a** (toluene-d_8).

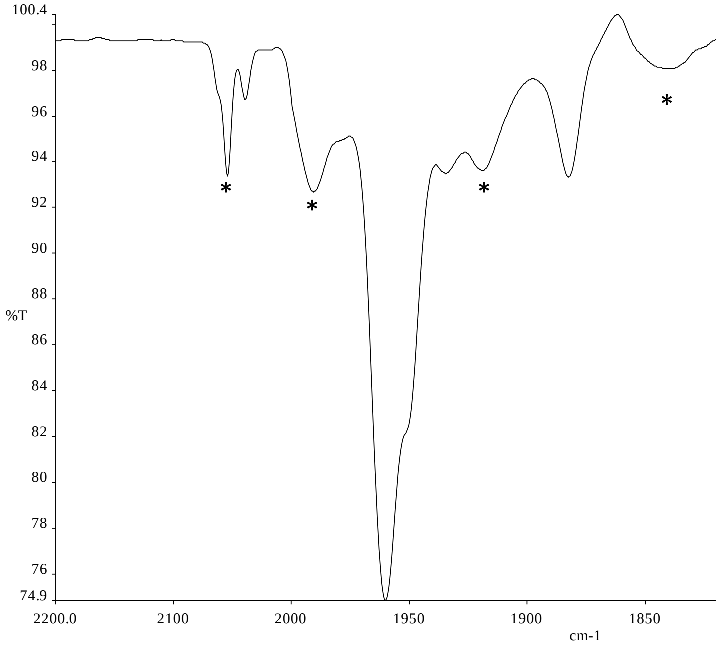

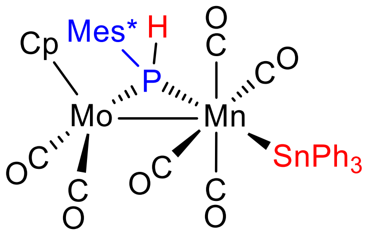

8b

Figure S37. IR spectrum of compound **8b** in toluene solution (* bands of isomer **9**).

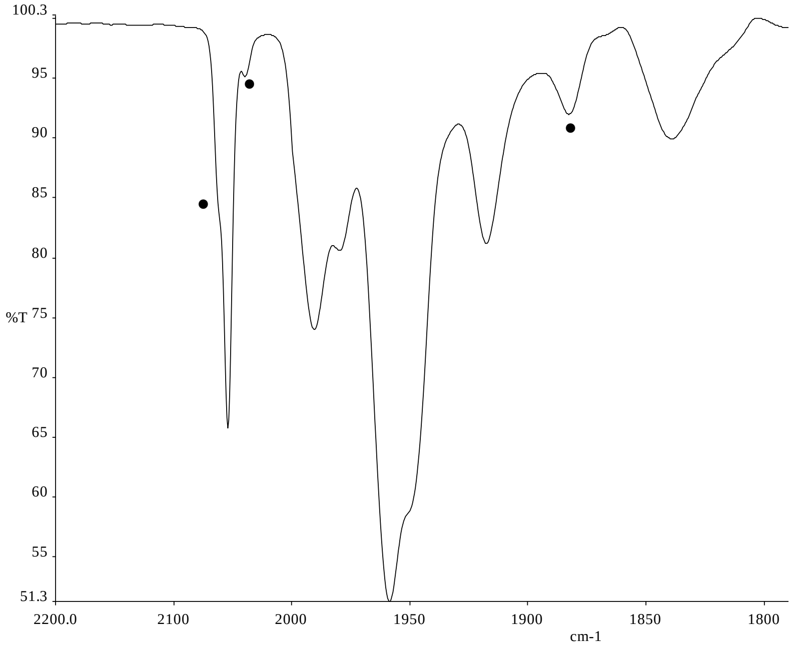

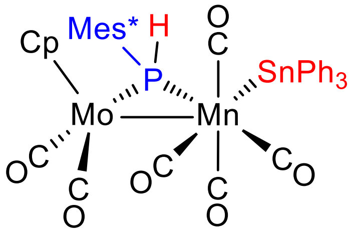

9

Figure S38. IR spectrum of compound **9** in toluene solution (• bands of isomer **8b**).

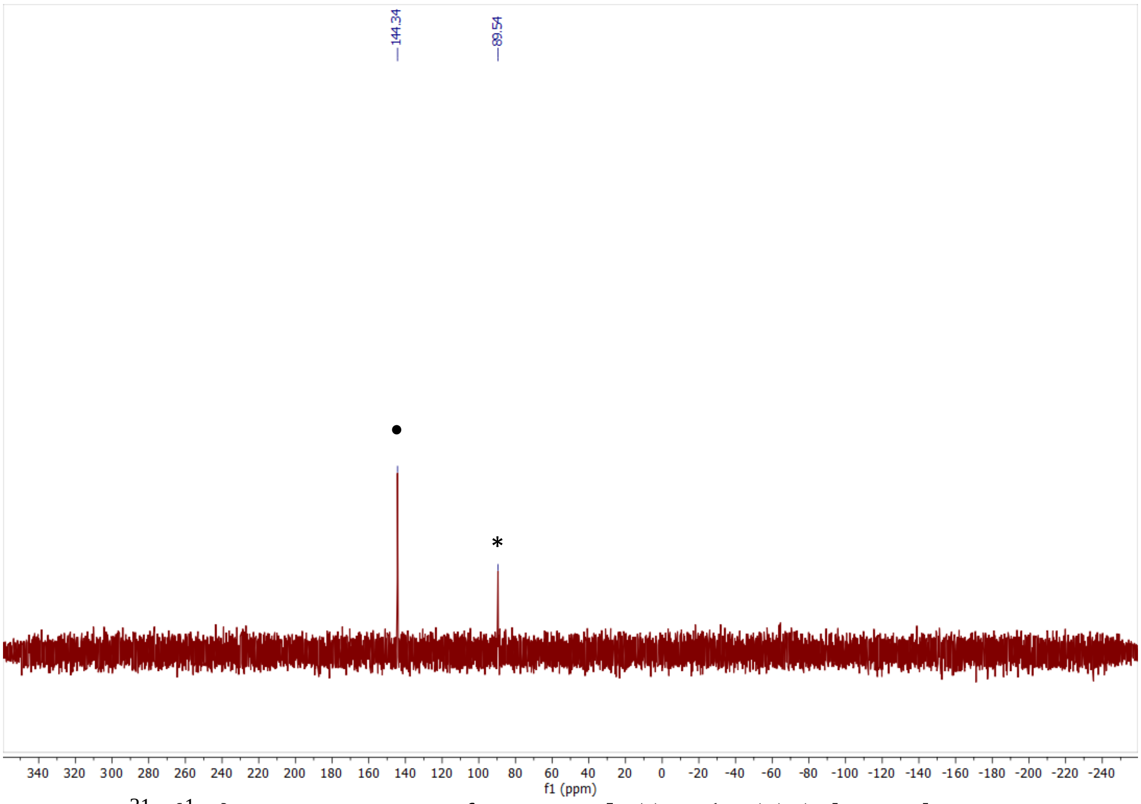

Figure S39. $^{31}P\{^{1}H\}$ NMR spectrum of isomers **8b** (•) and **9** (*) (toluene-d_8; equilibrium mixture).

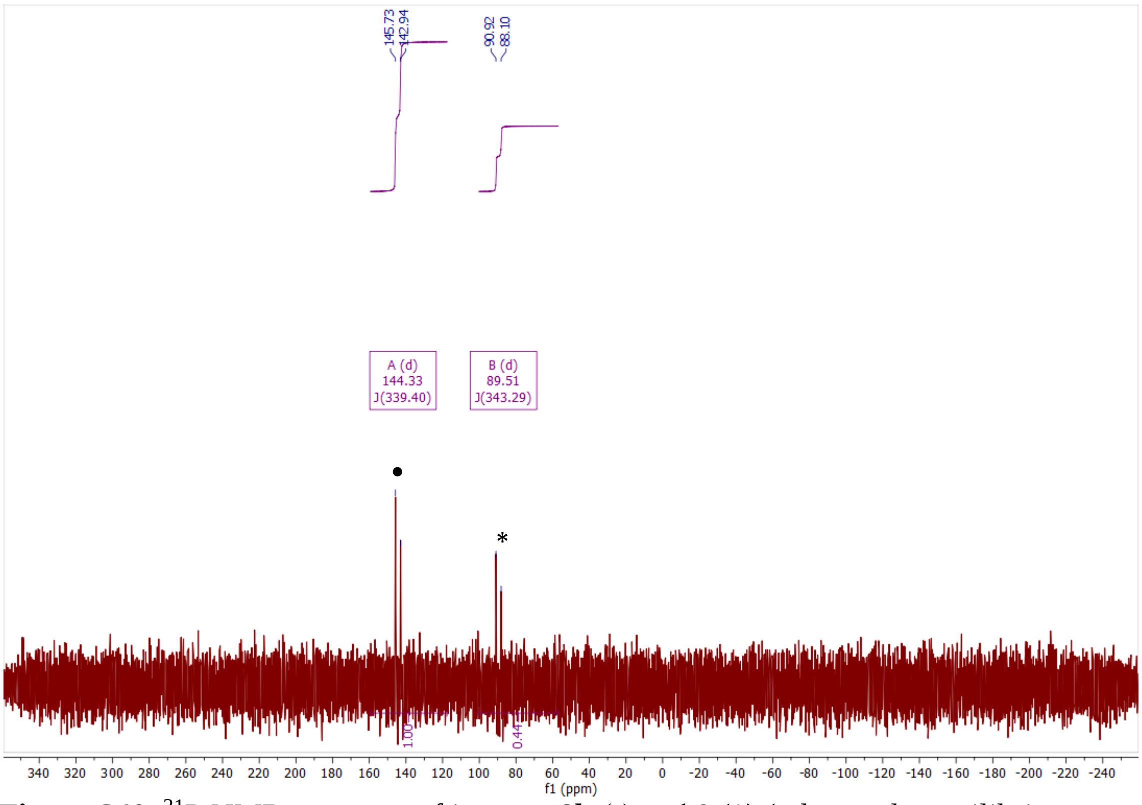

Figure S40. ^{31}P NMR spectrum of isomers **8b** (•) and **9** (*) (toluene-d_8; equilibrium mixture).

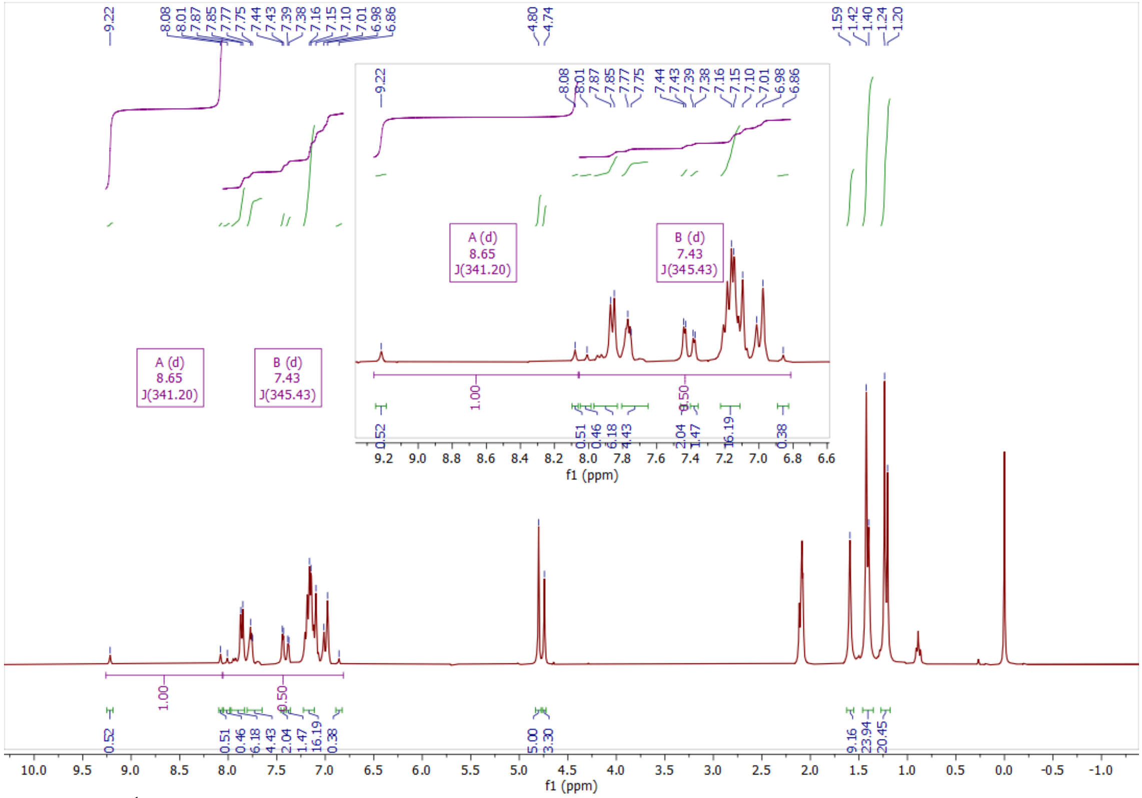

Figure S41. ^{1}H NMR spectrum of isomers **8b** (•) and **9** (*) (toluene-d_8; equilibrium mixture).

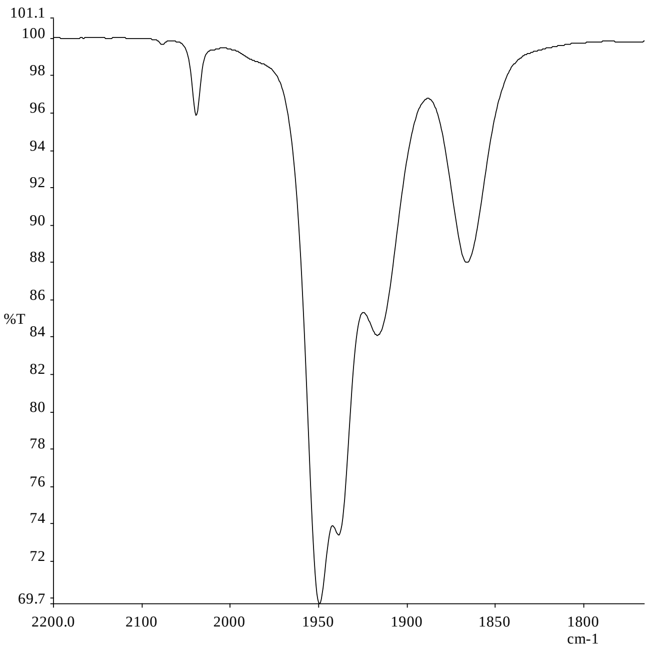

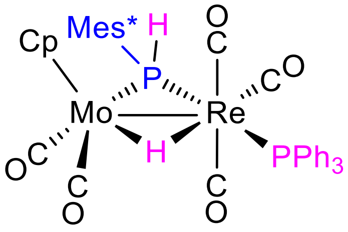

10

Figure S42. IR spectrum of compound **10** in dichloromethane solution.

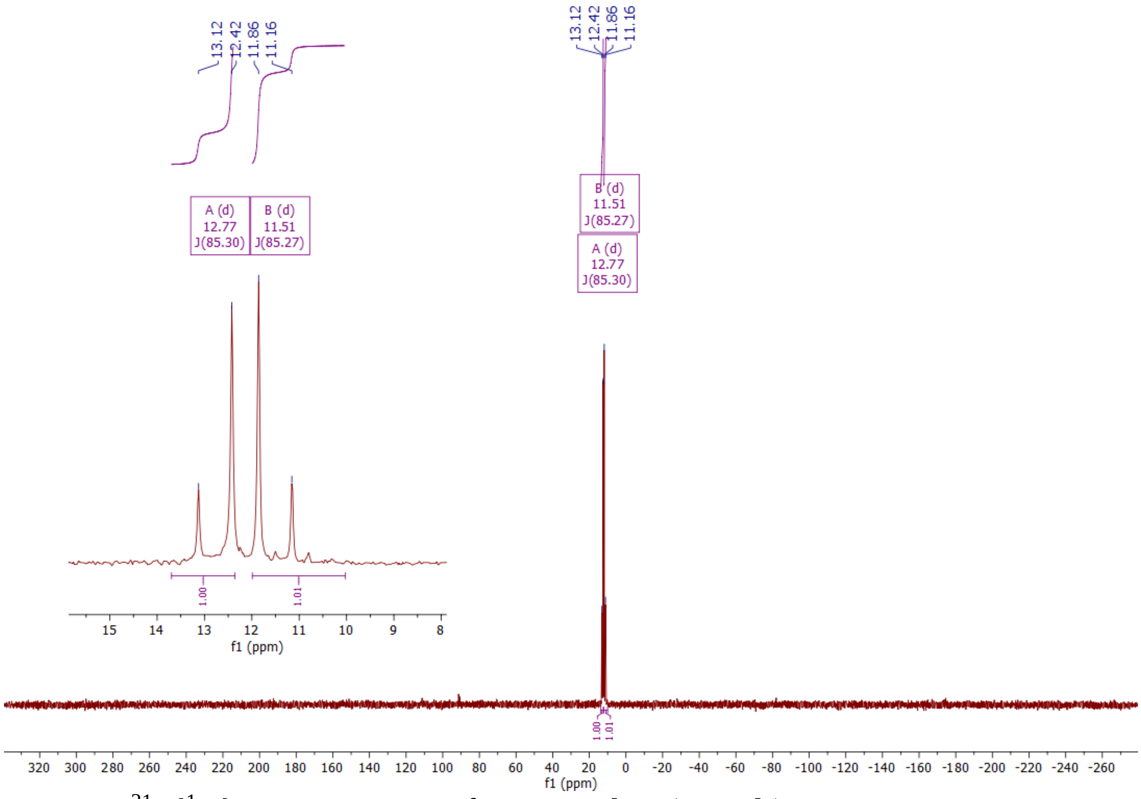

Figure S43. $^{31}P\{^{1}H\}$ NMR spectrum of compound **10** (CD_2Cl_2).

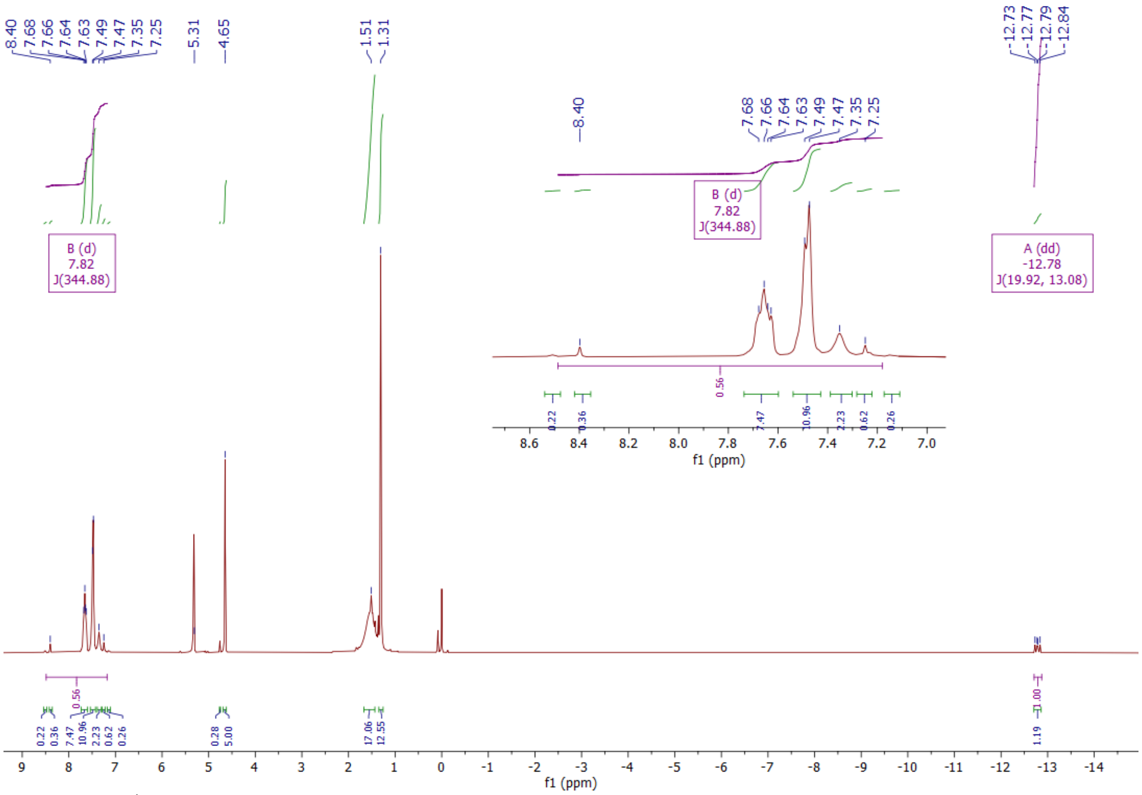

Figure S44. ^{1}H NMR spectrum of compound **10** (CD_2Cl_2).

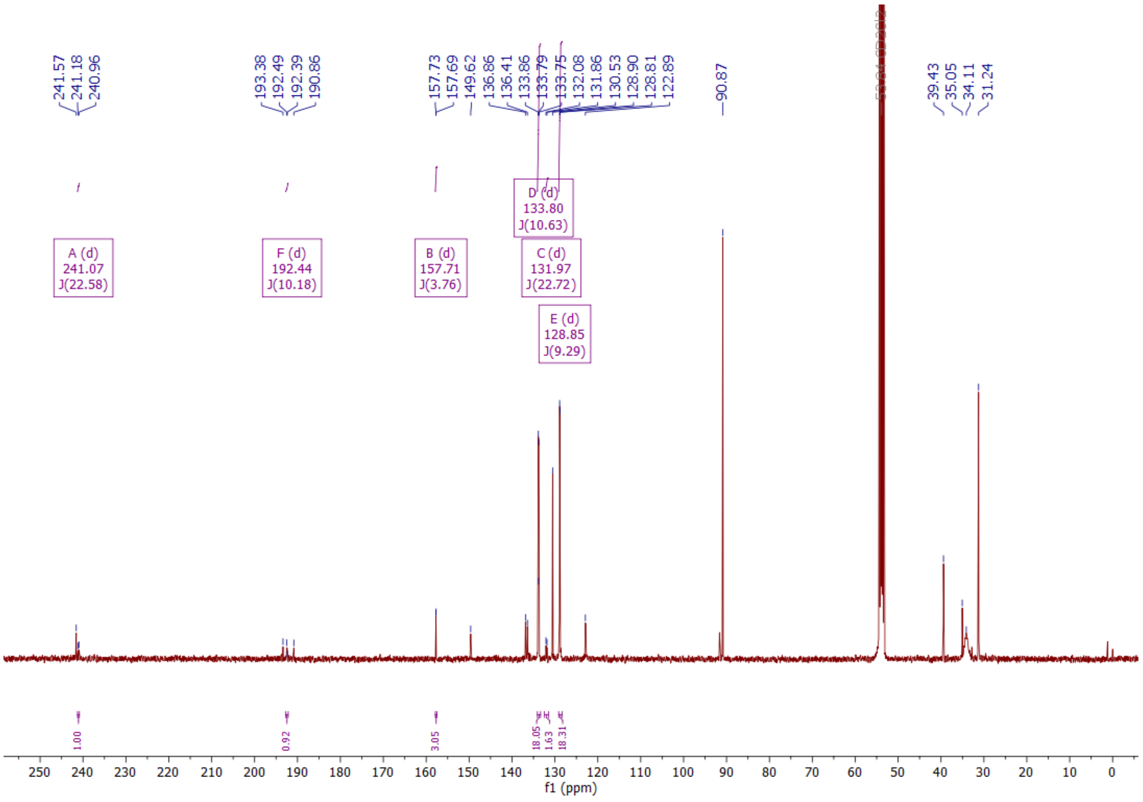

Figure S45. $^{13}C\{^{1}H\}$ NMR spectrum of compound **10** (CD_2Cl_2).

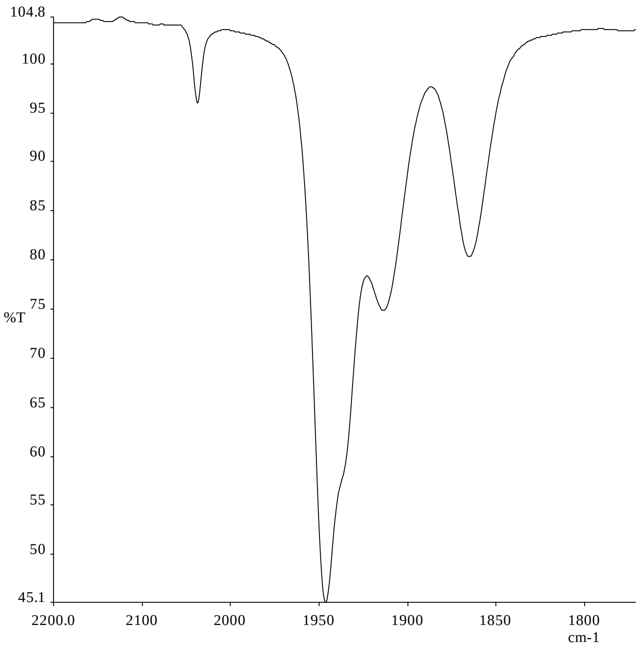

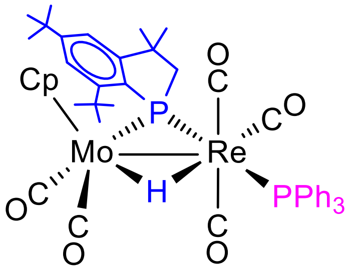

11

Figure S46. IR spectrum of compound **11** in dichloromethane solution.

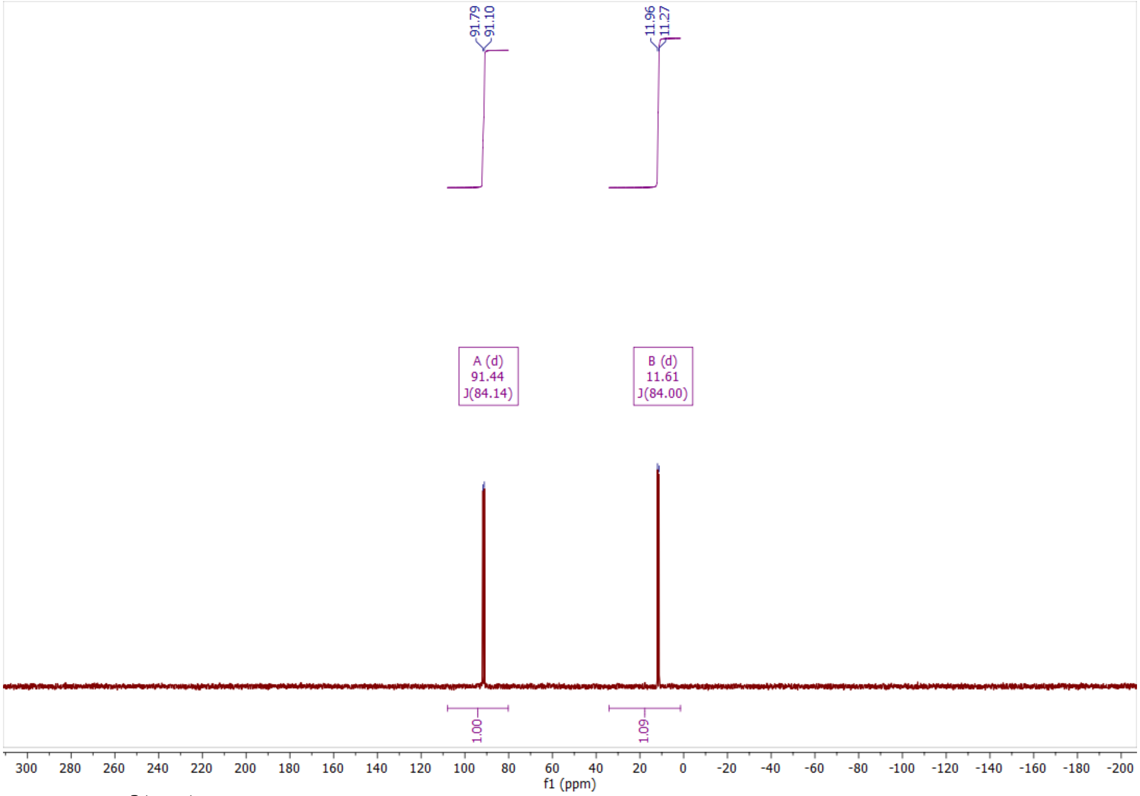

Figure S47. $^{31}P\{^{1}H\}$ NMR spectrum of compound **11** (CD_2Cl_2).

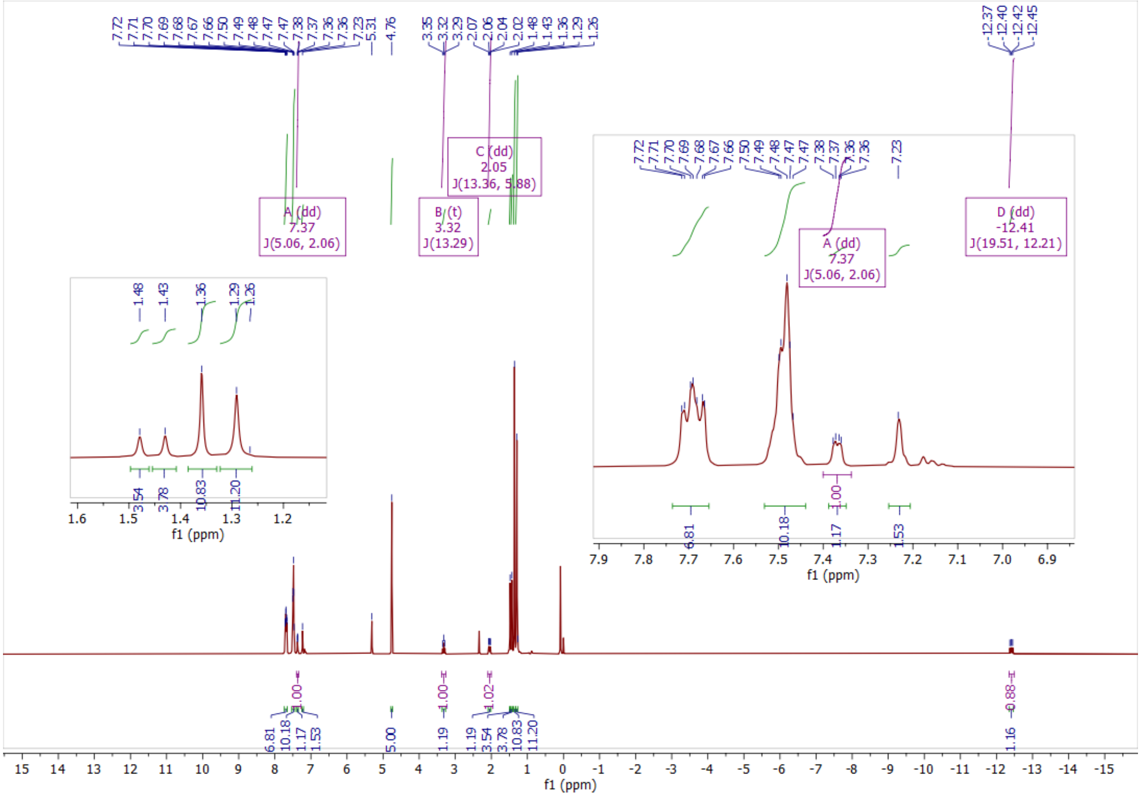

Figure S48. ^{1}H NMR spectrum of compound **11** (CD_2Cl_2).

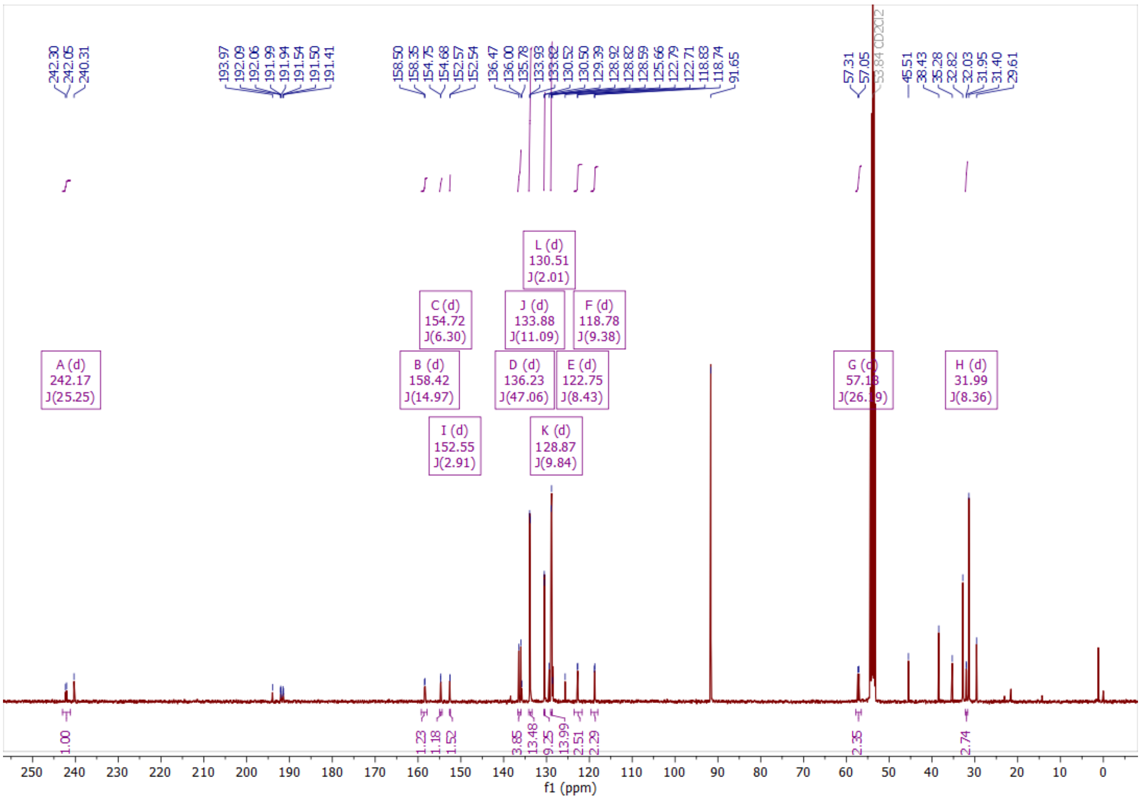

Figure S49. $^{13}C\{^{1}H\}$ NMR spectrum of compound **11** (CD_2Cl_2).

Figure S50. M06L-DFT optimized structures of model compound **1a-Ph** and the intermediates and transitions states following from its reaction with hydrogen, with their Gibbs free energies at 295 K (in kJ/mol, relative to **1a-Ph** + H_2) indicated between brackets, and most H atoms omitted for clarity.

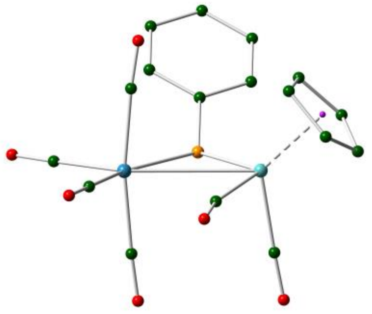	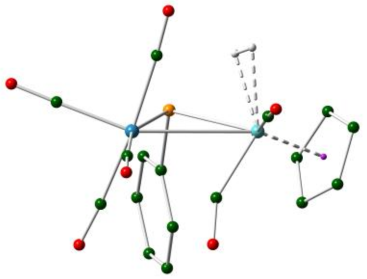
**1a-Ph**	**I1** (+97)
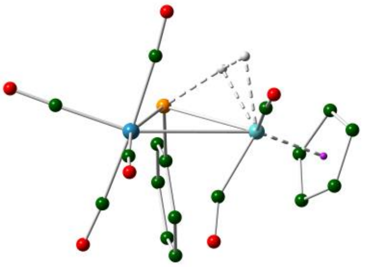	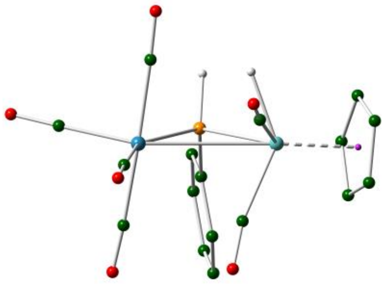
**TS1** (+108)	**I2** (-31)
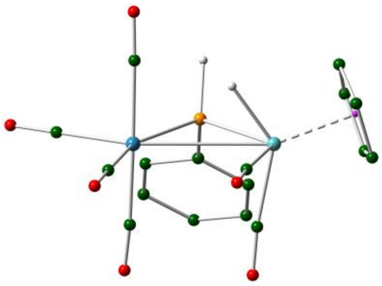	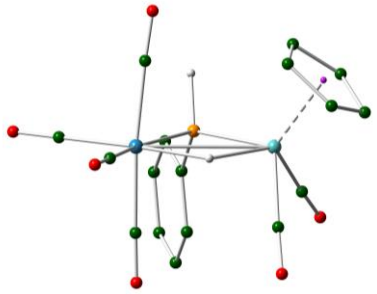
**TS2** (-16)	**2a-Ph** (-69)

Figure S51. Selected M06L-DFT molecular orbitals for model compound **1a-Ph**, with their energy (in eV) and main bonding character indicated below.

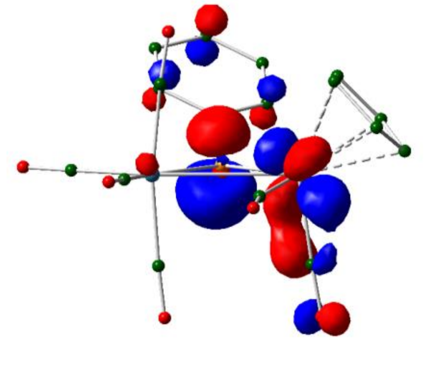	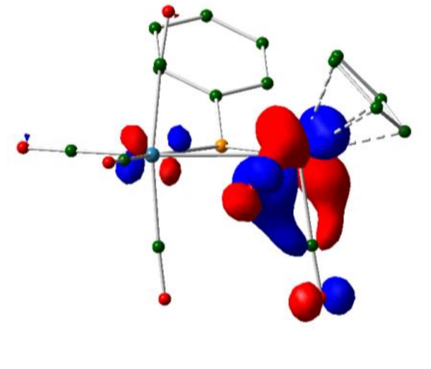
LUMO, –3.71eV, π*(Mo-P)	HOMO, –5.19eV, π(Mo-CO)
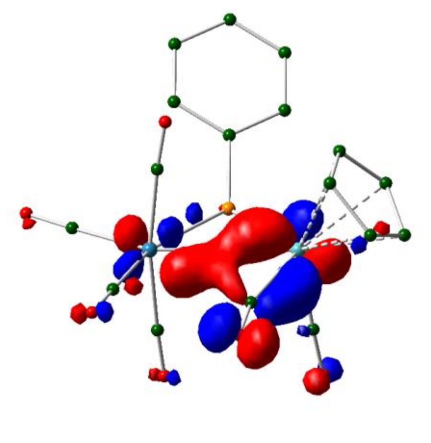	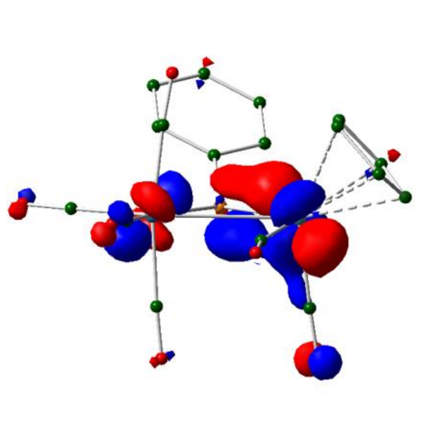
HOMO–1, –5.36eV, π(Mo-CO)	HOMO–2, –5.68eV, π(Mo-P)
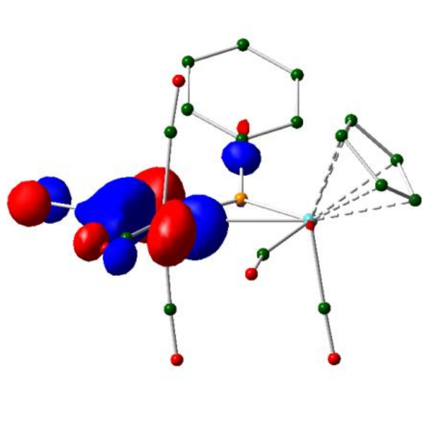	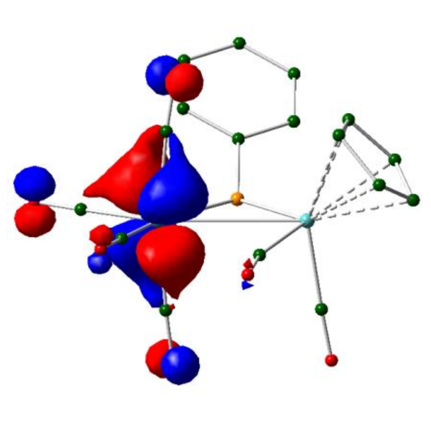
HOMO–3, –6.27eV, π(Re-CO)	HOMO–4, –6.61eV, π(Re-CO)
